# Arabidopsis TFL2/LHP1 Specifically Associates with Genes Marked by Trimethylation of Histone H3 Lysine 27

**DOI:** 10.1371/journal.pgen.0030086

**Published:** 2007-06-01

**Authors:** Franziska Turck, François Roudier, Sara Farrona, Marie-Laure Martin-Magniette, Elodie Guillaume, Nicolas Buisine, Séverine Gagnot, Robert A Martienssen, George Coupland, Vincent Colot

**Affiliations:** 1 Abteilung Entwicklungsbiologie der Pflanzen, Max Planck Institut für Züchtungsforschung, Cologne, Germany; 2 Unité de Recherche en Génomique Végétale, CNRS UMR8114, INRA UMR1165, Université d'Evry Val d'Essonne, Evry, France; 3 Mathématiques et Informatiques Appliquées, AgroParisTech, INRA UMR518, Paris, France; 4 Cold Spring Harbor Laboratory, Cold Spring Harbor, New York, United States of America; University of Cambridge, United Kingdom

## Abstract

TERMINAL FLOWER 2/LIKE HETEROCHROMATIN PROTEIN 1 (TFL2/LHP1) is the only *Arabidopsis* protein with overall sequence similarity to the HETEROCHROMATIN PROTEIN 1 (HP1) family of metazoans and S. pombe. TFL2/LHP1 represses transcription of numerous genes, including the flowering-time genes *FLOWERING LOCUS T* (*FT*) and *FLOWERING LOCUS C (FLC),* as well as the floral organ identity genes *AGAMOUS (AG) and APETALA 3 (AP3).* These genes are also regulated by proteins of the Polycomb repressive complex 2 (PRC2), and it has been proposed that TFL2/LHP1 represents a potential stabilizing factor of PRC2 activity. Here we show by chromatin immunoprecipitation and hybridization to an Arabidopsis Chromosome 4 tiling array (ChIP-chip) that TFL2/LHP1 associates with hundreds of small domains, almost all of which correspond to genes located within euchromatin. We investigated the chromatin marks to which TFL2/LHP1 binds and show that, in vitro, TFL2/LHP1 binds to histone H3 di- or tri-methylated at lysine 9 (H3K9me2 or H3K9me3), the marks recognized by HP1, and to histone H3 trimethylated at lysine 27 (H3K27me3), the mark deposited by PRC2. However, in vivo TFL2/LHP1 association with chromatin occurs almost exclusively and co-extensively with domains marked by H3K27me3, but not H3K9me2 or -3. Moreover, the distribution of H3K27me3 is unaffected in *lhp1* mutant plants, indicating that unlike PRC2 components, TFL2/LHP1 is not involved in the deposition of this mark. Rather, our data suggest that TFL2/LHP1 recognizes specifically H3K27me3 in vivo as part of a mechanism that represses the expression of many genes targeted by PRC2.

## Author Summary

Stable repression of gene expression is an important aspect of the developmental programs of higher organisms. In plants and animals, DNA is organized within chromatin, which contains at its core a set of evolutionarily conserved proteins called histones. These proteins can be modified for example by methylation or acetylation of lysines or phosphorylation of serines. Specific combinations of these histone modifications are interpreted by other chromatin proteins and thereby play essential roles in gene regulation. One such potential effector of the histone code in the flowering plant *Arabidopsis* is TERMINAL FLOWER 2/LIKE HETEROCHROMATIN PROTEIN 1 (TFL2/LHP1). Here we present highly detailed “epigenomic” maps that establish that TFL2/LHP1 associates with a subset of *Arabidopsis* genes that are marked by tri-methylation of Lysine 27 of histone H3. In plants and animals, an evolutionarily conserved complex called PRC2 deposits this mark. In *Drosophila* and mammals this modified histone is then read by another complex, called PRC1, to maintain the stable repression of genes. In *Arabidopsis* however, no PRC1 complex exists, and our results provide evidence that TFL2/LHP1 may fulfill a related function**.**

## Introduction

Spatial and temporal patterns of gene transcription are central to the developmental programs of plants and animals. Transcriptional repression plays a major role in creating and stabilizing these patterns. In plants, roles for transcriptional repression in reproductive development have been extensively studied. Proteins that repress transcription of genes that promote flowering or confer floral organ identity were identified by analysis of early-flowering mutants, and several of these proteins were predicted to play roles in chromatin regulation [1–7]. For example, CURLY LEAF (CLF) and EMBRYONIC FLOWER 2 (EMF2) are both required for the repression of floral organ identity genes and are homologues of *Drosophila* Enhancer of *zeste* (E[z]) and Suppressor of *zeste* 12 (Su[z]12), respectively, two core components of Polycomb repressive complex 2 (PRC2) [8–10]. In *Drosophila* and mammals, PRC2 catalyzes the tri-methylation of lysine 27 of histone H3 (H3K27me3) of nucleosomes widely located across target developmental genes [11,12]. This tri-methylation is then proposed to be recognized by the chromodomain of Polycomb, a central component of PRC1 [13], which maintains the stable transcriptional repression of target genes, although precisely how PRC1 operates is unclear. Despite the importance of PRC2 in the regulation of gene expression in plants, plant genomes do not appear to encode homologues of the metazoan PRC1 complex [8].

Here we focus on the *Arabidopsis* protein TERMINAL FLOWER 2/LIKE HETEROCHROMATIN PROTEIN 1 (TFL2/LHP1), which was implicated in the repression of flowering and chromatin regulation but is unrelated to known animal PRC1 or PRC2 components [5,6,14]. Mutations in *TFL2*/*LHP1* cause a range of developmental defects, including early flowering, reduced stability of the vernalized state, conversion of the shoot apical meristem to a terminal flower, curled leaves, and reduced root growth [5,6,14]. In addition, mutant *Arabidopsis* display constitutively altered glucosinolate levels and are unable to respond appropriately to heat-shock [15]. The early-flowering phenotype of *tfl2*/*lhp1* mutants results from increased expression of the floral promoter *FLOWERING LOCUS T (FT)* [6]. The instability of vernalization observed in *tfl2/lhp1* mutants is due to unstable repression of the floral repressor *FLOWERING LOCUS C (FLC)* [16,17], and it has been proposed that TFL2/LHP1 may have a PRC1-like role in the maintenance of PRC2-mediated repression of *FLC* [17]*.* Finally, the curled-leaf phenotype of *tfl2/lhp1* mutants is correlated with ectopic expression of the floral organ identity genes *AGAMOUS (AG)* and *APETALA3 (AP3)* [6,18]. These results suggest that TFL2/LHP1 represses transcription of genes that act during different stages of reproductive development. A more general role of TFL2/LHP1 in gene regulation is also suggested by the many misregulated genes detected in a partial transcriptome analysis of *tfl2/lhp1* seedlings [19].

TFL2/LHP1 is the only *Arabidopsis* protein that shows homology to HETEROCHROMATIN PROTEIN 1 (HP1) of metazoans and S. pombe [5,6], and based on the analysis of EST collections it appears to be widely conserved as a single-copy gene in plants. HP1 proteins are characterized by the presence of two conserved domains, the chromodomain and the chromo-shadow domain, and exist in multiple isoforms in metazoans [20]. As their names imply, the first members to be isolated (HP1a and b in *Drosophila,* HP1α and β in mammals) are enriched in heterochromatic regions. These HP1 isoforms, which are involved in the formation and maintenance of heterochromatin, but also participate in the regulation of heterochromatic and euchromatic genes, are believed to associate with target sites via the interaction of their chromodomain with di- or tri-methylated lysine 9 residues of Histone 3 (H3K9me2 or 3) [21–24], although alternative mechanisms of HP1 association likely exist [25–27]. In contrast to HP1a,b/ α,β isoforms, *Drosophila* and mammalian HP1c/γ isoforms appear to localize predominantly to euchromatic sites, where they either repress or activate genes through unknown mechanisms [28]. Similar to HP1c/γ, cytological localizations performed with transgenic plants indicate that TFL2/LHP1 is localized primarily in euchromatin, with little or no association with cytologically visible heterochromatin [19,29]. Taken together, these observations suggest that TFL2/LHP1 associates with genes present in euchromatin, and indeed such an association has recently been documented for *AG, AP3, FT, PISTILLATA* (*PI*), and *FLC* [16,17,30].

In order to gain deeper insight into the nature and chromosome-wide distribution of TFL2/LHP1 target sites and their associated histone marks, we have performed chromatin immunoprecipitation with antibodies that recognize epitope-tagged TFL2/LHP1 and hybridized the precipitated DNA to a DNA tiling array of the entire *Arabidopsis* Chromosome 4. We demonstrate that TFL2/LHP1 associates with hundreds of targets across this chromosome, the vast majority of which correspond to genes located within euchromatin. Furthermore, we show that although TFL2/LHP1 binds to H3K9me2, H3K9me3 and H3K27me27 peptides in vitro, in vivo TFL2/LHP1 associates almost exclusively and nearly co-extensively with H3K27me3. Moreover, the absence of noticeable changes in the distribution of H3K27me3 along Chromosome 4 in *lhp1* mutant plants indicates that TFL2/LHP1 is not involved in the deposition of this mark. Rather, TFL2/LHP1 specifically associates with H3K27me3 in an in vivo context, indicating that it is involved in a general mechanism of gene regulation mediated by PRC2.

## Results

### TFL2/LHP1 Binds Euchromatic Genes in Vivo

**Figure 1:**
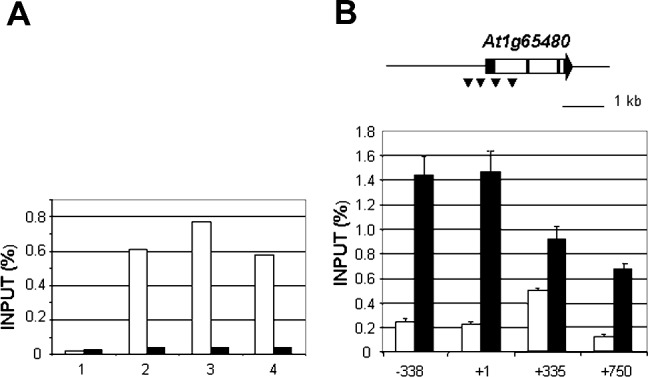
ChIP Assays with TFL2/LHP1:HA (A) ChIP was carried out with chromatin prepared from TFL2/LHP1:HA-expressing plants using anti-rat IgG control antibodies (1), or anti-HA antibodies developed in rabbit (2–4). Precipitated chromatin was eluted from the beads using low-pH buffer (1 and 2) or different amounts of HA-peptide (3 and 4). Precipitated DNA was tested via semi-quantitative PCR for presence of *FT (At1g65480)* proximal promoter, with PCR primers centered around position −388 from the transcriptional start (white bars) or *WRKY33 (At2g38470)* promoter with PCR primers centered around position −226 from the translational start (black bars). (B) ChIP samples prepared from TFL2/LHP1:HA (black bars) or FLC:HA-expressing plants (white bars) and immunoprecipitated with anti-HA antibodies were analyzed by quantitative PCR. PCR amplicons are indicated by triangles below the *FT* gene model. Data are based on three independent quantitative PCR experiments.

To define the genomic regions that TFL2/LHP1 directly associates with, chromatin immunoprecipitation (ChIP) experiments were conducted using transgenic plants expressing a functional HA-tagged version of the protein (see Materials and Methods). PCR analysis of ChIP DNA recovered from seedlings indicated a clear association of TFL2/LHP1 with the putative target gene *FT,* but not with the *WRKY33* gene taken as a negative control (Figure 1A). This association was not restricted to a single site at the *FT* locus, but spanned the ~700-bp region probed around the transcriptional start site (Figure 1B, black bars). This broad localization was not due to low resolution of the ChIP, because association of the MADS-box transcription factor FLC to the same region of the *FT* locus resulted in a well-defined and specific enrichment over the first intron, as reported previously [31,32] (Figure 1B, white bars).

**Figure 2:**
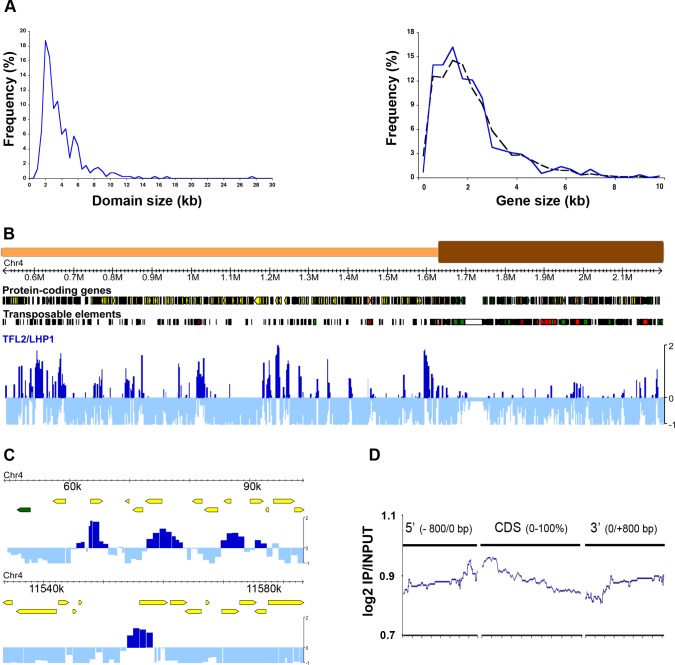
TFL2/LHP1 Associates with Small Euchromatic Gene Domains (A) Left panel: Size distribution of domains associated with TFL2/LHP1. Right panel: Size distribution of annotated genes present on Chromosome 4 (black dotted line) and of genes bound by TFL2/LHP1 (blue line). Note the absence of any skewing of the latter set compared to the whole chromosome set. (B) Genome browser view of a 1.7-Mb region of the short arm of Chromosome 4 (position 0.5 Mb to 2.2 Mb) centered on the distal boundary of the heterochromatic knob and showing the distribution of TFL2/LHP1 targets. Euchromatin and heterochromatin are depicted above the browser view as orange and brown cylinders, respectively. IP/INPUT ratios (log2) reporting significant TFL2/LHP1 association are marked in dark blue. (C) Genome browser representation of ChIP-chip data over several TFL2/LHP1 target loci. (D) Average value of IP/INPUT ratios along all TFL2/LHP1 target genes and 800 bp of flanking sequences. To accommodate for different gene lengths, positions within genes are indicated as percentage of total length. Highest mean ratios are located around the 5′ end of genes.

On the basis of these results, we performed a chromosome-wide analysis of TFL2/LHP1-associated regions by hybridizing ChIP DNA to a Chromosome 4 tiling DNA microarray. The array covers the 19-Mb *Arabidopsis* Chromosome 4 sequence as well as several other genomic regions in the form of 21,761 sequential 0.3–1.2-kb fragments ([33], see Materials and Methods). Chromosome 4 represents a good model for the 125-Mb *Arabidopsis* genome, which consists of five chromosomes that vary less than 2-fold in length and have similar sequence features. Importantly, unlike most array designs, which exclude repeats, these were included in order to interrogate repetitive as well as unique genomic sequences. Out of the ~21,000 tiles that could be analyzed reliably on the array, 1,713 showed robust association with TFL2/LHP1 (see Materials and Methods and Figure S1 for details of the analysis). In the majority of cases, TFL2/LHP1 association was observed over at least two contiguous tiles, as expected from the resolution provided by the array (0.3–1.2 kb) and the mean size of ChIP fragments (~800 bp). TFL2/LHP1 domains are 3.6 kb long on average, with the vast majority being less than 6.5 kb long (Figure 2A, left panel). Most domains are located evenly within the euchromatic parts of Chromosome 4 (Figures 2B and S2), consistent with cytological observations [19,29]. Of note, the 15 largest domains (10–27.5 kb) are all located within euchromatin, and may correspond to the granules observed over the diffuse, euchromatic-specific localization of TFL2/LHP1:GFP fusion proteins [19,29]. Strikingly, almost all TFL2/LHP1 domains coincide with genes and their flanking sequences (Figures 2 and S2). On average, preferential targeting of TFL2/LHP1 to proximal promoter regions and 5′ ends of target genes was observed (Figure 2D). Furthermore, the 603 TFL2/LHP1-target genes, which represent 15 % of the genes on the tiling array, showed no skewing towards any particular size class (Figure 2A, right panel). This observation contrasts with *Drosophila* HP1a, for which a clear preferential association was found with long genes, both in pericentric and nonpericentric regions [34], and towards the body and the 3′ end of transcription units [27]. Taken together, our results demonstrate that TFL2/LHP1 interacts with the chromatin of numerous individual transcriptional units that are located evenly along the repeat-poor regions of the *Arabidopsis* genome.

### Tandemly repeated genes are often targeted by TFL2/LHP1

**Figure 3:**
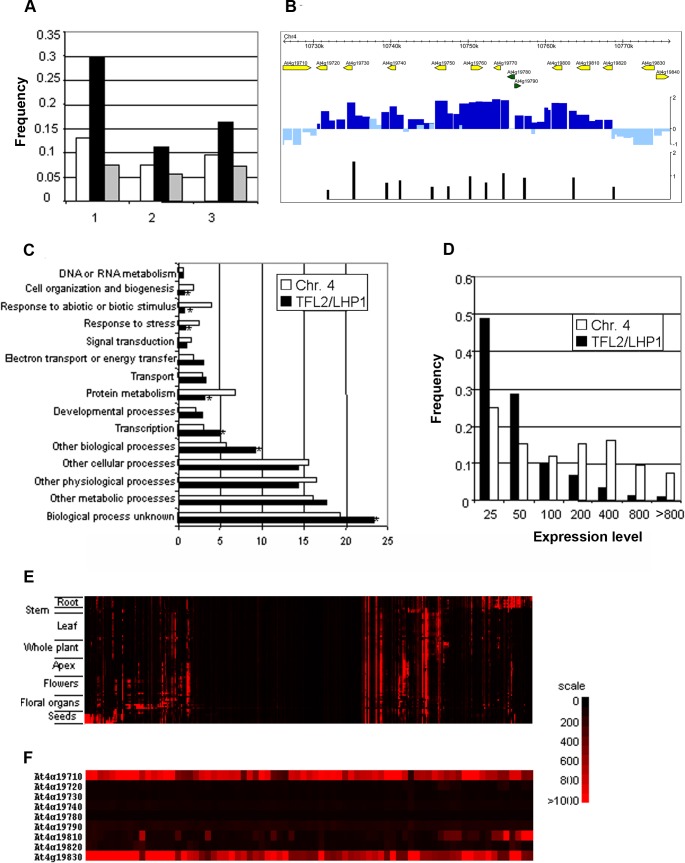
Analysis of TFL2/LHP1 Target Genes (A) Tandemly repeated genes are frequent targets of TFL2/LHP1. Percentage of TFL2/LHP1 targets among all Chromosome 4 genes (white bars), among tandemly repeated genes (black bars), and among genes that are part of segmental duplications (gray bars). To accommodate for possible cross-hybridization issues, the analysis was carried out considering all gene tiles (1), tiles with a single high-score BLAST hit (2), and tiles with one or two high-score BLAST hits. (B) Detailed analysis of TFL2/LHP1 association within a large array of tandemly repeated genes. Upper panel: Genome browser view of a 60-kb large region of Chromosome 4 encompassing the nine chitinase/glycosylase-18 genes (At4g19720–At4g19820), two LTR retrotransposon insertions (At4g19780 and At4g19790), and genes flanking the tandem array. Middle panel: ChIP-chip data presented as IP/INPUT ratios (log2). Lower panel: ChIP-PCR data using gene-specific primers presented as percentage of INPUT (black bars). (C) Gene ontology analysis of TFL2/LHP1 targets. Distribution of TFL2/LHP1 target genes (black bars) and all genes present on Chromosome 4 (white bars) into the different “biological process” categories as defined by TAIR. Note that the slight enrichment observed for “electron transport or energy pathways” is caused by the association of TFL2/LHP1 with the two large clusters of cytochrome P450 genes that are present on Chromosome 4. Asterisks indicate statistically significant differences (*p* < 0.01) between the two sets. (D) Global expression analysis of TFL2/LHP1 target genes. Median expression levels (horizontal axis) of TFL2/LHP1 target genes (white bars) and genes present of Chromosome 4 (black bars) were estimated from microarray data compiled at The Botany Beowulf Cluster (http://bbc.botany.utoronto.ca). The data were normalized using the standard MAS5.0 algorithm with a target value of 500. (E) Cluster representation [73] of the developmental series of expression data obtained from AtGenExpress (http://www.arabidopsis.org/servlets/TairObject?type=expression_set&id=1006710873) for 417 TFL2/LHP1 target genes (horizontal axis). Expression levels (vertical axis) are visualized as a heat map. (F) AtGenExpress data obtained for the nine tandemly repeated chitinase/glycosylase-18 genes and the two flanking genes. Genes are ordered as on the chromosome on the vertical axis. Expression profiles are indicated on the horizontal axis.

There are 249 sets of tandemly repeated genes on Chromosome 4 [35], corresponding to 679 genes. Thirty percent of these genes are present in the list of TFL2/LHP1 targets, suggesting that they are overrepresented. This was still observed when we excluded from the analysis all tiles that had more than one high-score BLAST hit against the *Arabidopsis* genome, thus ruling out potential cross-hybridization between highly related sequences as the sole cause of overrepresentation (Figure 3A). Furthemore, segmentally duplicated genes were not similarly enriched (Figure 3A). To explore further the association between TFL2/LHP1 and genes in large tandemly repeated gene clusters, the tandem array of nine glycosylase-18 genes present on Chromosome 4 was analyzed by ChIP-PCR (Figure 3B). Using gene-specific primer pairs, the association of TFL2/LHP1 with each gene of the tandem array was confirmed (Figure 3B). Moreover, IP/INPUT ratios measured by PCR were in agreement with the ChIP-chip data, demonstrating that cross-hybridization was not responsible for the observed broad distribution of TFL2/LHP1 over the glycosylase-18 gene cluster.

### Analysis of TFL2/LHP1 Gene Targets

The *Arabidopsis* Gene Ontology resource (http://arabidopsis.org) was used to categorize genes targeted by TFL2/LHP1 according to their functions. While most categories of “biological processes” were represented among TFL2/LHP1 targets, several categories were significantly enriched or depleted in comparison to their representation on Chromosome 4 (Figure 3C). Thus, protein metabolism (3.1% versus 6.7%), cell organization and biogenesis (0.7% versus 1.9%), response to stress (0.9% versus 2.5%), and environmental stimuli (0.8% versus 4.0%) are underrepresented, while transcription (4.9% versus 3.0%), other biological processes (9.2% versus 5.7%), and electron transport or energy pathways (3.0% versus 1.8%) are all overrepresented. These data demonstrate that TFL2/LHP1 is involved in the regulation of a wide range of biological processes and establish or confirm its association with key developmental regulators such as *AG* and *KNAT1,* which are located on Chromosome 4, as well as *AP3, FLC, LEAFY,* and *MEDEA* (*MEA*), which are located on other chromosomes but are represented on the tiling array (Tables S1 and S2).

To analyze the characteristics of TFL2/LHP1 targets further, expression levels were estimated using a comprehensive list of public microarray data compiled at AtGenExpress [36]. Over 80% of them have low or undetectable expression levels in most conditions tested, unlike the majority of other genes present on Chromosome 4 (Figure 3D and 3E). However, some targets are expressed to high levels in specific organs and developmental stages, such as seeds, flowers, apices, or roots (Figure 3E). Similarly, expression of most genes within the tandem array of glycosylase-18 genes is below detection level, unlike that of unrelated flanking genes, which are not associated with TFL2/LHP1 (Figure 3F). Finally, the set of genes with which TFL2/LHP1 is associated was compared with those previously shown to exhibit altered expression patterns in the *tfl2* mutant [19]. Of the 41 genes present on the tiling array that are differentially expressed in *tfl2–2,* nine are associated with TFL2/LHP1, and of these, seven show increased expression, including *AG* and *AP3*. The 32 remaining genes exhibit non-significant IP/INPUT ratios, suggesting that they are indirect targets of TFL2/LHP1 (data not shown).

### TFL2/LHP1 Associates with Genes That Are Marked by H3K27me3

**Figure 4:**
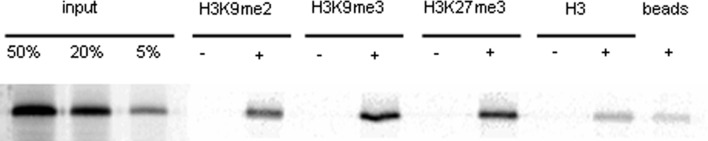
In Vitro Binding of TFL2/LHP1 to H3K27me3, H3K9me3, and H3K9me2 In vitro–translated and 35S-labeled TFL2/LHP1 was pulled down with either H3K9me2-, H3K9me3-, H3K27me3-, or H3-biotinylated peptides, or without peptide (beads). Purified protein was separated on SDS-PAGE and analyzed by autoradiography. Fractions of the input are shown as indicated. A representative autoradiogram of three independent experimental repetitions is shown.

By analogy with animal HP1a,b/α,β and the S. pombe homologue Clr4, it was originally proposed that TFL2/LHP1 recognizes chromatin marked by dimethylation of histone H3 lysine 9 [37]. However, both genetic and cytological evidence have since argued against an association of TFL2/LHP1 with H3K9me2 in plant cells [19,29,38]. The uncertainty concerning which chromatin mark(s) is recognized by TFL2/LHP1 prompted us to assess in vitro the binding of TFL2/LHP1 to histone H3 peptides carrying K9me2, K9me3, or K27me3 modifications. The latter two marks were tested because of cytological observations in *Arabidopsis* indicating that they are preferentially localized within euchromatin, as is TFL2/LHP1 [39–41]. The in vitro assay showed that TFL2/LHP1 binds significantly more to the three modified H3 peptides than to the unmodified one (Figure 4).

**Figure 5:**
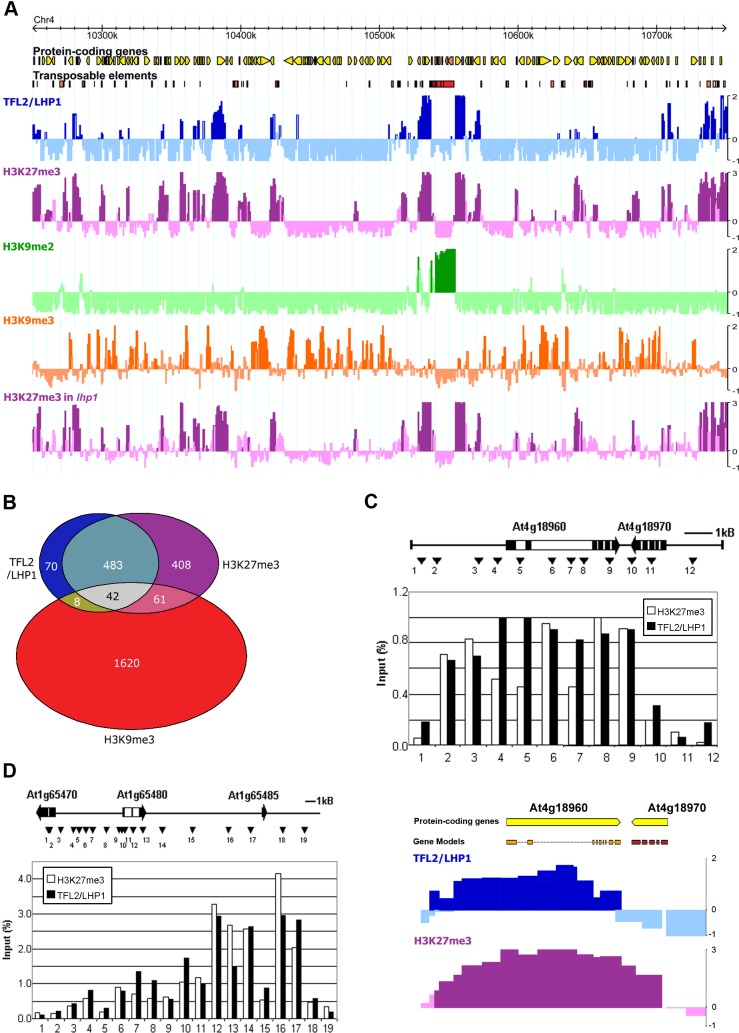
TFL2/LHP1 Associates Specifically with H3K27me3 (A) Genome browser view of a 500-kb region of Chromosome 4 (positions 10.25 Mb to 10.75 Mb) showing the co-extensive association of TFL2/LHP1 with H3K27me3, and the lack of overlap between H3K9me2, H3K9me3, and H3K27me3. IP/INPUT ratios (log2) reporting significant association are marked in dark colors. (B) Venn diagram showing the extent of overlap among genes that are associated with TFL2/LHP1, H3K27me3, or H3K9me3 (see also Table S9). (C) Scanning ChIP-PCR analysis of H3K27me3 (white bars) and TFL2/LHP1 (black bars) over the *AG* locus (At4g18960), and genome browser view of the corresponding epigenomic maps. (D) Scanning ChIP-PCR analysis of H3K27me3 (white bars) and TFL2/LHP1 (black bars) over the *FT* locus (At1g65480). In C and D, amplicons are indicated as triangles and numbered consecutively along the scanned regions. Data are based on three independent quantitative PCR experiments.

To explore further the interaction between TFL2/LHP1 and chromatin, ChIP-chip analyses were carried out using antibodies specific to these three histone H3 modifications. ChIP-chip performed with antibodies against H3K9me2 demonstrated that this chromatin mark is present almost exclusively over transposable elements and related repeats (Figures 5A and S2; Tables S3 and S4). Consistent with immunolocalization data, H3K9me2 is therefore particularly abundant in the repeat-rich pericentric regions of Chromosome 4 that are covered by the tiling array (Figure S2), as well as in the heterochromatic knob (Figure S2 and [42]). ChIP-chip analysis of H3K9me3 and H3K27me3 demonstrated that these two marks are detected mostly within the euchromatic parts of Chromosome 4 (Figures 5A and S2; Tables S5–S8), thus reinforcing cytological observations [43]. However, the higher resolution provided by the ChIP-chip analysis revealed that these two marks do not overlap significantly (Table S9) and are distributed over numerous small domains that typically cover one or two genes and their flanking sequences.

Little overlap was observed between TFL2/LHP1 and H3K9me3 localization, and in most cases this limited overlap results from closely juxtaposed domains, not from tight colocalization (Table S9). In contrast, 87.1 % (525/603) of TFL2/LHP1 gene targets are broadly marked by H3K27me3 (Figure 5B and Table S9). PCR scanning analysis of *AG* (At4g18960) and *FT* (At1g65480) confirmed the broad and coincidental localization of H3K27me3 over TFL2/LHP1 target genes (Figure 5C and 5D). However, the percentage of overlap between H3K27me3-marked genes and TFL2/LHP1 target genes is lower than the reciprocal (52.8 % versus 87.1 %; Table S9). Close examination of the epigenomic maps indicates that this results from a higher signal-to-noise ratio in the case of H3K27me3 (Figures 5A, 5C, and S2), which leads to H3K27me3 domains appearing somewhat larger in size than the corresponding TFL2/LHP1 domains. When this is taken into account, overlap between H3K27me3-marked genes and TFL2/LHP1 target genes increases from 52.8% to over 85%. In addition, some of the remaining differences between TFL2/LHP1 and H3K27me3 localization reflect insertion/deletion polymorphisms between the *Arabidopsis* accessions Landsberg *erecta* and Columbia (data not shown), in which the TFL2/LHP1 and the various histone modifications were mapped, respectively. We conclude therefore that TFL2/LHP1 and H3K27me3 are generally colocalized along the genome, and overlap at more than 85%–90% of sites at which they are present. Finally, ChIP-chip analysis of H3K27me3 was also performed in *lhp1* mutant seedlings. The H3K27me3 profiles were found to be very similar between wild type and *lhp1* (Figures 5A and S2). This result demonstrates that TFL2/LHP1 is not required for depositing H3K27me3, and suggests instead that TFL2/LHP1 is involved in interpreting this chromatin mark, which would account for the co-extensive distribution of TFL2/LHP1 and H3K27me3.

## Discussion

Using epigenomic profiling, we have established that TFL2/LHP1 is a euchromatic protein that mainly associates with genes marked by H3K27me3, rather than H3K9me2 or H3K9me3. Indeed, our ChIP-chip data demonstrate that TFL2/LHP1 associates with numerous and discrete H3K27me3-marked regions that are evenly distributed along euchromatin. These regions usually cover one or two genes and their immediate surroundings, with the notable exception of a few large domains, made up of tandemly repeated genes. Based on our analysis of Chromosome 4, we predict that approximately 15% of *Arabidopsis* genes are targeted by TFL2/LHP1.

### Association of TFL2/LHP1 with H3K27me3 Suggests Its Implication in PRC2-Mediated Control of Gene Expression

In *Drosophila* and mammals, silencing by Polycomb group proteins occurs through the deposition of H3K27me3. This modification is carried out by the SET domain histone H3 methlytransferase E(z),which is present in the evolutionarily conserved PRC2. Once deposited, this chromatin mark is thought to be recognized by PRC1, which maintains transcriptional repression by still-unknown mechanisms. The recognition of H3K27me3 by PRC1 is believed to occur via the chromodomain of the Polycomb protein, based on in vitro studies [13]. However, in plants, no homologues of Polycomb or other components of PRC1 have so far been identified, thus raising the question of the nature of the protein that might recognize the H3K27me3 mark.

Recent reports have shown that mutations in one of the three genes encoding *E(z)* homologues in *Arabidopsis*, *CLF*, *SWINGER* (*SWN*), and *MEA*, reduces H3K27me3 levels at PRC2 target genes, demonstrating that PRC2 generates the same chromatin mark in plants and animals [9,44–47]. These results provide strong evidence that the function of PRC2 is conserved between plants and animals. Several genes whose transcriptional repression requires PRC2 are also regulated by TFL2/LHP1. Thus, *clf* mutants show a curled-leaf phenotype similar to *tfl2/lhp1* as well as ectopic expression of *AG* and *AP3* [48]. Mutations in other PRC2 components also cause developmental defects related to those of *tfl2/lhp1,* although these defects are considerably enhanced in some PRC2 mutants. For example, VERNALIZATION 2 (VRN2) and EMF2, which are homologues of the PRC2 component Su(z)12 in animals, also control processes regulated by TFL2/LHP1. VRN2 is critical for the maintenance of transcriptional repression of *FLC* after vernalization [49] and leads to an enrichment of H3K27me3 over *FLC* [17,44]. Stable repression of *FLC* expression also requires TFL2/LHP1 [16,17]. By associating with chromatin that is marked with H3K27me3, TFL2/LHP1 resembles the Polycomb component of animal PRC1, which participates in the transcriptional repression of genes targeted by PRC2. Such a role has in fact already been proposed, notably based on the observation that TFL2/LHP1 is required to maintain stable repression of *FLC* following vernalization [17,41]. In other respects, however, TFL2/LHP1 differs from Polycomb since it is not required for the repression of all genes regulated by PRC2, as suggested by the milder phenotypes of *tfl2/lhp1* mutants compared to mutants in which PRC2 function is affected. Furthermore, *tfl2*/*lhp1* mutants lack fertilization or seed development defects [5,6,14], which contrasts with the severe reproductive defects observed with the PRC2 mutants *fertilization independent endosperm* (*fie*), *fertilization independent seed 2* (*fis2*), and *mea* [50]. These differences may be explained by the small fraction of genes marked by H3K27me3 that may not be associated with TFL2/LHP1. Alternatively, the repressive function of TFL2/LHP1 may be partially redundant with other protein complexes involved in PRC2-mediated regulation. Such a hypothesis would be consistent with the observation that loss of PRC1 but not PRC2 genes has occurred repeatedly during the evolution of metazoans [51].

While the chromodomain of *Drosophila* Polycomb recognizes H3K27me3, the chromodomains of *Drosophila* and mouse HP1 proteins have low affinity for H3K27me3 peptides and bind to H3K9me2 and H3K9me3 [52–54]. However, none of the mouse Polycomb homologs accumulate at pericentric heterochromatin, which is enriched in H3K9me3, despite displaying in vitro affinity towards both H3K9me3 and H3K27me3 [55]. Similarly, whereas TFL2/LHP1 is specifically associated with H3K27me3 in vivo, it binds to H3K9me2, H3K9me3 and H3K27me3 in vitro. These observations demonstrate that predictions of protein binding based on in vitro assays do not always hold up as other factors clearly come into play in vivo.

If TFL2/LHP1 takes the place of Polycomb in a plant-specific complex functionally related to PRC1 of animals, other proteins present in this complex may provide additional affinity to H3K27me3. Alternatively, a second, yet unidentified chromatin mark could codistribute with H3K27me3 and be specifically recognized by other proteins that are part of the complex. Likewise, the ubiquitination mark of H2A lysine 119 is recognized by the *Drosophila* PRC1, although here, deposition of the mark appears to be a downstream event of H3K27 trimethylation [56–58]. Both scenarios provide an explanation for the fact that a proportion of the TFL2/LHP1 target genes does not appear to be upregulated in *tfl2* and that some mutants of PRC2 have more severe phenotypes than *tfl2/lhp1* mutants, since loss of TFL2/LHP1 function could be buffered by other members of the complex [8,19,59].

As HP1, TFL2/LHP1 probably recruits accessory repressive proteins via an interaction with its chromo-shadow domain. One of the best characterized interaction partners of HP1 is the SUV(39)1/2 histone methyltransferase [20,28]. Interaction between the two proteins is thought to be required for the spreading of heterochromatin in animals. So far, there is no genetic evidence in *Arabidopsis* connecting TFL2/LHP1 to any of the SUV(39)1/2 histone methyltransferases [16]. In contrast to *lhp1,* single mutants of the ten *Arabidopsis* homologs of *SUV(39)1/2* do not interfere with the stable repression of *FLC* after vernalization [16]. However, genetic redundancy between the *Arabidopsis SUV(39)1/2* homologs has not been excluded. Nevertheless, *suvh4* and *suvh2* single mutants affect gene silencing [38,39] and *PAI1* gene silencing is SUVH4 dependent but does not require a functional TFL2/LHP1 [38].

A connection between transcriptional repression, TFL2/LHP1 recruitment, and di- as well as trimethylation of H3K9 and H3K27, was previously suggested for the *FLC* gene, which is silenced during vernalization [16,17,60]. The finding that TFL2/LHP1 binds H3K9me2 or 3 and H3K27me3 in vitro may corroborate this hypothesis. However, our in vivo data do not support this as a general model for the TFL2/LHP1 mode of function since we found no correlation between TFL2/LHP1 targets and H3K9me2 or H3K9me3. Nevertheless, *FLC* is stably repressed only in plants exposed to vernalization, and TFL2/LHP1 might interact with H3K9me2 and H3K9me3 only under specialized conditions such as these.

Finally, although 20%–30% of *Arabidopsis* genes are associated with some degree of DNA methylation [61–63], we found no correlation between TFL2/LHP1 or H3K27me3 localization and DNA methylation patterns (data not shown). This observation reinforces the notion that DNA methylation does not seem to have a widespread role in regulating the expression of genes in plants [50,63].

### TFL2/LHP1 Target Genes and Regulation by PRCs

In *Drosophila*, repression by Polycomb group proteins occurs through their association with specific DNA sequences. These Polycomb response elements (PREs) include many conserved short motifs, but exhibit no overall sequence similarity [13]. In *Drosophila*, H3K27me3 methylation extends well beyond these PREs, whereas binding of most PRC2 and PRC1 components, with the notable exception of Polycomb, is restricted to the PREs themselves. In mammals however, no PRE has been identified and PRC1 and PRC2 binding extends over larger regions, leaving open the possibility that Polycomb group proteins are recruited by a different mechanism [13]. Whether plants resemble mammals or *Drosophila* in this respect remains to be determined. Interestingly, TFL2/LHP1 binding and H3K27me3 marking were often more pronounced around the proximal promoter and 5′ coding region of target genes (Figures 2D and S2). In *AG,* key regulatory motifs are located within the largest intron, for which maximal association with TFL2/LHP1 and H3K27me3 was detected (Figure 5C). Taken together, these results suggest that transcriptional regulatory elements may provide entry points for PRC2-dependent trimethylation of H3K27 in *Arabidopsis*.

Tandemly repeated genes are frequent targets of TFL2/LHP1, suggesting that they induce higher order structural changes of their chromatin that provide a mark for TFL2/LHP1 recruitment. Conversely, the fact that these genes are duplicated and highly related may require particular control of their expression and TFL2/LHP1 could therefore actively participate in a dosage compensation mechanism. The observation that genes that are part of segmental duplications are not similarly overrepresented favors the first scenario.

### *Arabidopsis* Does Not Contain a Heterochromatic Isoform of HP1

TFL2/LHP1 is the only *Arabidopsis* homologue of HP1, and therefore our demonstration that TFL2/LHP1 is located almost exclusively in euchromatin, which supports previous cytological data [19,29,64], indicates that *Arabidopsis* does not contain a functional homologue of the heterochromatic isoforms of HP1. One of the functions of metazoan HP1 a/α and b/β isoforms is the stabilization of condensed pericentromeric heterochromatin. Since *Arabidopsis* clearly has such heterochromatin, the machinery involved in its stabilization must be different from its metazoan counterpart. There is a relative flexibility as to which histone modifications are associated with heterochromatin in different organisms. The predominant histone H3 modification in mammalian heterochromatin is H3K9me3, a mark that is not enriched in the analogous heterochromatic regions in *Arabidopsis,* but shows a diffuse distribution, both at the cytological [39,43] and molecular (this work) levels. In contrast, the H3K9me2 mark is strongly associated with heterochromatic regions in plants, while in mammals it is not enriched in centromeres but is rather correlated with transcriptional silencing of euchromatic genes [65]. An exception is the inactive X chromosome, which carries both H3K9me2 and H3K27me3 [66]. DNA methylation and the occurrence of the H3K9me2 mark are correlated over repeated sequences in plants and animals, and both are therefore highly enriched in heterochromatin [67]. However, loss of these two marks does not always lead to a loss of cytologically visible heterochromatin [68,69]. Recently, VARIANT IN METHYLATION 1 (VIM1) was identified because of its involvement in the maintenance of centromeric heterochromatin. VIM1 is concentrated at chromocenters and possesses both histone and methyl–cytosine interaction domains [70]. If VIM1 provides the glue that stabilizes heterochromatin in *Arabidopsis,* then TFL2/LHP1 would not have been required for this function in the ancestor of land plants. This, together with the fact that TFL2/LHP1 is euchromatic and that many of the animal HP1 proteins also localize to euchromatin, suggests an evolutionary scenario in which the ancestral role of HP1 was to repress euchromatic genes, with its heterochromatic role being a derived character.

## Materials and Methods

### Plant material.

TFL2/LHP1:HA transgenic plants were produced in the Landsberg *erecta* (L*er*) accession. The *35S::TFL2/LHP1:HA* construct used in this study complements the *tfl2/lhp1* mutation, indicating that the addition of the HA epitope does not impair functionality of the protein. Of 22 scored T1 plants, six showed a wild-type growth habit, 15 showed intermediate phenotypes between wild type and *tfl2* and only one showed the terminal flower phenotype. Wild-type and *lhp1–1* mutant plants used for the ChIP-chip analysis of histone H3 methylation marks were of the Columbia (Col) accession. The *lhp1–1* mutation was originally isolated in the Wassilewskija accession [5] and was introgressed into Columbia through six crosses. The *TFL2/LHP1:HA* C-terminal fusion gene was generated by amplifying a full-length *TFL2/LHP1* cDNA with primers TFL2_HA_F, 5′-CCATGAAAGGGGCAAGTGGTGCT-3′ and TFL2_HA_R, 5′-CATTAAGTAGTGGGAGAGTCACCGG-3′ that were flanked by Gateway recombination sites, and by recombining the PCR product into a Gateway entry clone (Invitrogen, http://www.invitrogen.com). The *TFL2/LHP1* cDNA was then recombined into a modified pJawohl binary destination vector to produce *35S::TFL2/LHP1:HA*. Transformation was carried out as described [31].

### ChIP assays.

ChIP assays were carried out as described [31,71] using 10-d-old seedlings grown in liquid and the following antibodies: anti-HA (H6908, Sigma, http://www.sigmaaldrich.com), anti-H3K9me2 (07–441; Upstate, http://www.upstate.com), anti-H3K9me3 (07–442, Upstate), and anti-H3K27me3 (07–449, Upstate). Primers used for ChIP-PCR are described in Table S10. The FT promoter sequence of the accession L*er,* which carries a 1.5-kb deletion compared with the publicly available Col sequence, has been deposited at EMBL. No signal was detected in mock-antibody precipitations, whereas a weak signal was found in nontarget regions. Since the background bands are at the limit of detection for quantitative PCR and the more sensitive end-point PCR analysis, we expressed our ChIP results as percentage of input fraction and not as fold-enrichment over negative controls. Quantitative PCR values were obtained by averaging results of at least two independent experiments. Note that no significant difference in H3K27me3 distribution was observed between untransformed and *35S::TFL2/LHP1:HA* transgenic seedlings, indicating that overexpression of TFL2/LHP1:HA does not affect H3K27me3 distribution (Figure S3).

### Chromosome 4 tiling array.

*Arabidopsis* Chromosome 4 tiling microarray was designed from the entire sequence of Chromosome 4 and comprised 21,405 printed features, each consisting of 0.3–1.2-kb PCR product amplified with sequential primer pairs along Chromosome 4. An additional 356 amplicons of similar size were printed that cover 36 genes of interest and neighboring sequences located on the other four chromosomes [63]. Over 50% of tiles represent single-copy regions as identified by BLAST analysis of sequential 100-bp windows against the entire *Arabidopsis* genome sequence [33].

### ChIP-chip analysis.

ChIP-chip analyses of TFL2/LHP1:HA and the three histone marks H3K9me2, H3K9me3, and H3K27me3 were performed on two biological replicates, except for that of H3K27me3 in the *lhp1* mutant, which was carried out once. DNA recovered after ChIP (IP fraction) and directly from input chromatin (INPUT) was differentially labeled and hybridized in classical dye-swap experiments to correct dye biases, as previously described [42]. Arrays were scanned (GenePix 4000A scanner, Axon Instruments) and fluorescence was quantified using the software GenePix Pro. Data obtained for each array were stored as GenePix reader (gpr) files. No background was subtracted, and raw data (base 2 logarithm of median feature pixel intensity) corresponding to the 635-nm and 532-nm wavelength channels were extracted for each hybridized array from the corresponding gpr file. Manually flagged spots (−100) were excluded from the analysis.

Since the INPUT and IP samples differ substantially, array-by-array normalization such as loess cannot be applied. Instead, normalization between arrays was performed based on the properties of dye-swaps to remove technical biases. Let *Y_ij_* be the signal of the sample labeled with the dye *j* on the array *i*. Given that the second array is a technical replicate of the first one, the distribution of *Y*_21_ (respectively *Y*_22_) should be close to that of *Y*_12_ (respectively *Y*_11)_. In practice, the relationship between *Y*_21_ and *Y*_12_ is linear but it is not the identity function. The parameters of the two linear models are estimated by *Y*_21_ = *a* + *bY*_12_ + *N*(0,σ^2^) and *Y*_22_ = *c* + *dY*_11_ + *N*(0,σ^2^), and these estimates are used to define the normalized IP and INPUT values of the second array relative to the first one: *Y*_21_ = (*Y*_21_ – *a*)/*b* and *Y*_22_ = (*Y*_22 –_ *c*)/*d*. For each sample and for each tile, the values of the two arrays of the dye-swap are then averaged.

For each dye-swap, IP/INPUT ratios were analyzed using a two-step procedure, as follows (see also Figure S1). First, a hybridization threshold was defined by modeling the distribution of average IP values for the ~12,000 tiles of the array that are devoid of repeated sequences, and that cannot therefore lead to cross-hybridization [33]. To this end, truncated and non-truncated Gaussian mixture models were applied [72], with a component number varying from one to five. Using the Bayesian information criterion, models with two or three components were systematically selected. We then interpret the components to characterize significant IP signals. The selected model is often a mixture with two well-separated components. In this case, tiles classified in the component with greatest mean according to the maximum a posteriori (MAP) rule are declared to have significant IP values. When the number of components is three, the component with the lowest mean is well separated from the others and characterizes background hybridization. Consequently, tiles classified in the other two components according to the MAP rule are declared to have significant IP values.

In a second step, all IP values above the hybridization threshold were retrieved from the list of ~21000 IP values in order to analyze the corresponding IP/INPUT ratios. To define so-called “enriched” tiles, a similar procedure based on mixture models was used. Given that the Chromosome 4 tiling array contains both unique and repeated sequences, the unavoidable carry over of total genomic DNA leads to possible significant IP values for these sequences. As a matter of fact, most selected models contained two or three components, and the component with the lowest mean was found to correspond mainly to tiles with highly repeated sequences. When the selected model is a mixture with two well-separated components, tiles classified in the component of greatest mean according to the MAP rule are declared enriched. When the number of components is three, we note that the two components with the lowest means are well separated from the last. As above, tiles classified in the component of greatest mean according to the MAP rule are declared enriched. In the case of the model with four components (H3K9me2, replicate 2), the component with the second-lowest mean is fully included within the component with the lowest mean. Tiles classified in the two components of greatest means according to the MAP rule are declared enriched (Figure S1).

Lists of tiles with IP/INPUT ratios reporting significant enrichment were compared between biological replicates (Table S11; Figure S2). Overlap ranged from 94.8% for TFL2/LHP1 to nearly 100% for H3K9me3, reflecting a higher signal-to-noise ratio in the case of most methylation marks compared to TFL2/LHP1. Selected tiles common to the two replicates were manually curated to remove “singletons,” as these were not expected from the average size of chromatin fragments (800 bp) and the resolution provided by the array (934 bp on average). Indeed, the majority of singletons were found to result from cross-hybridization. These curated lists were used for the final analysis (Tables S1, S3, S5, and S7). Estimation of specificity (false positive) and sensitivity (false negative) was achieved experimentally by performing ChIP PCR on randomly chosen loci (Figure S4). Tiles that cover annotated genes by at least 50 bp were called “genic” and were used to obtain the genes lists of Tables S2, S4, S6, and S8.

### In vitro transcription/translation of TFL2/LHP1 and peptide pull-down assays.

The TFL2/LHP1 cDNA was recombined into a pTNT vector (L5610; Promega, http://www.promega.com) with the Gateway cassette cloned in the XbaI restriction site (generous gift of S. Jang, Cologne). This plasmid allows in vitro transcription of *TFL2/LHP1* by the T7 polymerase. Plasmid DNA (1 μg) was transcribed and the resulting RNA translated using the TNT Quick Coupled kit (L1170, Promega) following the manufacturer's instructions. For peptide pull-down assays, 50 ng of biotinylated histone H3 peptides, that were either methylated on K27 (aa 21–44; 12–565, Upstate), methylated on K9, or unmethylated (aa 1–21; 12–430 and 12–403, Upstate) were incubated with 10 μl of Dynabeads M-280 Streptavidin (Dynal/Invitrogen) in PBS for 2 h at 4 °C and blocked with 0.25% BSA in PBS for 30 min. The beads were washed three times with binding buffer (20 mM Tris-HCl pH 7.9, 0.2 mM EDTA, 1mM DTT, 0.2 mM PMSF, and 20% glycerol) containing 200 mM KCl and mixed with 5 μl of in vitro translated protein and 100 μl of binding buffer for 60 min at 4 °C. After washing five times with binding buffer containing 500 mM KCl, the bound proteins were eluted in SDS loading buffer, resolved by SDS-PAGE, and visualized using a phosphorimager.

## Supporting Information

Figure S1 Mixture Models Used to Identify IP/INPUT Values Reporting Significant Enrichment Distribution of IP and IP/INPUT values are indicated for each biological replicate in the left and right panels, respectively. Gaussians are indicated in navy blue, light blue, red, and yellow in order of increasing means. Hybridization and IP/INPUT thresholds are indicated as vertical bars.
(244 KB PDF)

Figure S2 Genome Browser View of Epigenomic Maps The entire set of epigenomic maps that was produced in this work is presented in consecutive 1-Mb panels. Biological replicates are shown below each other. IP/INPUT ratios (log2) reporting significant association are marked in dark colors.
(4.4 MB PDF)

Figure S3 Correlation of H3K27me3 in Untransformed and 35S::TFL2/LHP1:HA Transgenic Seedlings PCR was carried out on ChIP samples obtained with anti-H3K27me3 antibodies, using 23 different primer pairs. IP/INPUT ratios are plotted against each other. The data show that over-expression TFL2/LHP1 does not affect H3K27me3 distribution.
(46 KB PDF)

Figure S4 Confirmation of ChIP-Chip Results by ChIP-PCR ChIP-PCR was carried out based on 33 randomly selected tiles that showed by ChIP-chip either no association with TFL2/LHP1 and H3K27me3, or association with both. Fifteen out of the 17 tiles declared “negative” and 13 out of 16 tiles declared “positive” by ChIP-chip were validated by PCR in independent ChIP experiments. Except in one case, discrepancies between ChIP-chip and ChIP-PCR concerned tiles with IP/INPUT values in the ChIP-chip analysis of TFL2/LHP1and H3K27me3 that were just below or above the positive/negative thresholds.
(65 KB PDF)

Table S1 List of Tiles Associated with TFL2/LHP1 (239 KB XLS)

Table S2 List of Genes Associated with TFL2/LHP1 (61 KB XLS)

Table S3 List of Tiles Associated with H3K9me2 (335 KB XLS)

Table S4 List of Genes Associated with H3K9me2 (53 KB XLS)

Table S5 List of Tiles Associated with H3K27me3 (389 KB XLS)

Table S6 List of Genes Associated with H3K27me3 (91 KB XLS)

Table S7 List of Tiles Associated with H3K9me3 (681 KB XLS)

Table S8 List of Genes Associated with H3K9me3 (165 KB XLS)

Table S9 Extent of Overlap between TFL2/LHP1 Target Genes and Genes Marked by H3K9me2, H3K9me3 or H3K27me3 (32 KB DOC)

Table S10 List of PCR Primers (30 KB XLS)

Table S11 Extent of Overlap between Biological Replicates (27 KB DOC)

### Accession Numbers

Accession numbers for the ArrayExpress (http://www.ebi.ac.uk/arrayexpress) data discussed in this paper are A-MEXP-602, array design and E-MEXP-951, experimental data.

The European Molecular Biology Laboratory database (EMBL) (http://www.ebi.ac.uk/embl) accession number for the FT promoter sequence in L*er* is AM492685.

A browser-based interface to the data is available at the following website: http://dynagen.ijm.jussieu.fr/research/tools/repet/gbrowse/arabidopsis.

## Abbreviations

*AG*: *AGAMOUS*
*AP3*: *APETALA 3*
ChIP: chromatin immunoprecipitation
*FLC*: *FLOWERING LOCUS C*
*FT*: *FLOWERING LOCUS T*
HP1: HETEROCHROMATIN PROTEIN 1
H3K27me3: histone H3 trimethylated at lysine 27
IP: immunoprecipitation
PRC: Polycomb repressive complex
PRE: Polycomb response element
TFL2/LHP1: TERMINAL FLOWER 2/LIKE HETEROCHROMATIN PROTEIN 1
