# Supplementary material for: Arabidopsis TFL2/LHP1 Specifically Associates with Genes Marked by Trimethylation of Histone H3 Lysine 27
Source: PLoS Genet. 2007 Jun 1;3(6):e86. doi: 10.1371/journal.pgen.0030086 (PMC1885283; doi:10.1371/journal.pgen.0030086)

Turck, Roudier et al. FIGURE S1

TFL2/LHP1

Replicate1

Hybridization threshold = 7.2545

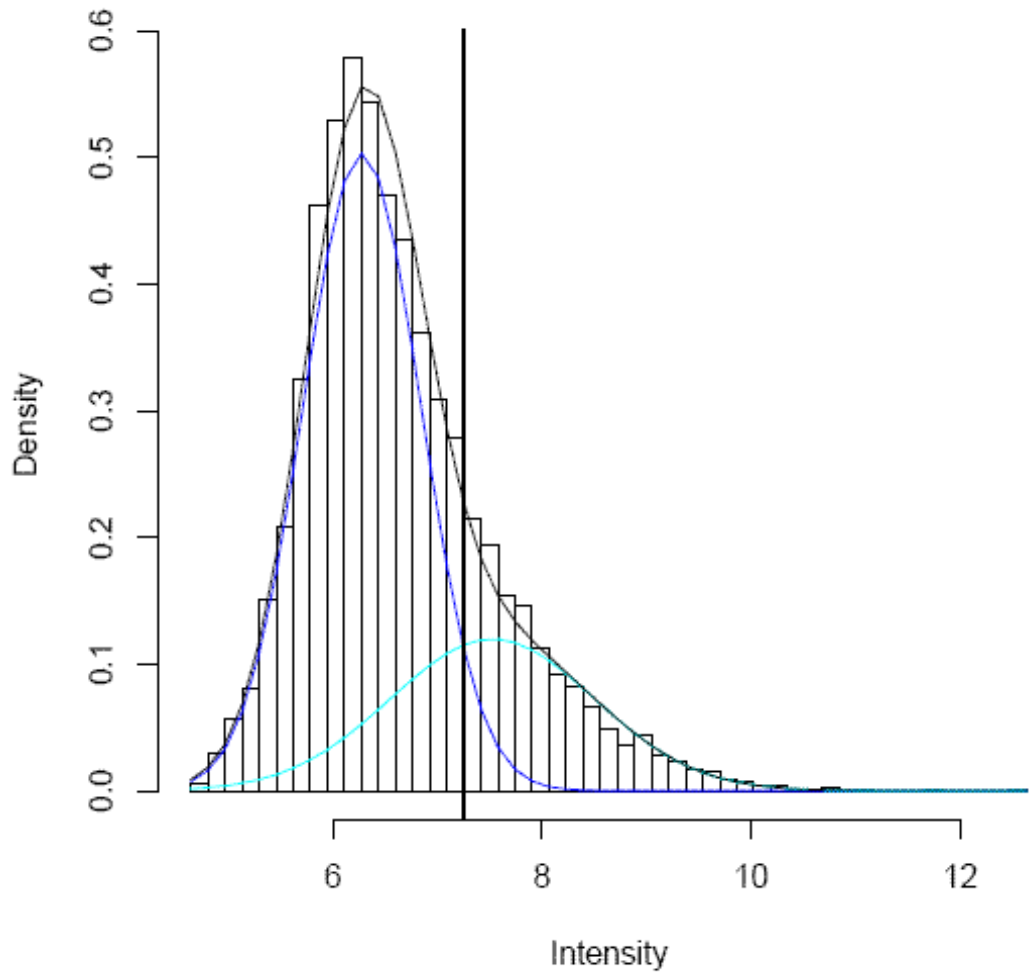

Ratio threshold = - 0.5831

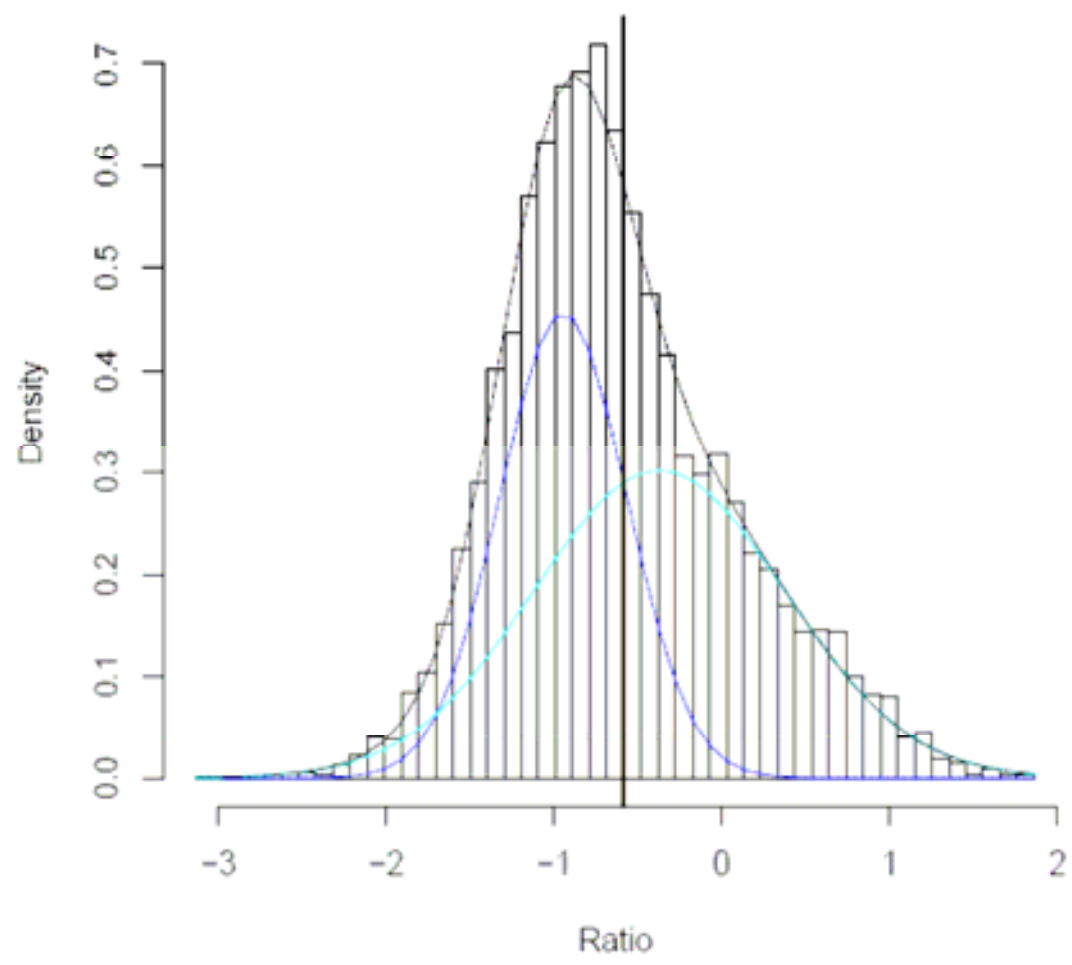

Replicate2

Hybridization threshold = 7.1465

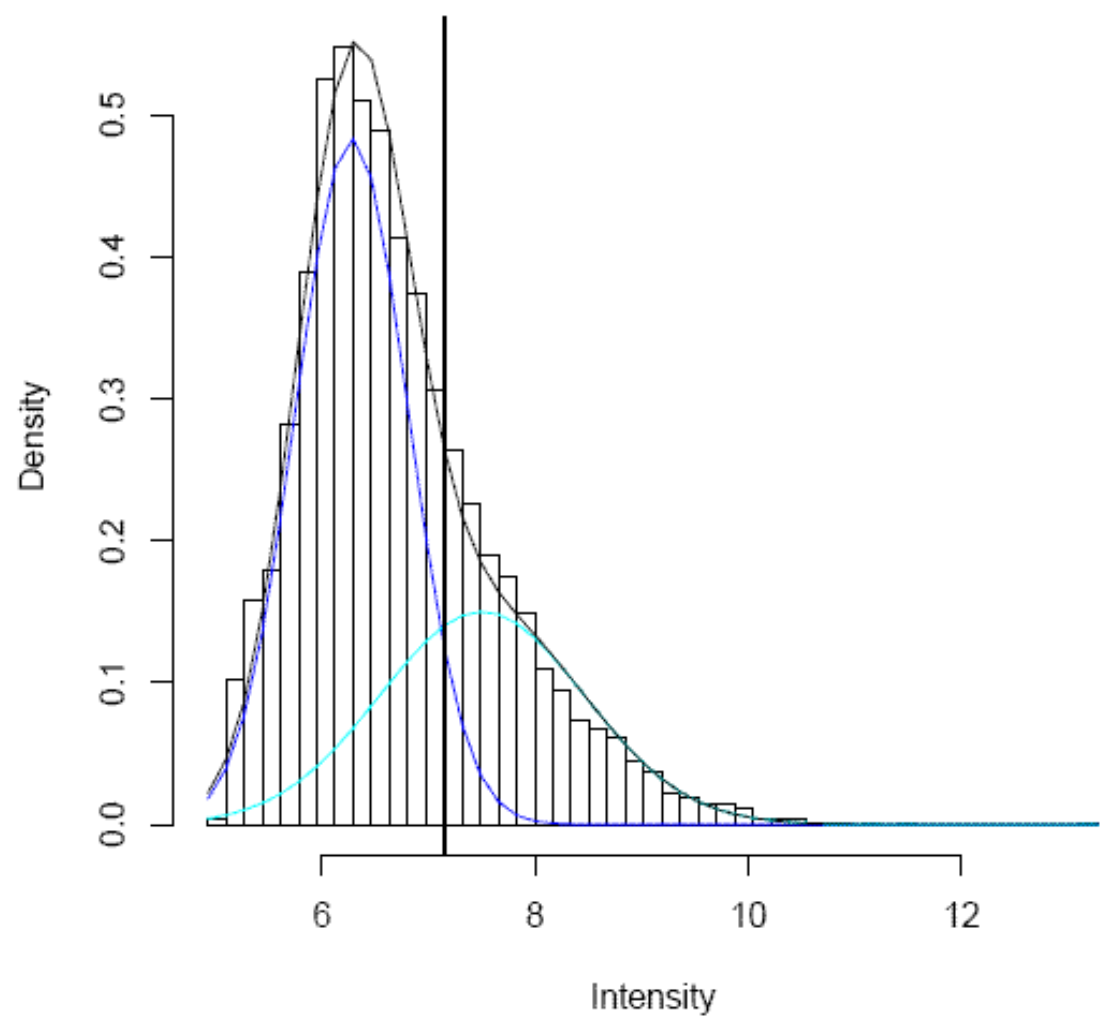

Ratio threshold = - 0.2132

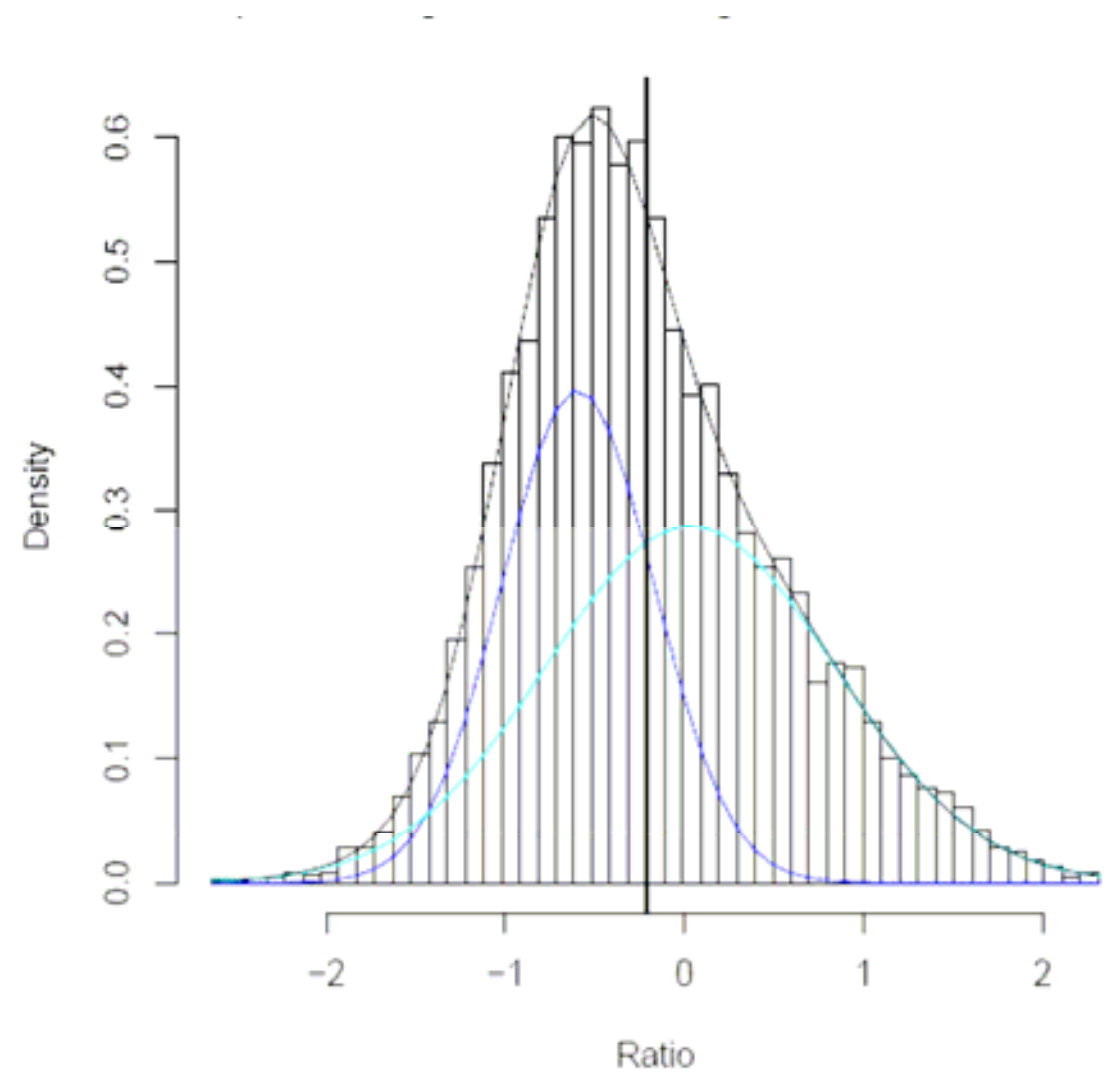

H3K27me3 (WT)

Hybridization threshold = 9.1438

Ratio threshold = - 0.4785

Replicate1

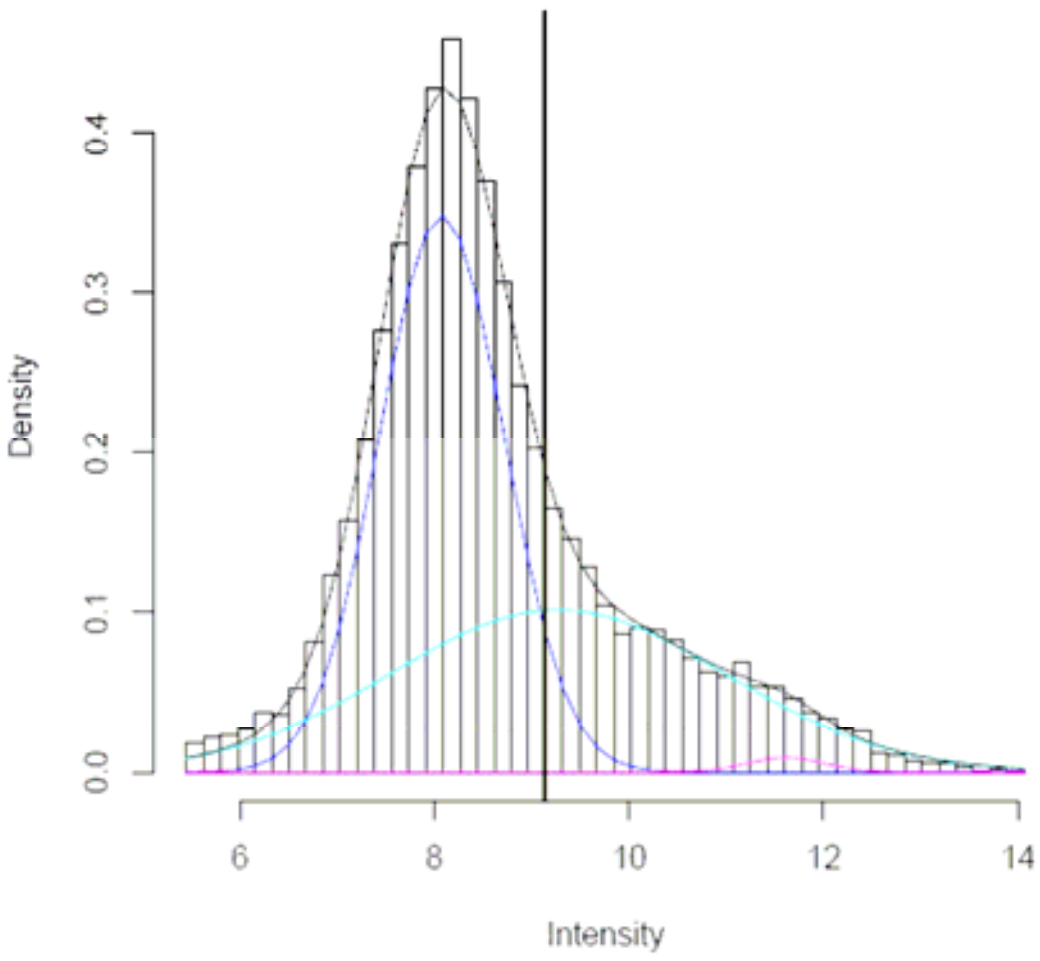

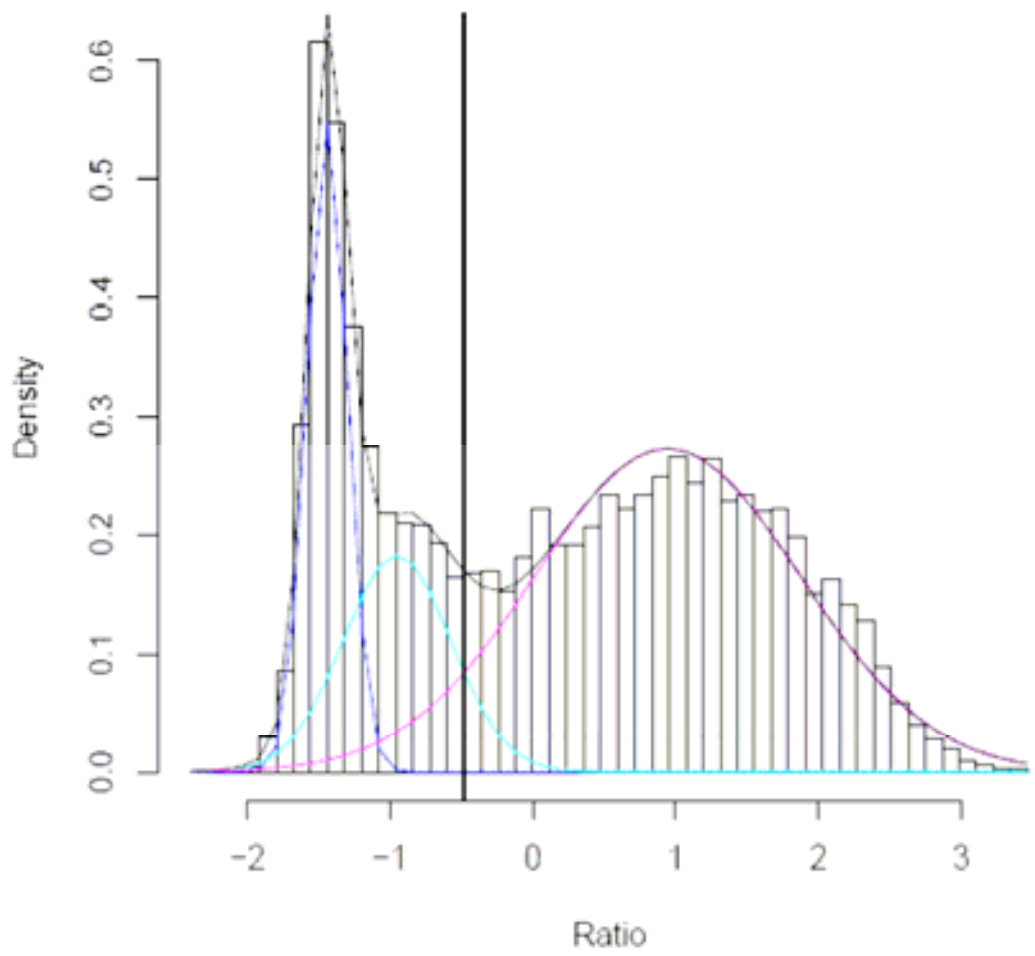

Hybridization threshold = 8.666

Ratio threshold = - 0.4017

Replicate2

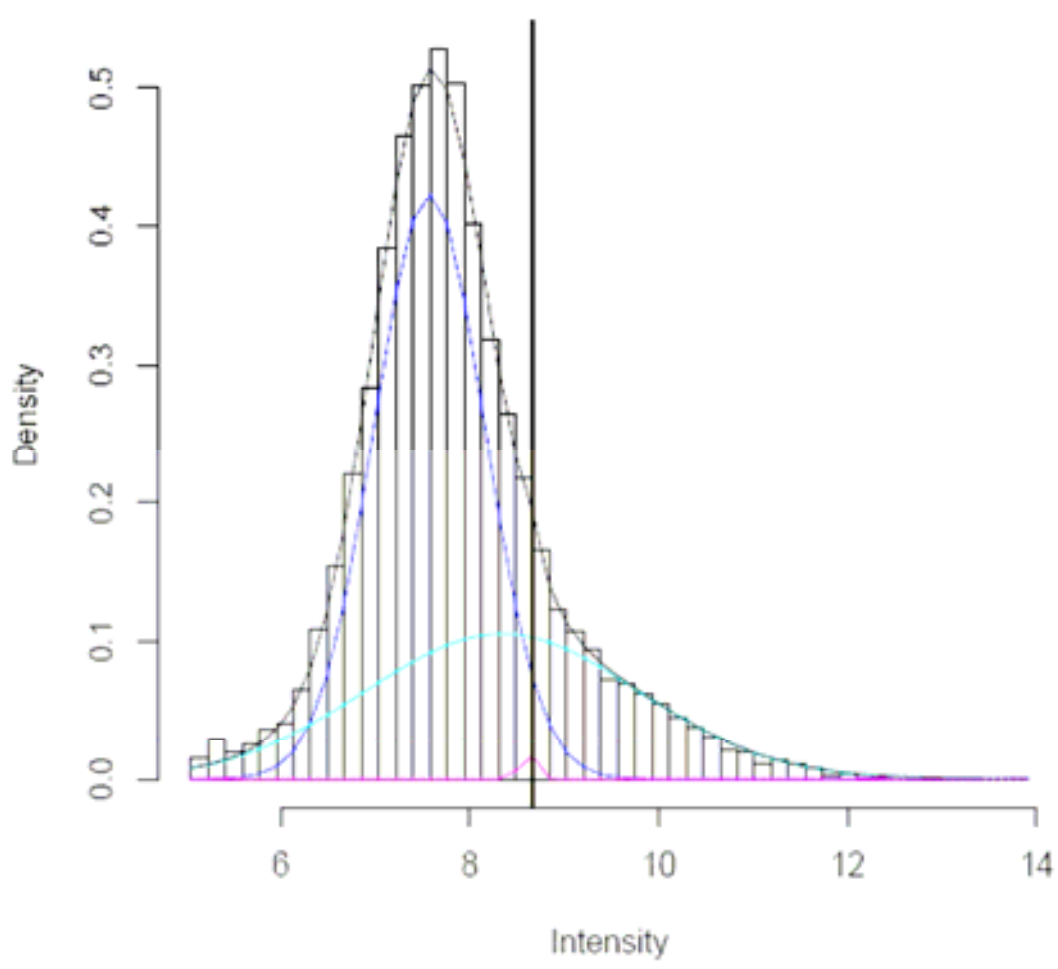

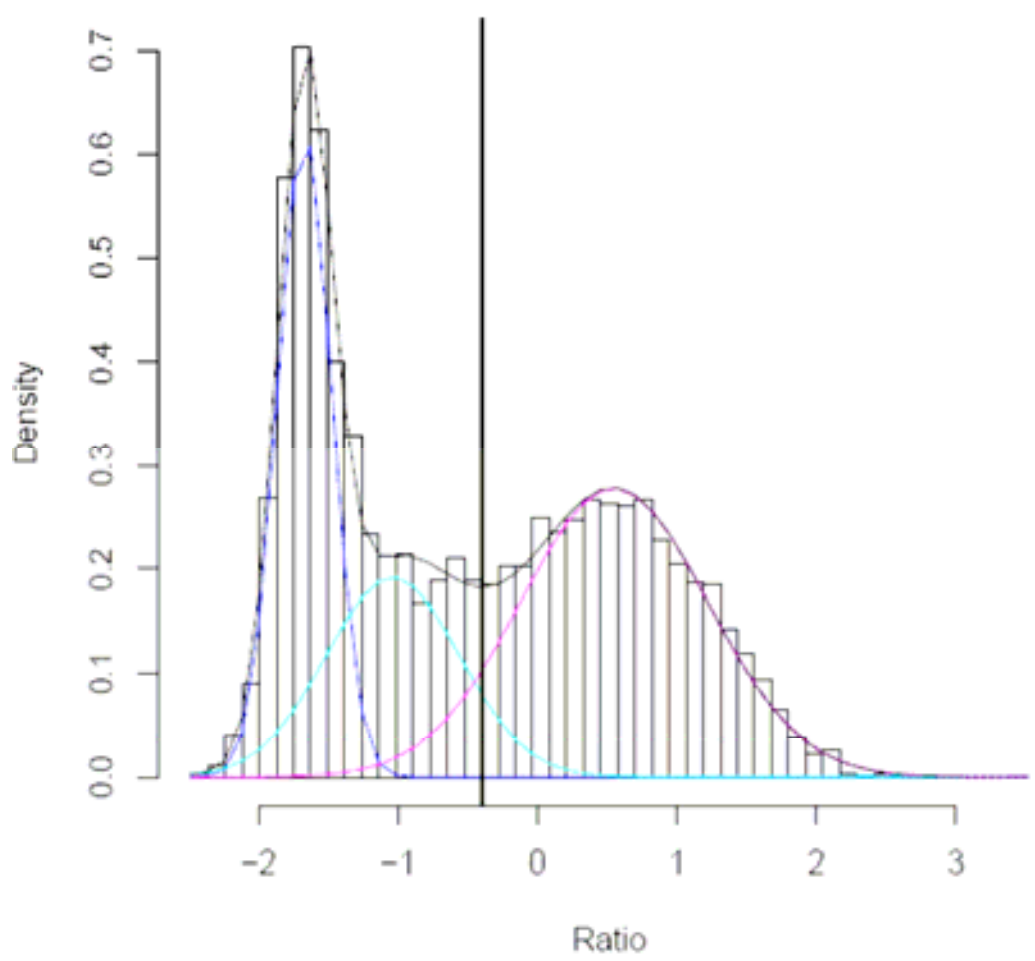

H3K27me3 (*lhp1*)

Hybridization threshold = 7.3909

Ratio threshold = - 1.313639

Replicate1

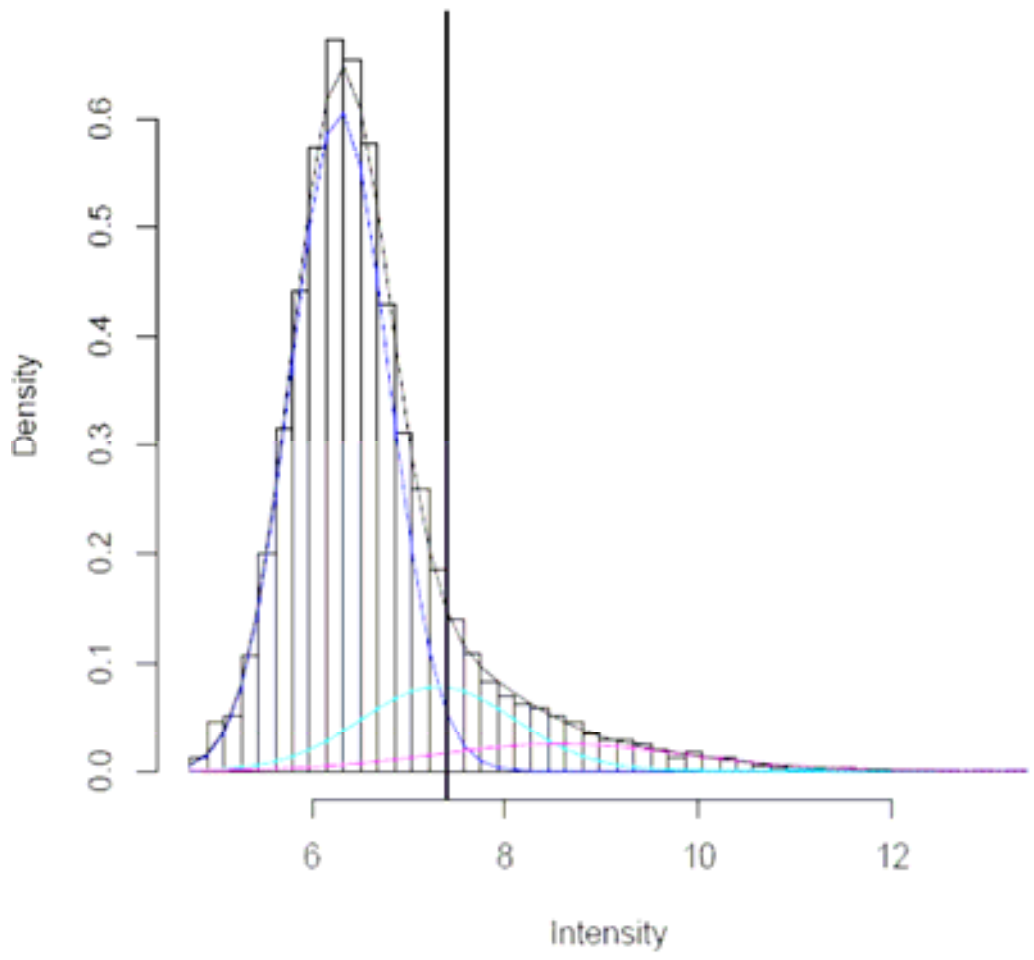

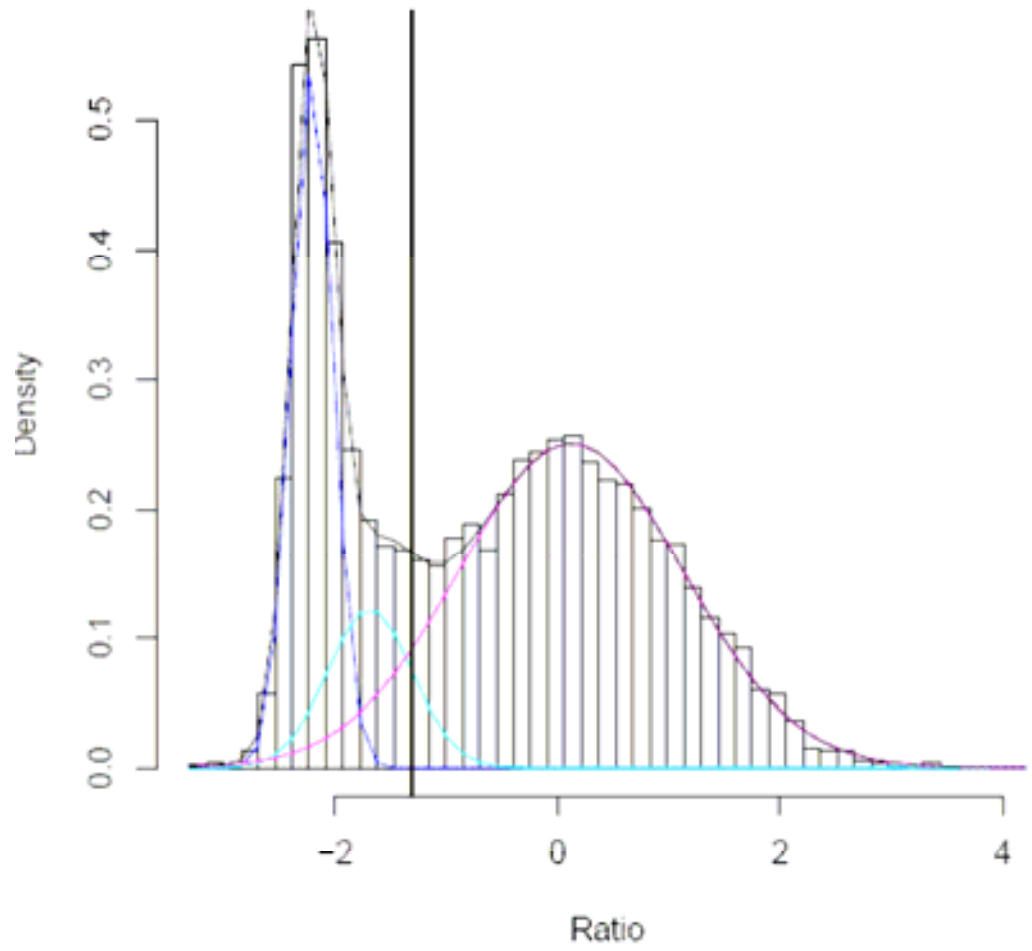

H3K9me2 (WT)

Replicate1

Hybridization threshold = 9.3852

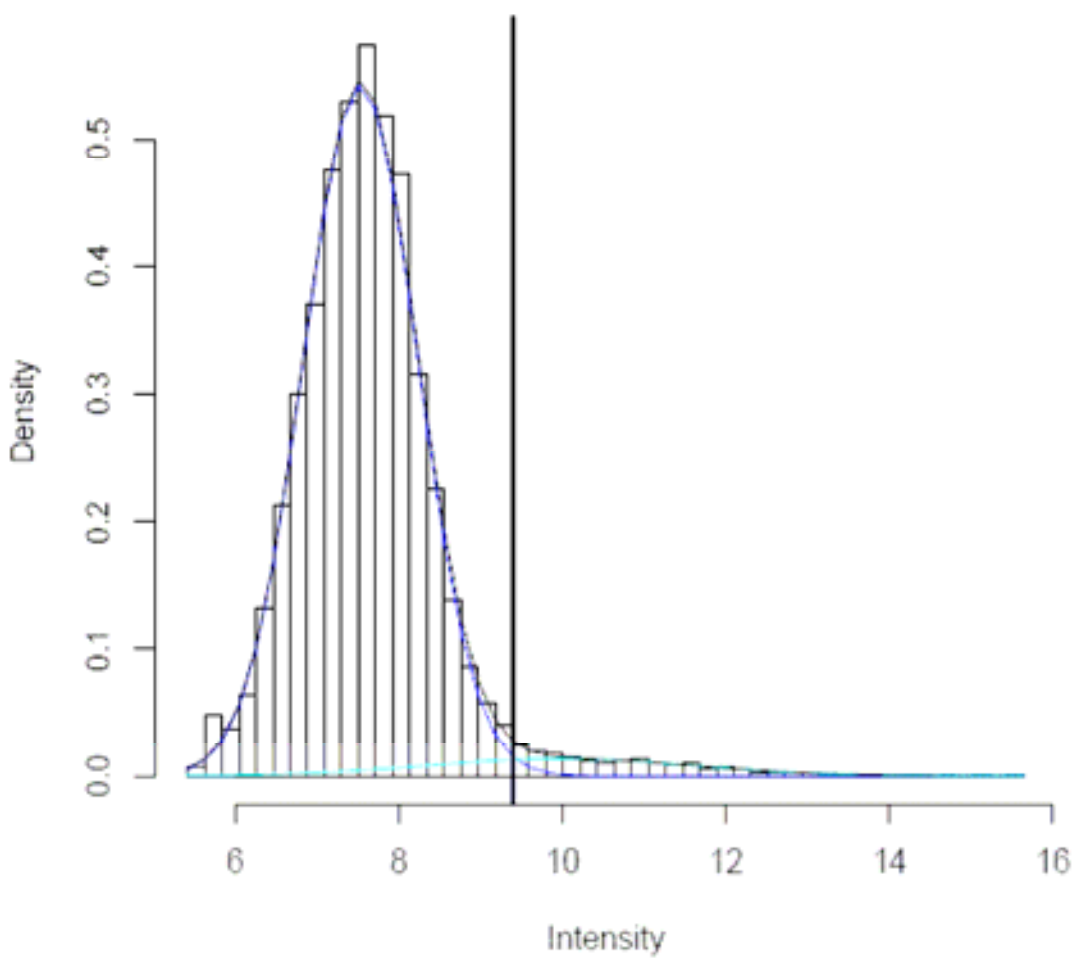

Ratio threshold = = 0.7232

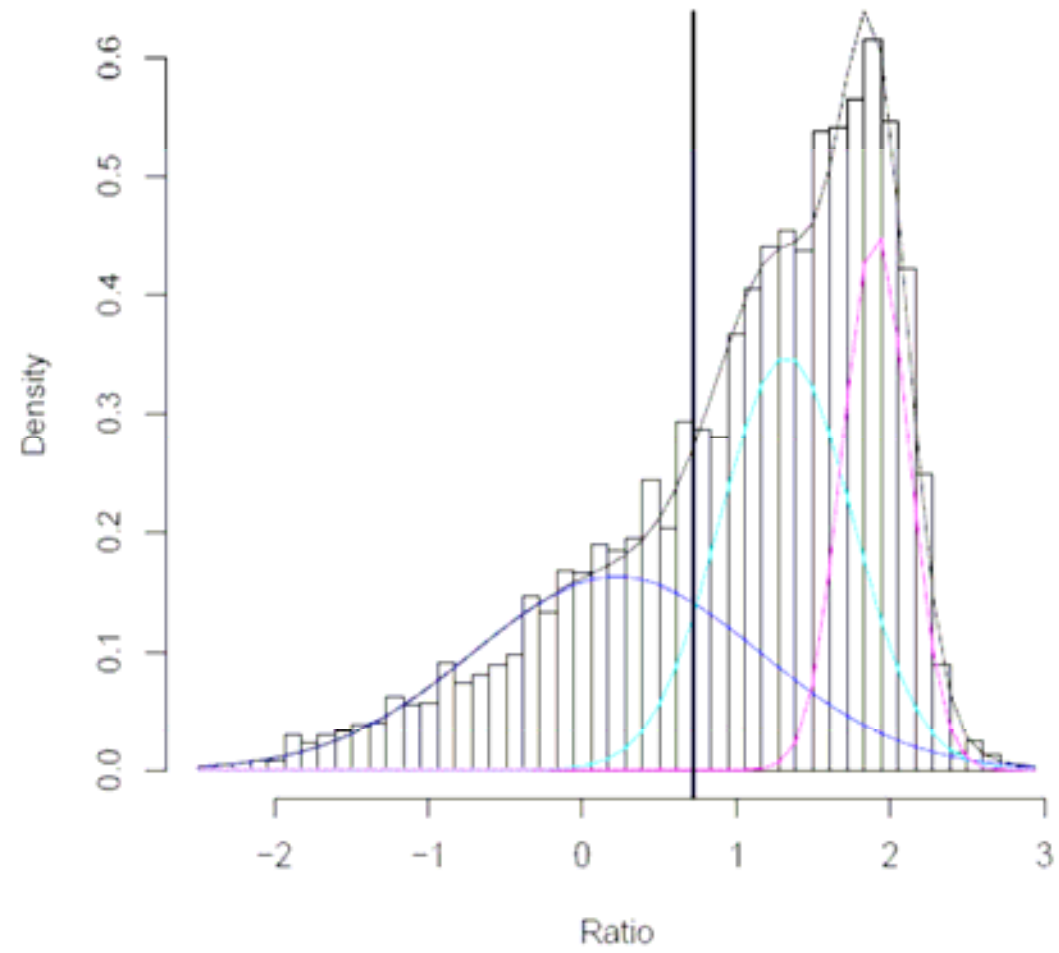

Replicate2

Hybridization threshold = 9.0944

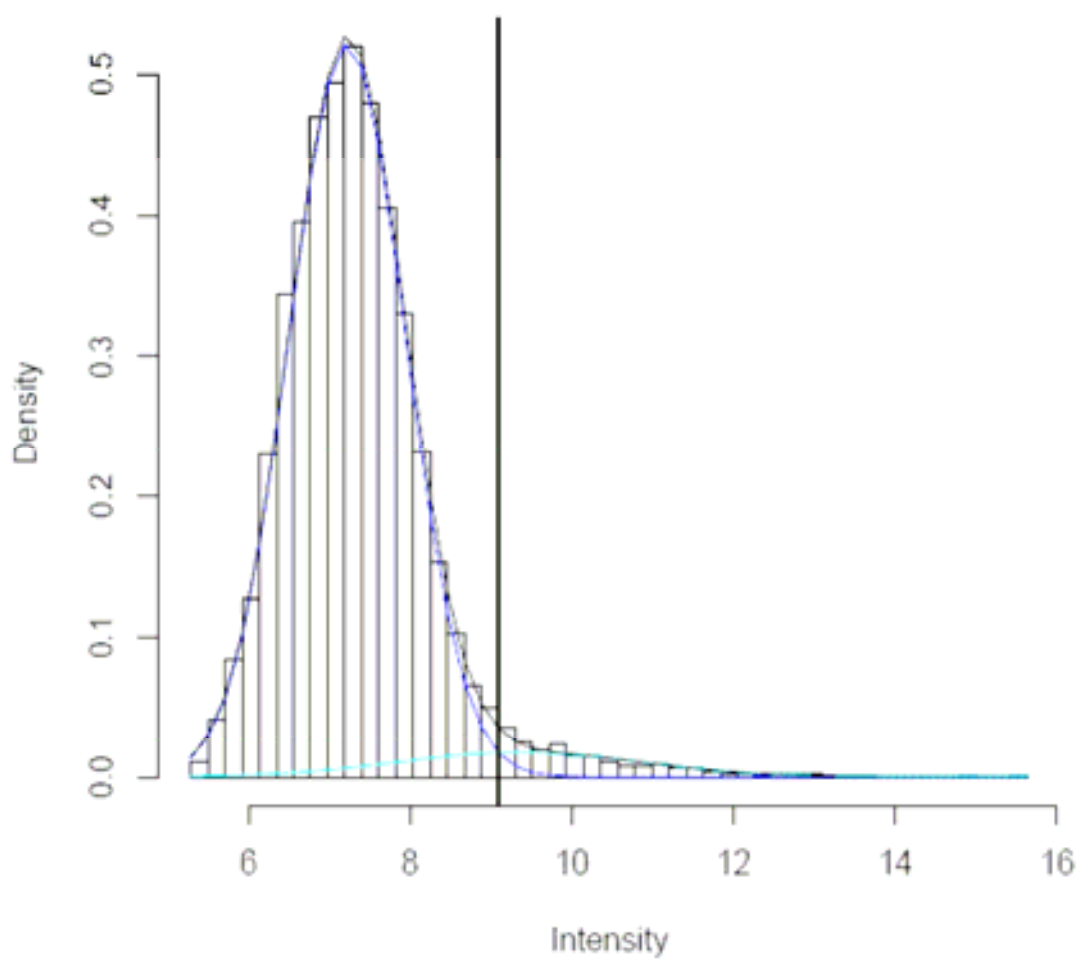

Ratio threshold = 0.7176

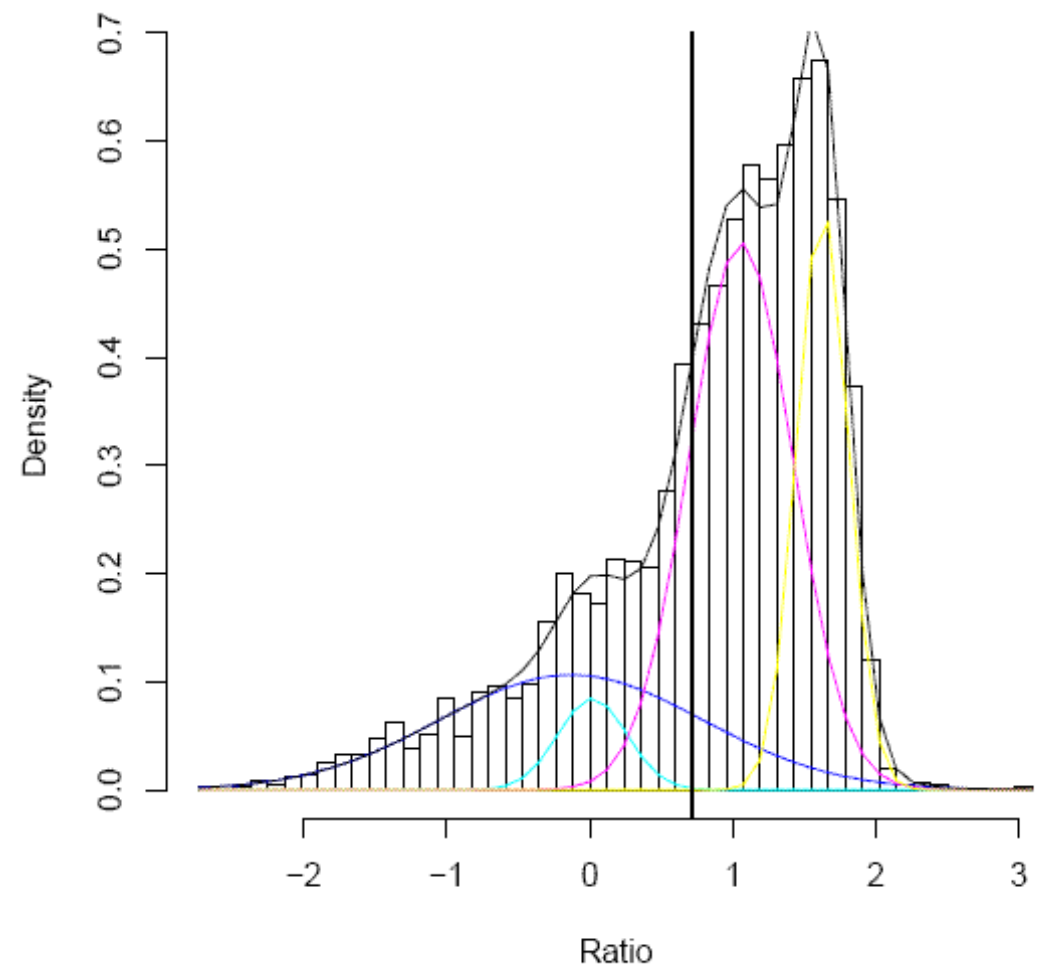

H3K9me3 (WT)

Hybridization threshold = 7.37253

Ratio threshold = = - 1.039546

Replicate1

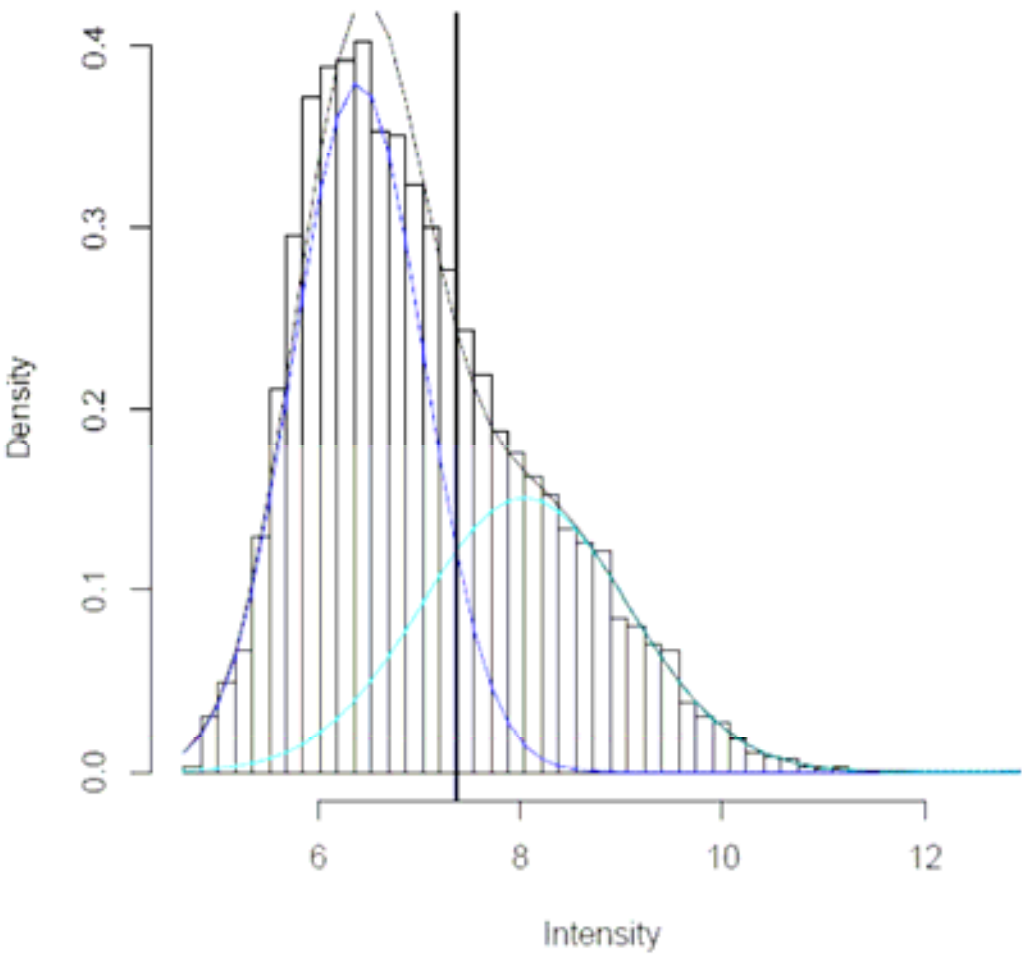

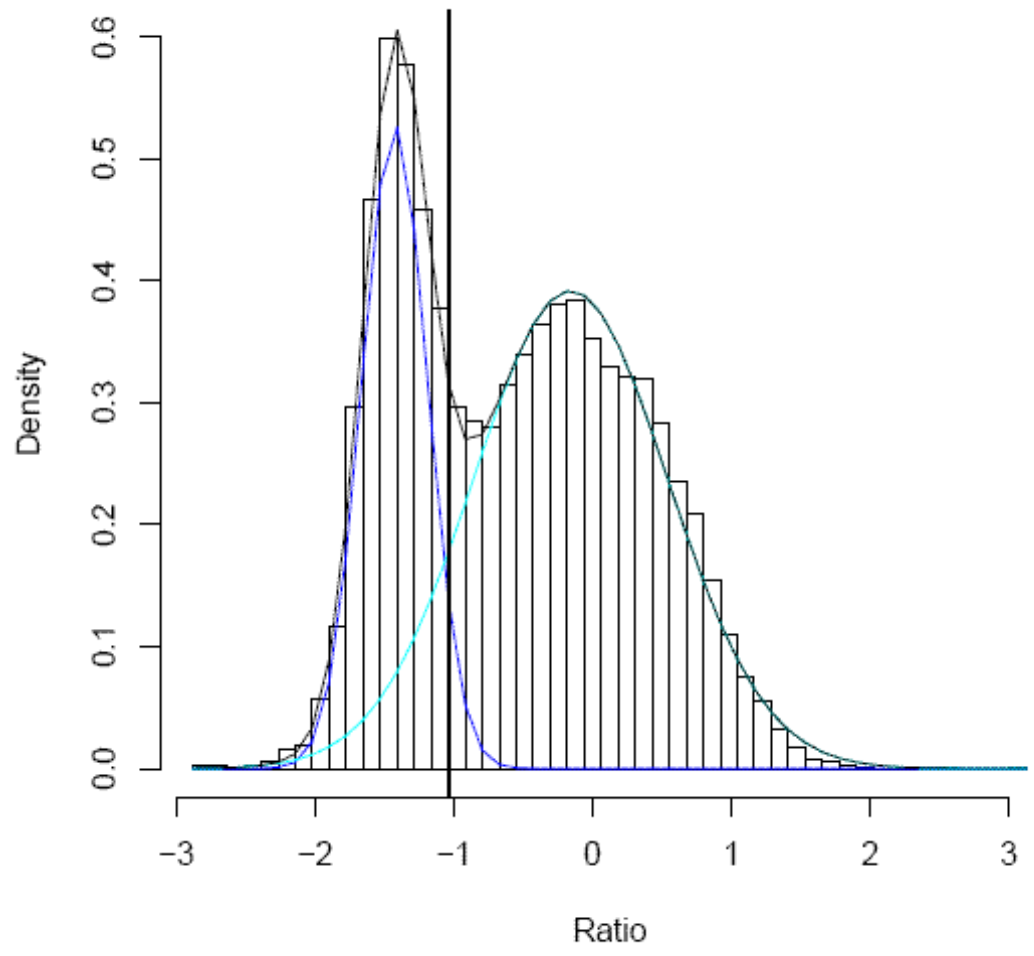

Hybridization threshold = 6.809251

Ratio threshold = - 1.420660

Replicate2

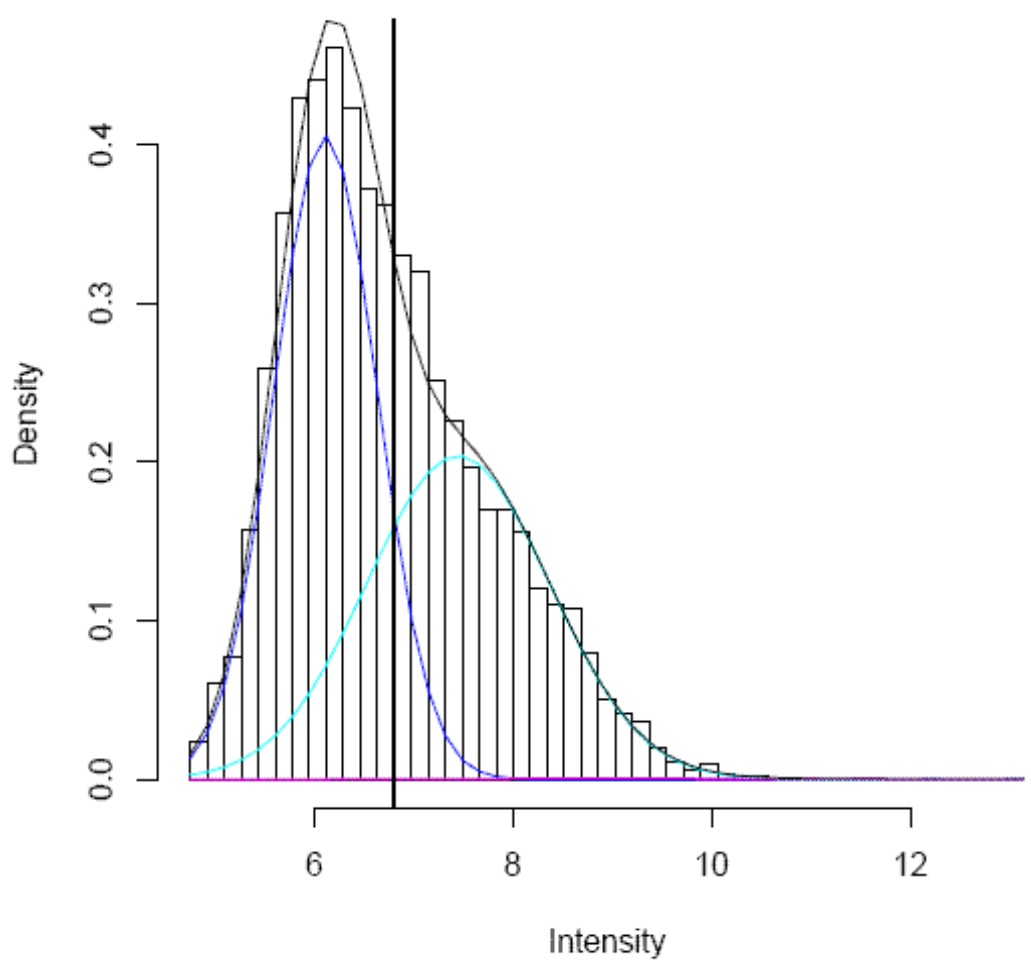

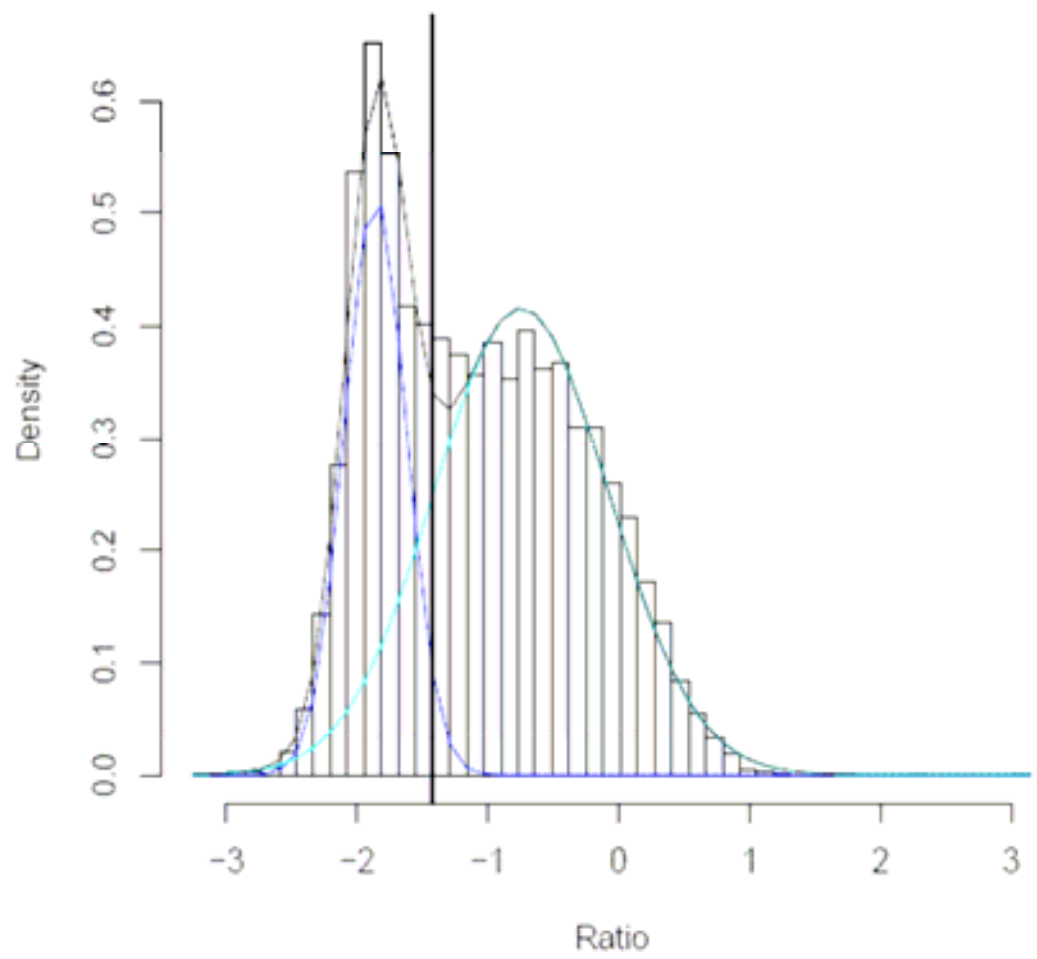

Supplement: Supplementary file 1 [file pgen.0030086.sg001.pdf]
