# Supplementary material for: Arabidopsis TFL2/LHP1 Specifically Associates with Genes Marked by Trimethylation of Histone H3 Lysine 27
Source: PLoS Genet. 2007 Jun 1;3(6):e86. doi: 10.1371/journal.pgen.0030086 (PMC1885283; doi:10.1371/journal.pgen.0030086)
Supplement: Supplementary file 2 [file pgen.0030086.sg002.pdf]

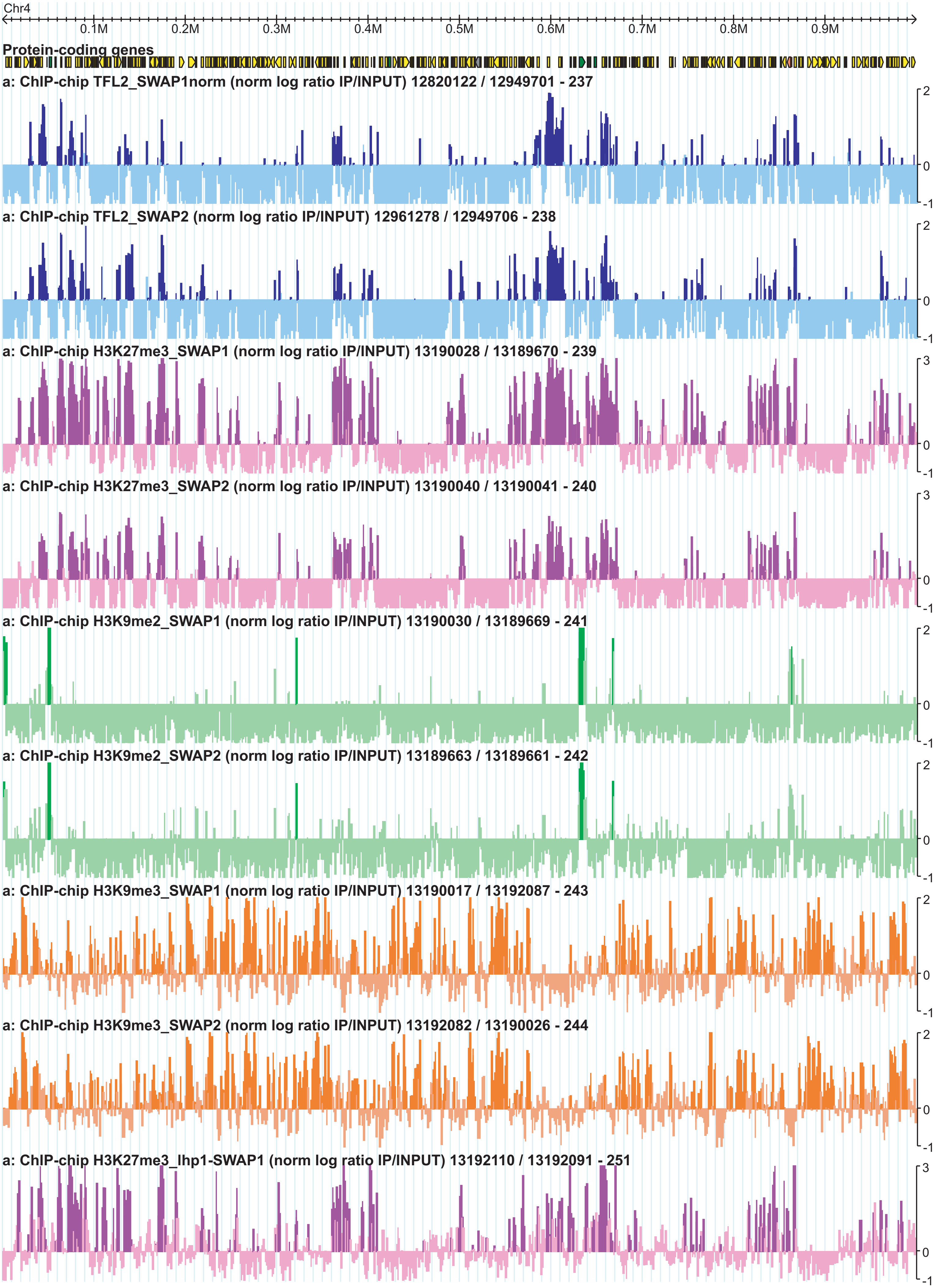
Chr4
0.1M
0.2M
0.3M
0.4M
0.5M
0.6M
0.7M
0.8M
0.9M
Protein-coding genes
a: ChIP-chip TFL2_SWAP1norm (norm log ratio IP/INPUT) 12820122 / 12949701 - 237
2
0
-1
a: ChIP-chip TFL2_SWAP2 (norm log ratio IP/INPUT) 12961278 / 12949706 - 238
2
0
-1
a: ChIP-chip H3K27me3_SWAP1 (norm log ratio IP/INPUT) 13190028 / 13189670 - 239
3
0
-1
a: ChIP-chip H3K27me3_SWAP2 (norm log ratio IP/INPUT) 13190040 / 13190041 - 240
3
0
-1
a: ChIP-chip H3K9me2_SWAP1 (norm log ratio IP/INPUT) 13190030 / 13189669 - 241
2
0
-1
a: ChIP-chip H3K9me2_SWAP2 (norm log ratio IP/INPUT) 13189663 / 13189661 - 242
2
0
-1
a: ChIP-chip H3K9me3_SWAP1 (norm log ratio IP/INPUT) 13190017 / 13192087 - 243
2
0
-1
a: ChIP-chip H3K9me3_SWAP2 (norm log ratio IP/INPUT) 13192082 / 13190026 - 244
2
0
-1
a: ChIP-chip H3K27me3_lhp1-SWAP1 (norm log ratio IP/INPUT) 13192110 / 13192091 - 251
3
0
-1

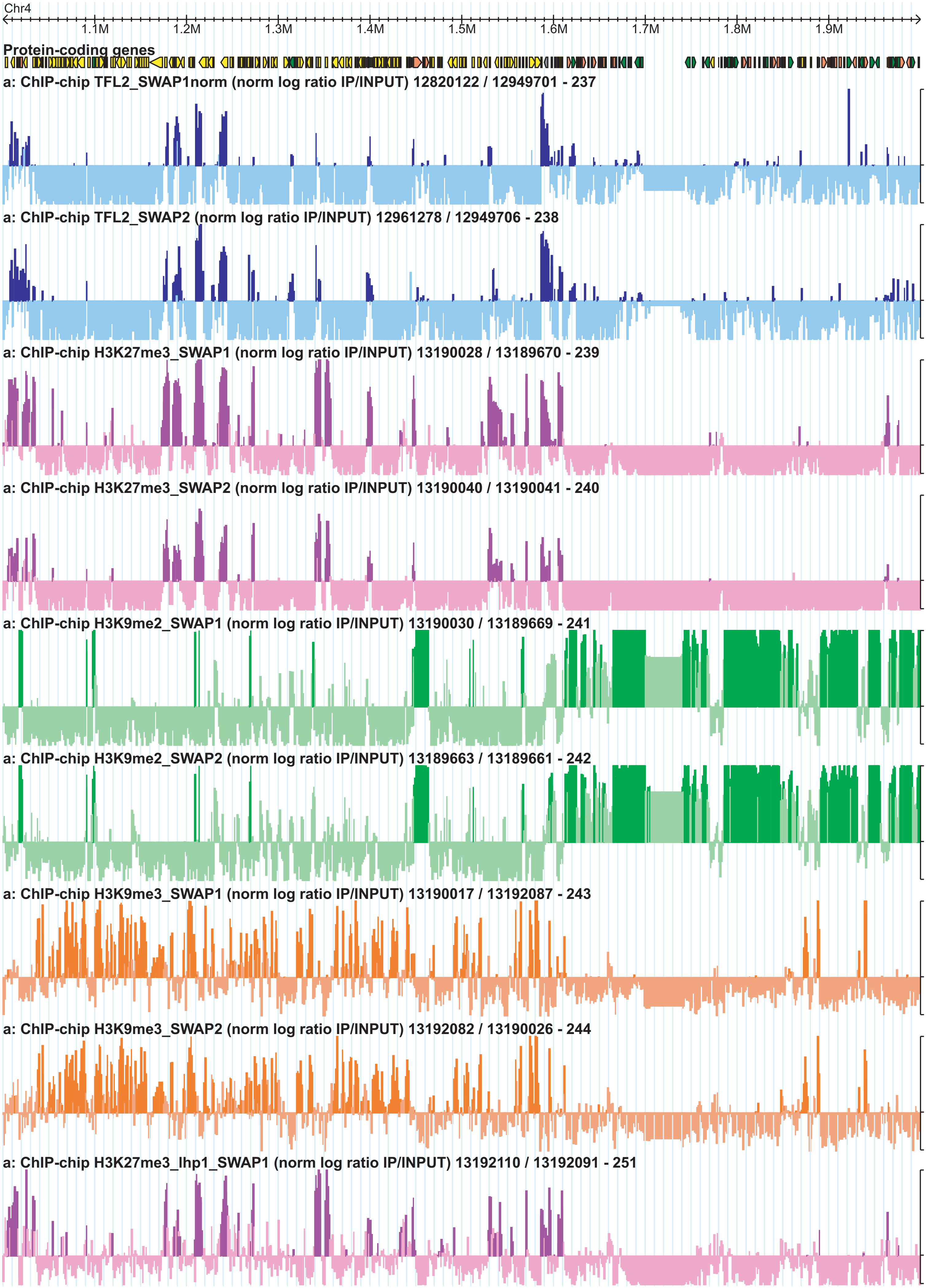

Chr4
1.1M
1.2M
1.3M
1.4M
1.5M
1.6M
1.7M
1.8M
1.9M
Protein-coding genes
a: ChIP-chip TFL2_SWAP1norm (norm log ratio IP/INPUT) 12820122 / 12949701 - 237
a: ChIP-chip TFL2_SWAP2 (norm log ratio IP/INPUT) 12961278 / 12949706 - 238
a: ChIP-chip H3K27me3_SWAP1 (norm log ratio IP/INPUT) 13190028 / 13189670 - 239
a: ChIP-chip H3K27me3_SWAP2 (norm log ratio IP/INPUT) 13190040 / 13190041 - 240
a: ChIP-chip H3K9me2_SWAP1 (norm log ratio IP/INPUT) 13190030 / 13189669 - 241
a: ChIP-chip H3K9me2_SWAP2 (norm log ratio IP/INPUT) 13189663 / 13189661 - 242
a: ChIP-chip H3K9me3_SWAP1 (norm log ratio IP/INPUT) 13190017 / 13192087 - 243
a: ChIP-chip H3K9me3_SWAP2 (norm log ratio IP/INPUT) 13192082 / 13190026 - 244
a: ChIP-chip H3K27me3_lhp1_SWAP1 (norm log ratio IP/INPUT) 13192110 / 13192091 - 251

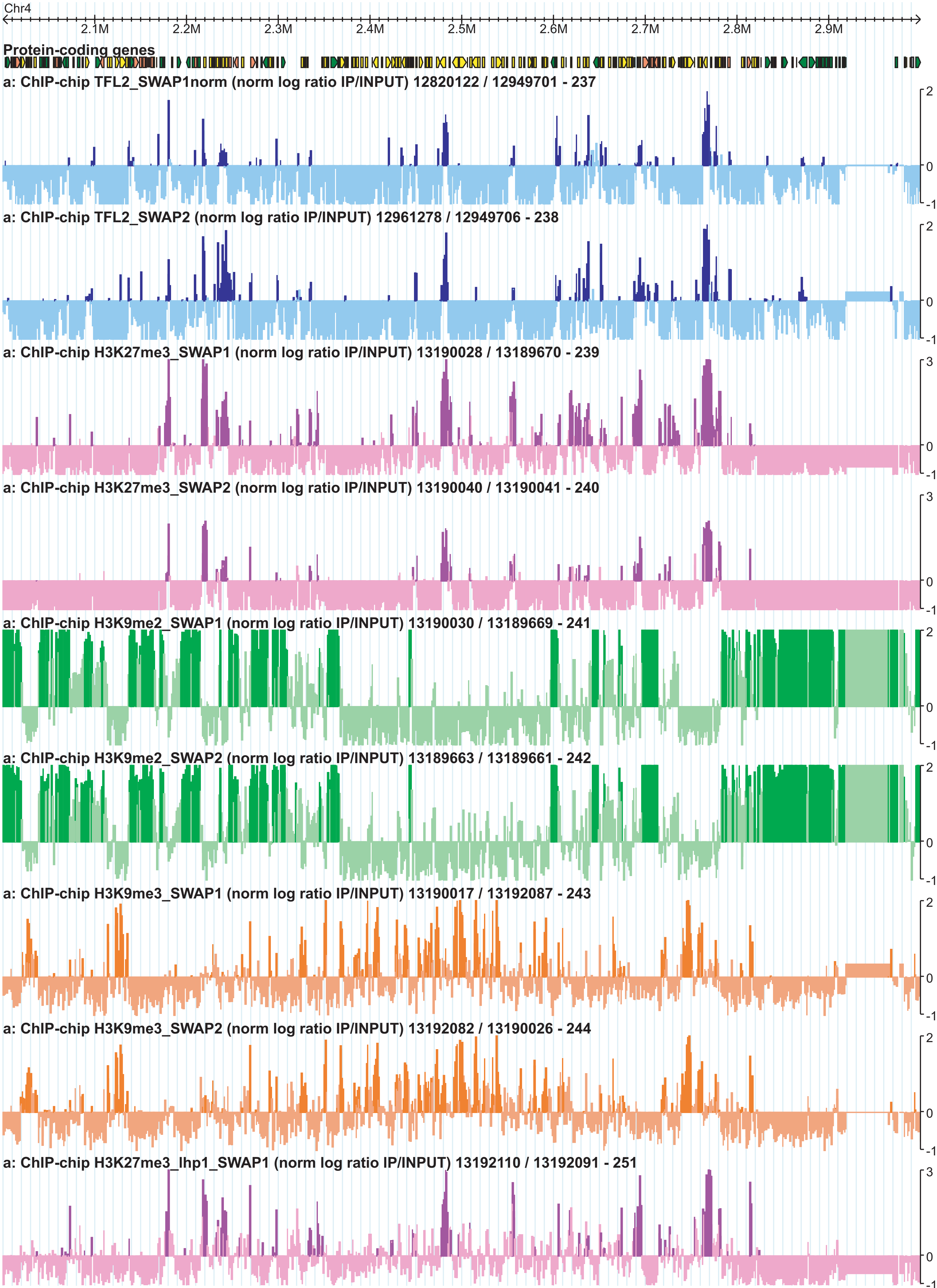
Chr4
2.1M
2.2M
2.3M
2.4M
2.5M
2.6M
2.7M
2.8M
2.9M
Protein-coding genes
a: ChIP-chip TFL2_SWAP1norm (norm log ratio IP/INPUT) 12820122 / 12949701 - 237
a: ChIP-chip TFL2_SWAP2 (norm log ratio IP/INPUT) 12961278 / 12949706 - 238
a: ChIP-chip H3K27me3_SWAP1 (norm log ratio IP/INPUT) 13190028 / 13189670 - 239
a: ChIP-chip H3K27me3_SWAP2 (norm log ratio IP/INPUT) 13190040 / 13190041 - 240
a: ChIP-chip H3K9me2_SWAP1 (norm log ratio IP/INPUT) 13190030 / 13189669 - 241
a: ChIP-chip H3K9me2_SWAP2 (norm log ratio IP/INPUT) 13189663 / 13189661 - 242
a: ChIP-chip H3K9me3_SWAP1 (norm log ratio IP/INPUT) 13190017 / 13192087 - 243
a: ChIP-chip H3K9me3_SWAP2 (norm log ratio IP/INPUT) 13192082 / 13190026 - 244
a: ChIP-chip H3K27me3_lhp1_SWAP1 (norm log ratio IP/INPUT) 13192110 / 13192091 - 251

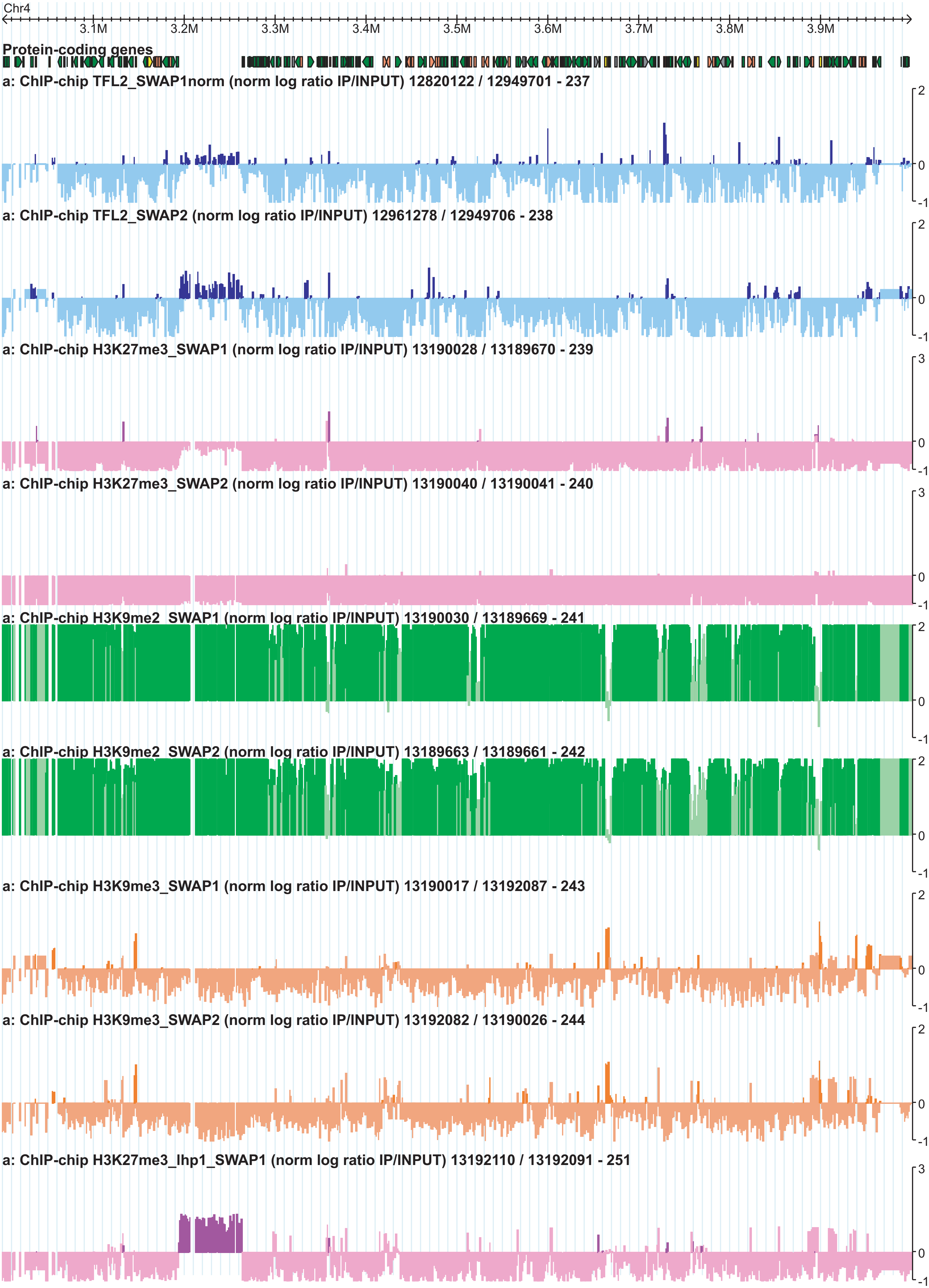

Chr4
3.1M
3.2M
3.3M
3.4M
3.5M
3.6M
3.7M
3.8M
3.9M
Protein-coding genes
a: ChIP-chip TFL2_SWAP1norm (norm log ratio IP/INPUT) 12820122 / 12949701 - 237
a: ChIP-chip TFL2_SWAP2 (norm log ratio IP/INPUT) 12961278 / 12949706 - 238
a: ChIP-chip H3K27me3_SWAP1 (norm log ratio IP/INPUT) 13190028 / 13189670 - 239
a: ChIP-chip H3K27me3_SWAP2 (norm log ratio IP/INPUT) 13190040 / 13190041 - 240
a: ChIP-chip H3K9me2_SWAP1 (norm log ratio IP/INPUT) 13190030 / 13189669 - 241
a: ChIP-chip H3K9me2_SWAP2 (norm log ratio IP/INPUT) 13189663 / 13189661 - 242
a: ChIP-chip H3K9me3_SWAP1 (norm log ratio IP/INPUT) 13190017 / 13192087 - 243
a: ChIP-chip H3K9me3_SWAP2 (norm log ratio IP/INPUT) 13192082 / 13190026 - 244
a: ChIP-chip H3K27me3_lhp1_SWAP1 (norm log ratio IP/INPUT) 13192110 / 13192091 - 251

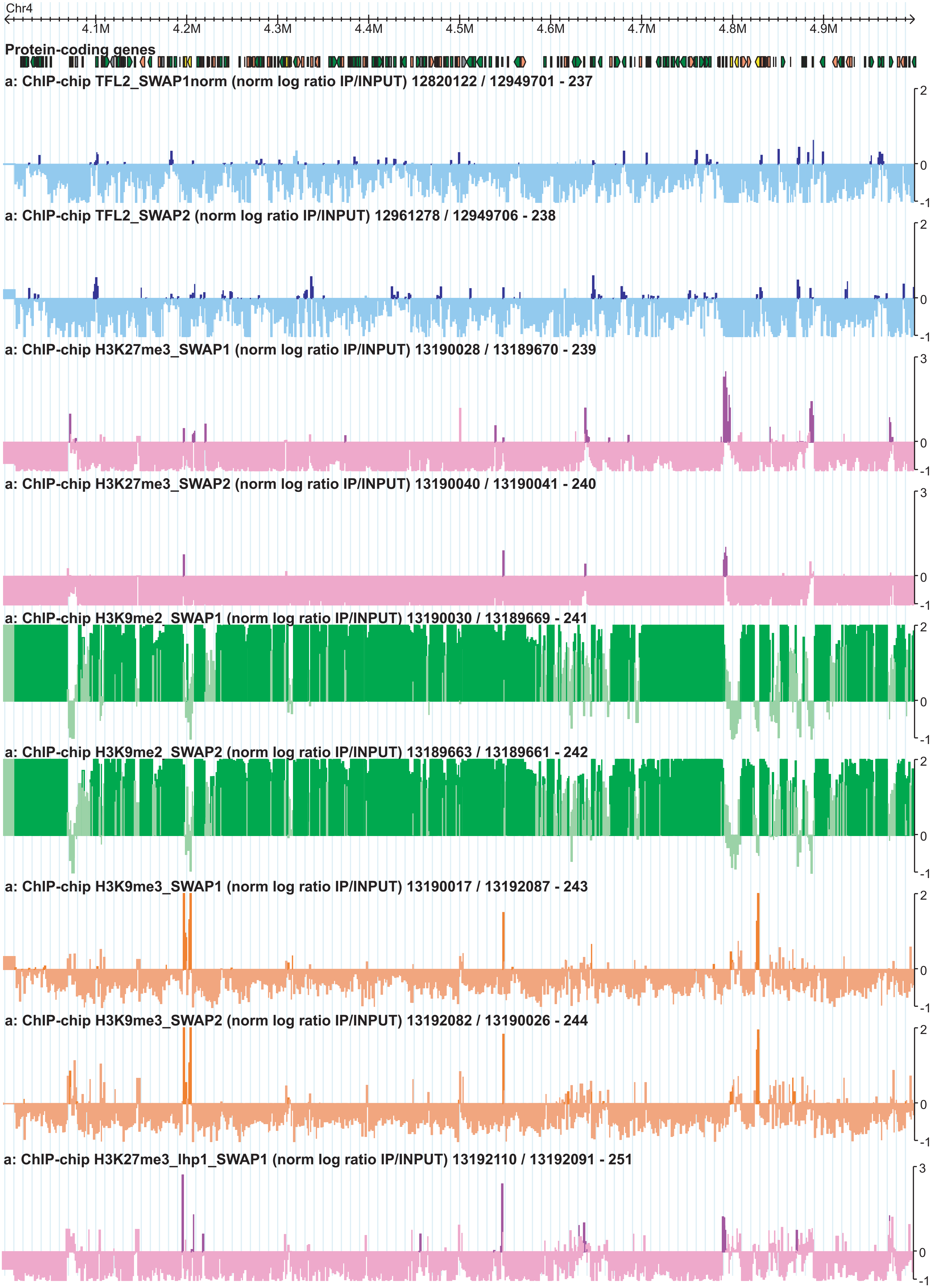

Chr4
4.1M
4.2M
4.3M
4.4M
4.5M
4.6M
4.7M
4.8M
4.9M
Protein-coding genes
a: ChIP-chip TFL2_SWAP1norm (norm log ratio IP/INPUT) 12820122 / 12949701 - 237
2
0
-1
a: ChIP-chip TFL2_SWAP2 (norm log ratio IP/INPUT) 12961278 / 12949706 - 238
2
0
-1
a: ChIP-chip H3K27me3_SWAP1 (norm log ratio IP/INPUT) 13190028 / 13189670 - 239
3
0
-1
a: ChIP-chip H3K27me3_SWAP2 (norm log ratio IP/INPUT) 13190040 / 13190041 - 240
3
0
-1
a: ChIP-chip H3K9me2_SWAP1 (norm log ratio IP/INPUT) 13190030 / 13189669 - 241
2
0
-1
a: ChIP-chip H3K9me2_SWAP2 (norm log ratio IP/INPUT) 13189663 / 13189661 - 242
2
0
-1
a: ChIP-chip H3K9me3_SWAP1 (norm log ratio IP/INPUT) 13190017 / 13192087 - 243
2
0
-1
a: ChIP-chip H3K9me3_SWAP2 (norm log ratio IP/INPUT) 13192082 / 13190026 - 244
2
0
-1
a: ChIP-chip H3K27me3_lhp1_SWAP1 (norm log ratio IP/INPUT) 13192110 / 13192091 - 251
3
0
-1

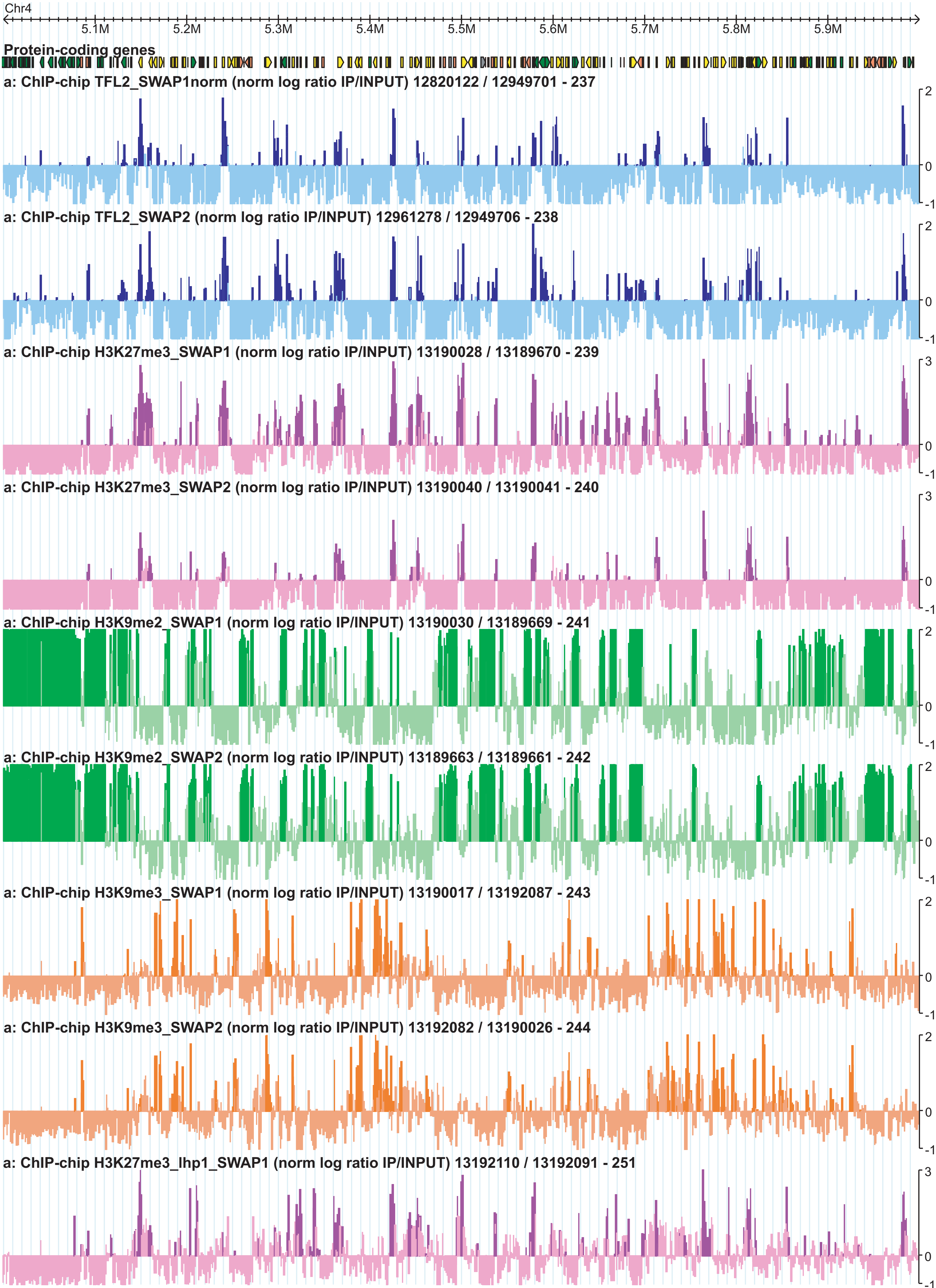

Chr4
5.1M
5.2M
5.3M
5.4M
5.5M
5.6M
5.7M
5.8M
5.9M
Protein-coding genes
a: ChIP-chip TFL2_SWAP1norm (norm log ratio IP/INPUT) 12820122 / 12949701 - 237
a: ChIP-chip TFL2_SWAP2 (norm log ratio IP/INPUT) 12961278 / 12949706 - 238
a: ChIP-chip H3K27me3_SWAP1 (norm log ratio IP/INPUT) 13190028 / 13189670 - 239
a: ChIP-chip H3K27me3_SWAP2 (norm log ratio IP/INPUT) 13190040 / 13190041 - 240
a: ChIP-chip H3K9me2_SWAP1 (norm log ratio IP/INPUT) 13190030 / 13189669 - 241
a: ChIP-chip H3K9me2_SWAP2 (norm log ratio IP/INPUT) 13189663 / 13189661 - 242
a: ChIP-chip H3K9me3_SWAP1 (norm log ratio IP/INPUT) 13190017 / 13192087 - 243
a: ChIP-chip H3K9me3_SWAP2 (norm log ratio IP/INPUT) 13192082 / 13190026 - 244
a: ChIP-chip H3K27me3_lhp1_SWAP1 (norm log ratio IP/INPUT) 13192110 / 13192091 - 251

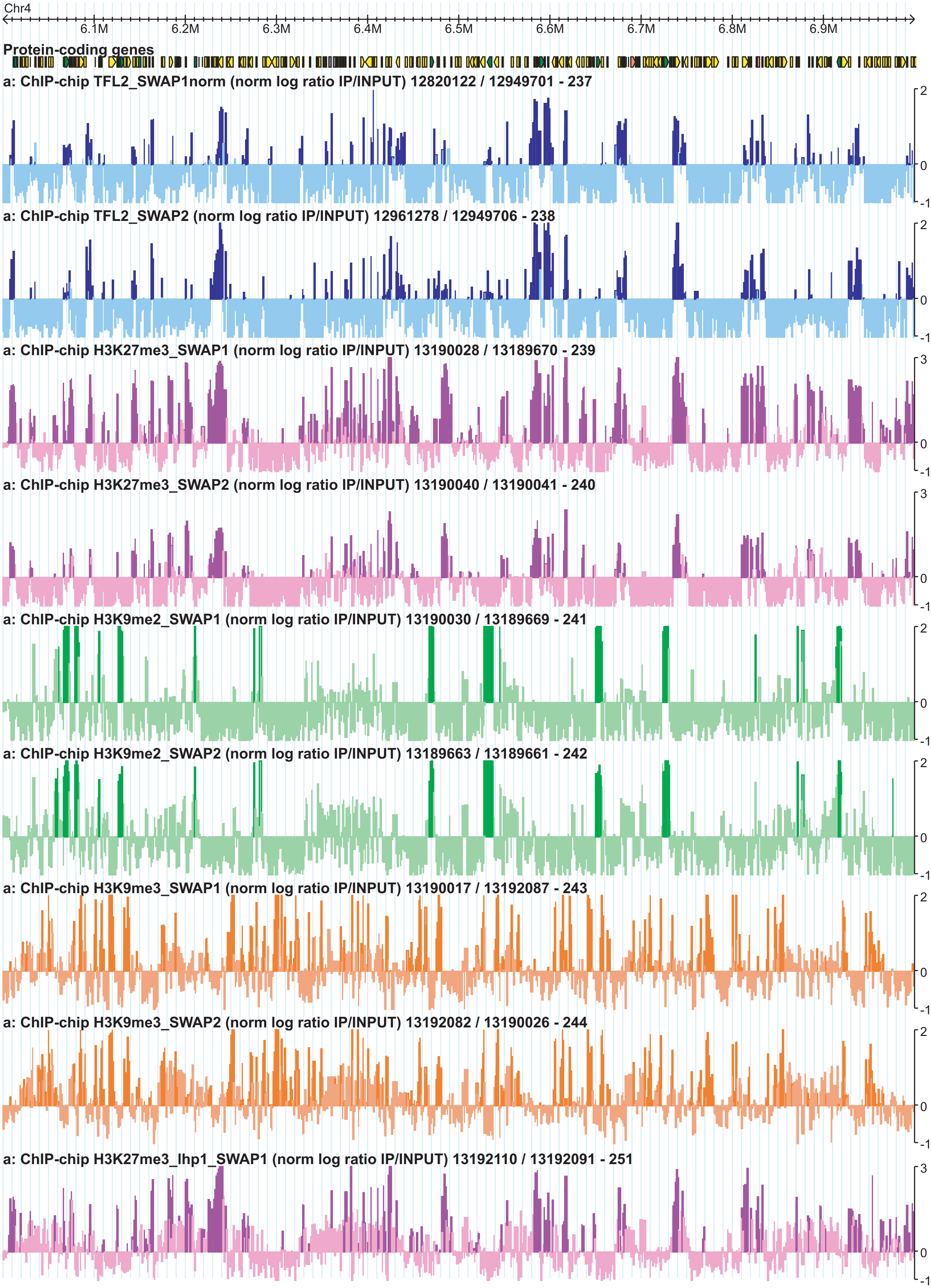
Chr4
6.1M
6.2M
6.3M
6.4M
6.5M
6.6M
6.7M
6.8M
6.9M
Protein-coding genes
a: ChIP-chip TFL2_SWAP1norm (norm log ratio IP/INPUT) 12820122 / 12949701 - 237
a: ChIP-chip TFL2_SWAP2 (norm log ratio IP/INPUT) 12961278 / 12949706 - 238
a: ChIP-chip H3K27me3_SWAP1 (norm log ratio IP/INPUT) 13190028 / 13189670 - 239
a: ChIP-chip H3K27me3_SWAP2 (norm log ratio IP/INPUT) 13190040 / 13190041 - 240
a: ChIP-chip H3K9me2_SWAP1 (norm log ratio IP/INPUT) 13190030 / 13189669 - 241
a: ChIP-chip H3K9me2_SWAP2 (norm log ratio IP/INPUT) 13189663 / 13189661 - 242
a: ChIP-chip H3K9me3_SWAP1 (norm log ratio IP/INPUT) 13190017 / 13192087 - 243
a: ChIP-chip H3K9me3_SWAP2 (norm log ratio IP/INPUT) 13192082 / 13190026 - 244
a: ChIP-chip H3K27me3_lhp1_SWAP1 (norm log ratio IP/INPUT) 13192110 / 13192091 - 251

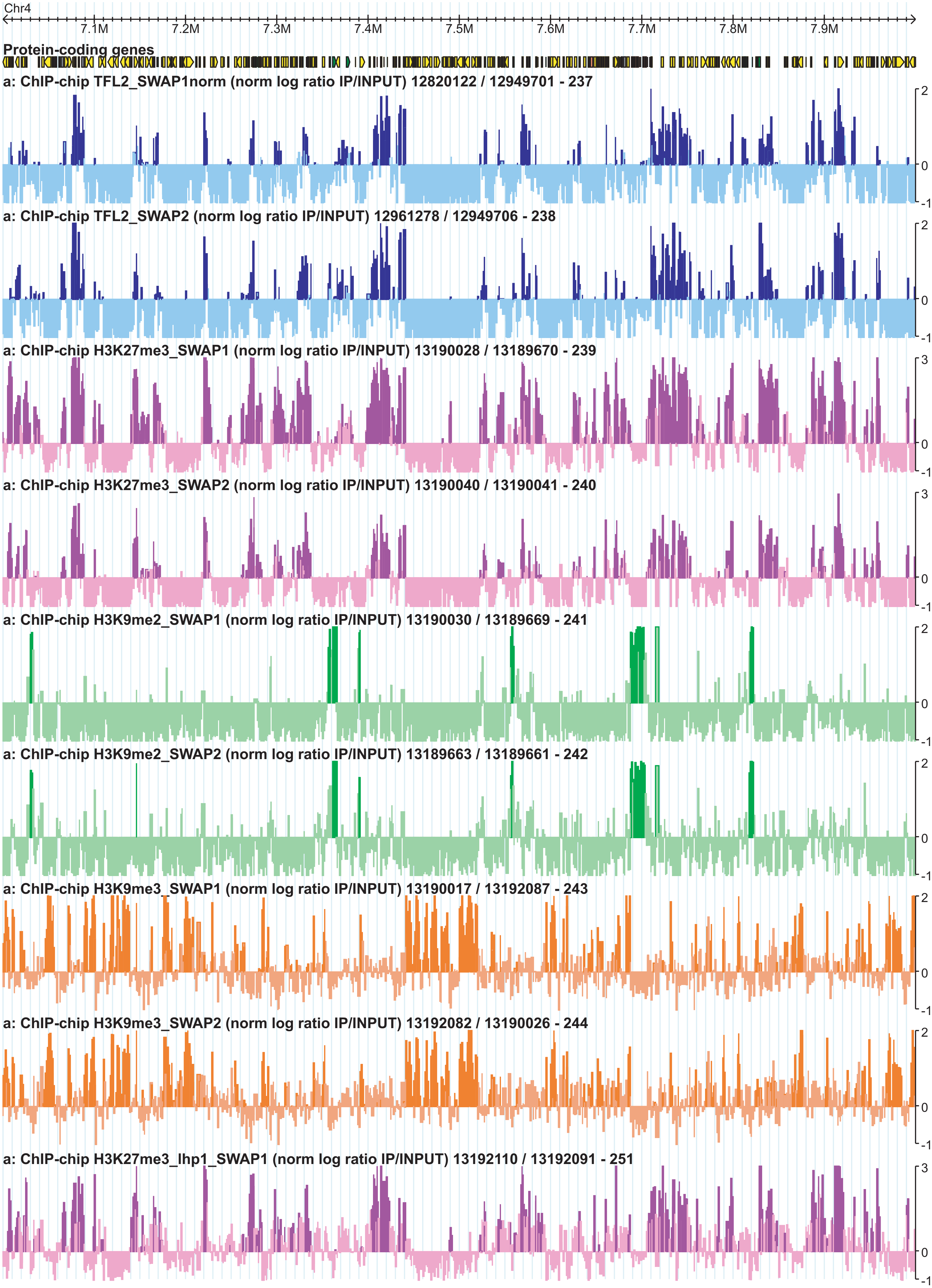
Chr4
7.1M
7.2M
7.3M
7.4M
7.5M
7.6M
7.7M
7.8M
7.9M
Protein-coding genes
a: ChIP-chip TFL2_SWAP1norm (norm log ratio IP/INPUT) 12820122 / 12949701 - 237
a: ChIP-chip TFL2_SWAP2 (norm log ratio IP/INPUT) 12961278 / 12949706 - 238
a: ChIP-chip H3K27me3_SWAP1 (norm log ratio IP/INPUT) 13190028 / 13189670 - 239
a: ChIP-chip H3K27me3_SWAP2 (norm log ratio IP/INPUT) 13190040 / 13190041 - 240
a: ChIP-chip H3K9me2_SWAP1 (norm log ratio IP/INPUT) 13190030 / 13189669 - 241
a: ChIP-chip H3K9me2_SWAP2 (norm log ratio IP/INPUT) 13189663 / 13189661 - 242
a: ChIP-chip H3K9me3_SWAP1 (norm log ratio IP/INPUT) 13190017 / 13192087 - 243
a: ChIP-chip H3K9me3_SWAP2 (norm log ratio IP/INPUT) 13192082 / 13190026 - 244
a: ChIP-chip H3K27me3_lhp1_SWAP1 (norm log ratio IP/INPUT) 13192110 / 13192091 - 251

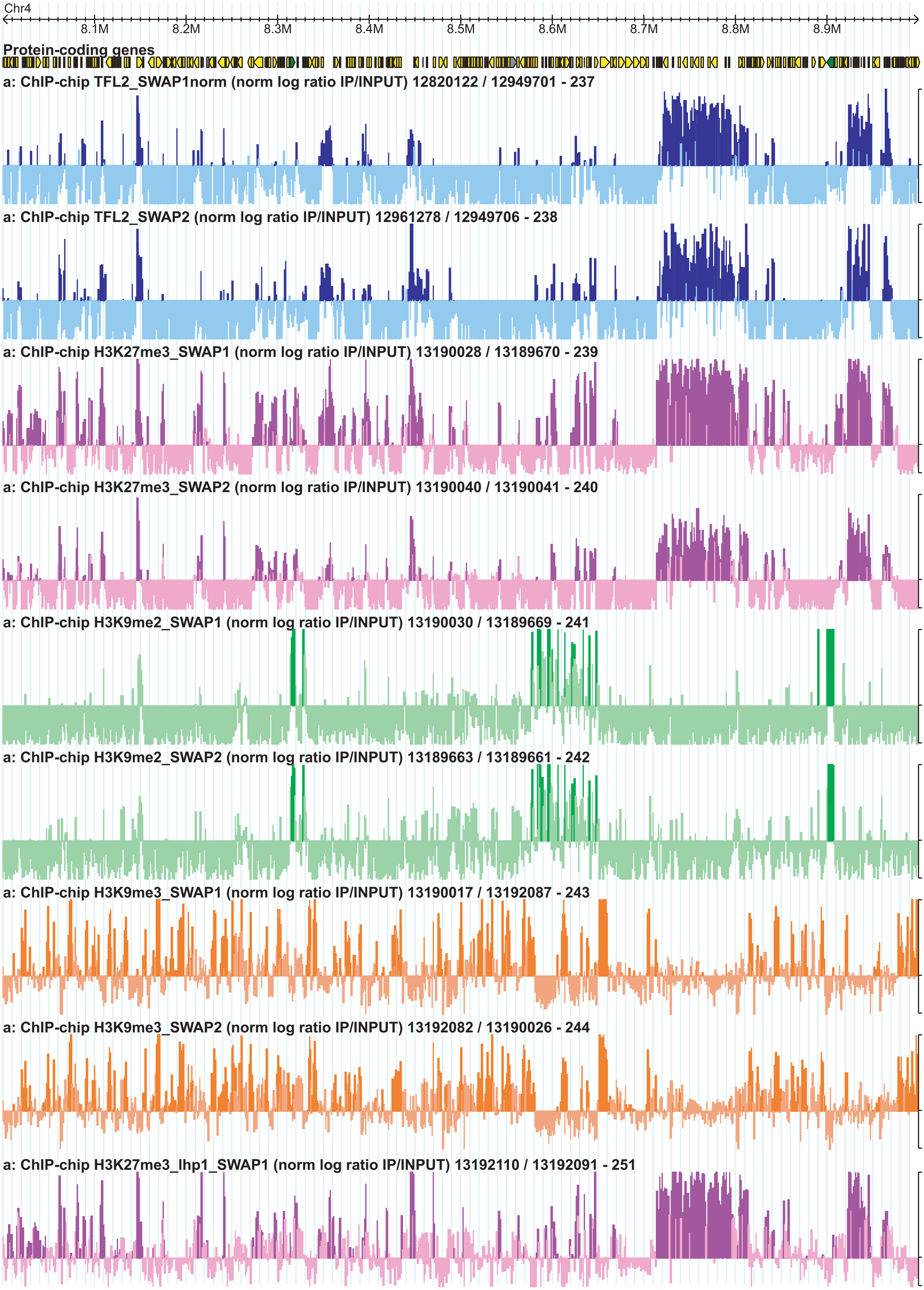
Chr4
8.1M
8.2M
8.3M
8.4M
8.5M
8.6M
8.7M
8.8M
8.9M
Protein-coding genes
a: ChIP-chip TFL2_SWAP1norm (norm log ratio IP/INPUT) 12820122 / 12949701 - 237
2
0
-1
a: ChIP-chip TFL2_SWAP2 (norm log ratio IP/INPUT) 12961278 / 12949706 - 238
2
0
-1
a: ChIP-chip H3K27me3_SWAP1 (norm log ratio IP/INPUT) 13190028 / 13189670 - 239
3
0
-1
a: ChIP-chip H3K27me3_SWAP2 (norm log ratio IP/INPUT) 13190040 / 13190041 - 240
3
0
-1
a: ChIP-chip H3K9me2_SWAP1 (norm log ratio IP/INPUT) 13190030 / 13189669 - 241
2
0
-1
a: ChIP-chip H3K9me2_SWAP2 (norm log ratio IP/INPUT) 13189663 / 13189661 - 242
2
0
-1
a: ChIP-chip H3K9me3_SWAP1 (norm log ratio IP/INPUT) 13190017 / 13192087 - 243
2
0
-1
a: ChIP-chip H3K9me3_SWAP2 (norm log ratio IP/INPUT) 13192082 / 13190026 - 244
2
0
-1
a: ChIP-chip H3K27me3_lhp1_SWAP1 (norm log ratio IP/INPUT) 13192110 / 13192091 - 251
3
0
-1

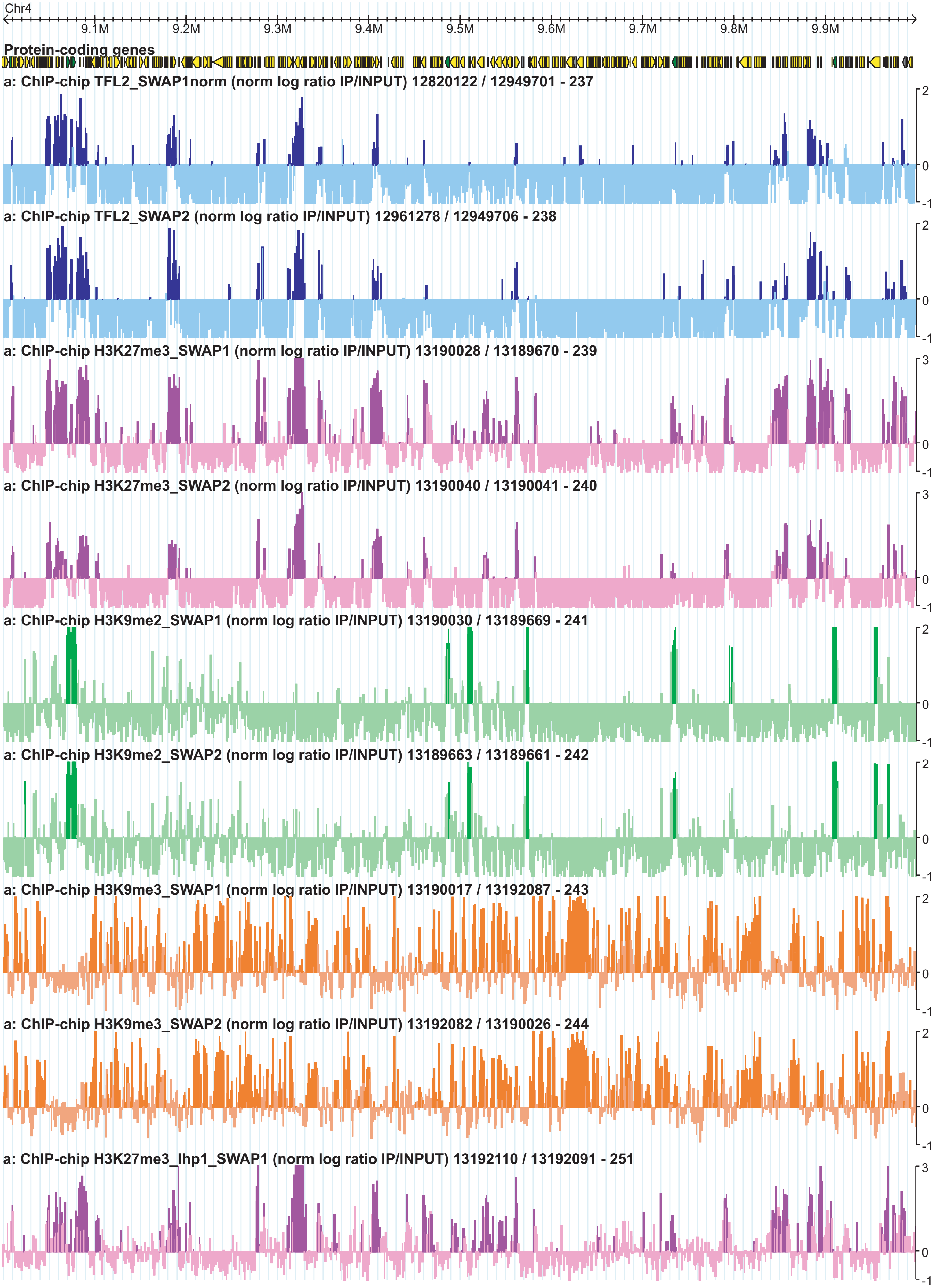
Chr4
9.1M
9.2M
9.3M
9.4M
9.5M
9.6M
9.7M
9.8M
9.9M
Protein-coding genes
a: ChIP-chip TFL2_SWAP1norm (norm log ratio IP/INPUT) 12820122 / 12949701 - 237
a: ChIP-chip TFL2_SWAP2 (norm log ratio IP/INPUT) 12961278 / 12949706 - 238
a: ChIP-chip H3K27me3_SWAP1 (norm log ratio IP/INPUT) 13190028 / 13189670 - 239
a: ChIP-chip H3K27me3_SWAP2 (norm log ratio IP/INPUT) 13190040 / 13190041 - 240
a: ChIP-chip H3K9me2_SWAP1 (norm log ratio IP/INPUT) 13190030 / 13189669 - 241
a: ChIP-chip H3K9me2_SWAP2 (norm log ratio IP/INPUT) 13189663 / 13189661 - 242
a: ChIP-chip H3K9me3_SWAP1 (norm log ratio IP/INPUT) 13190017 / 13192087 - 243
a: ChIP-chip H3K9me3_SWAP2 (norm log ratio IP/INPUT) 13192082 / 13190026 - 244
a: ChIP-chip H3K27me3_lhp1_SWAP1 (norm log ratio IP/INPUT) 13192110 / 13192091 - 251

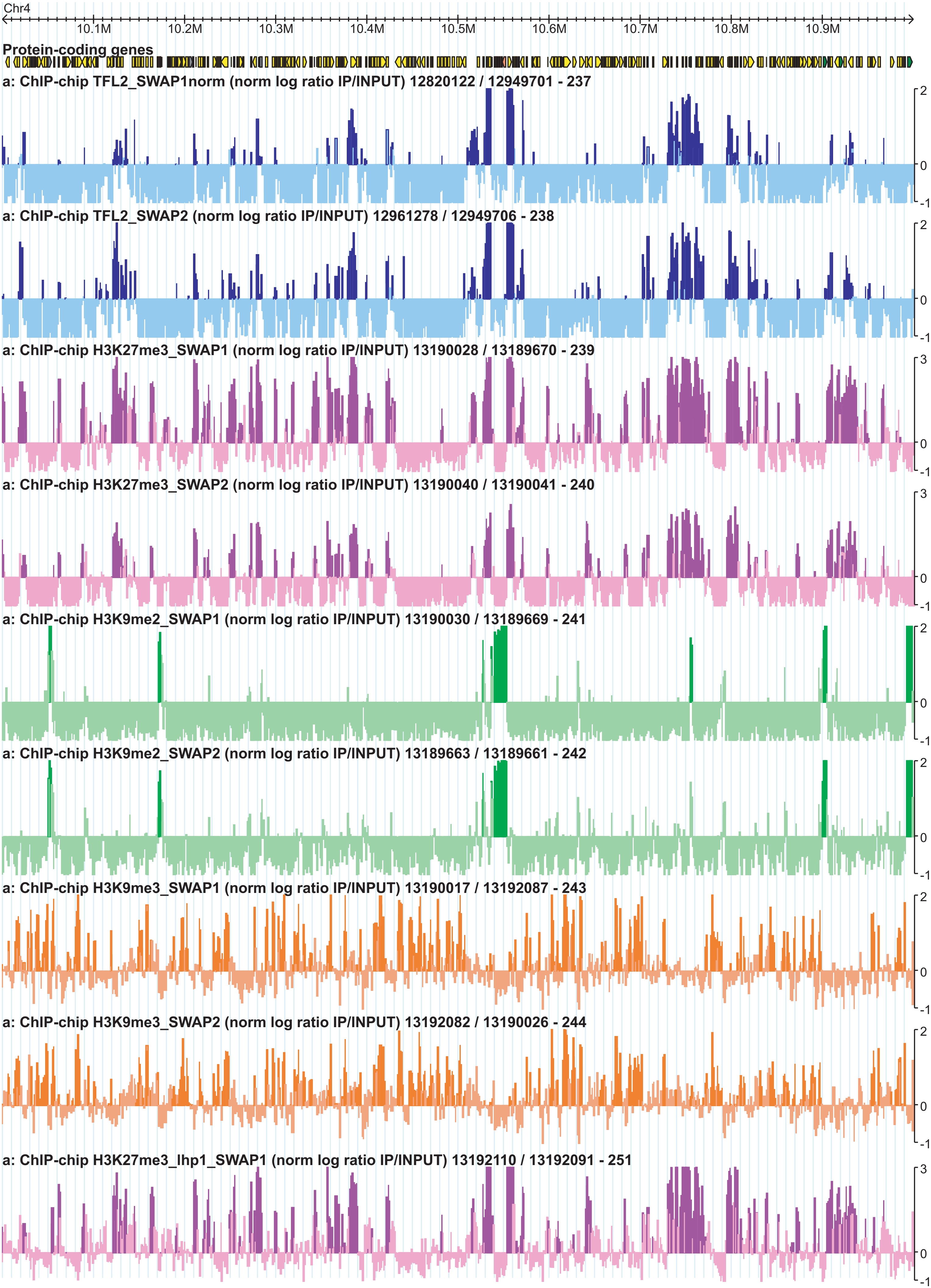

Chr4
10.1M
10.2M
10.3M
10.4M
10.5M
10.6M
10.7M
10.8M
10.9M
Protein-coding genes
a: ChIP-chip TFL2_SWAP1norm (norm log ratio IP/INPUT) 12820122 / 12949701 - 237
a: ChIP-chip TFL2_SWAP2 (norm log ratio IP/INPUT) 12961278 / 12949706 - 238
a: ChIP-chip H3K27me3_SWAP1 (norm log ratio IP/INPUT) 13190028 / 13189670 - 239
a: ChIP-chip H3K27me3_SWAP2 (norm log ratio IP/INPUT) 13190040 / 13190041 - 240
a: ChIP-chip H3K9me2_SWAP1 (norm log ratio IP/INPUT) 13190030 / 13189669 - 241
a: ChIP-chip H3K9me2_SWAP2 (norm log ratio IP/INPUT) 13189663 / 13189661 - 242
a: ChIP-chip H3K9me3_SWAP1 (norm log ratio IP/INPUT) 13190017 / 13192087 - 243
a: ChIP-chip H3K9me3_SWAP2 (norm log ratio IP/INPUT) 13192082 / 13190026 - 244
a: ChIP-chip H3K27me3_lhp1_SWAP1 (norm log ratio IP/INPUT) 13192110 / 13192091 - 251

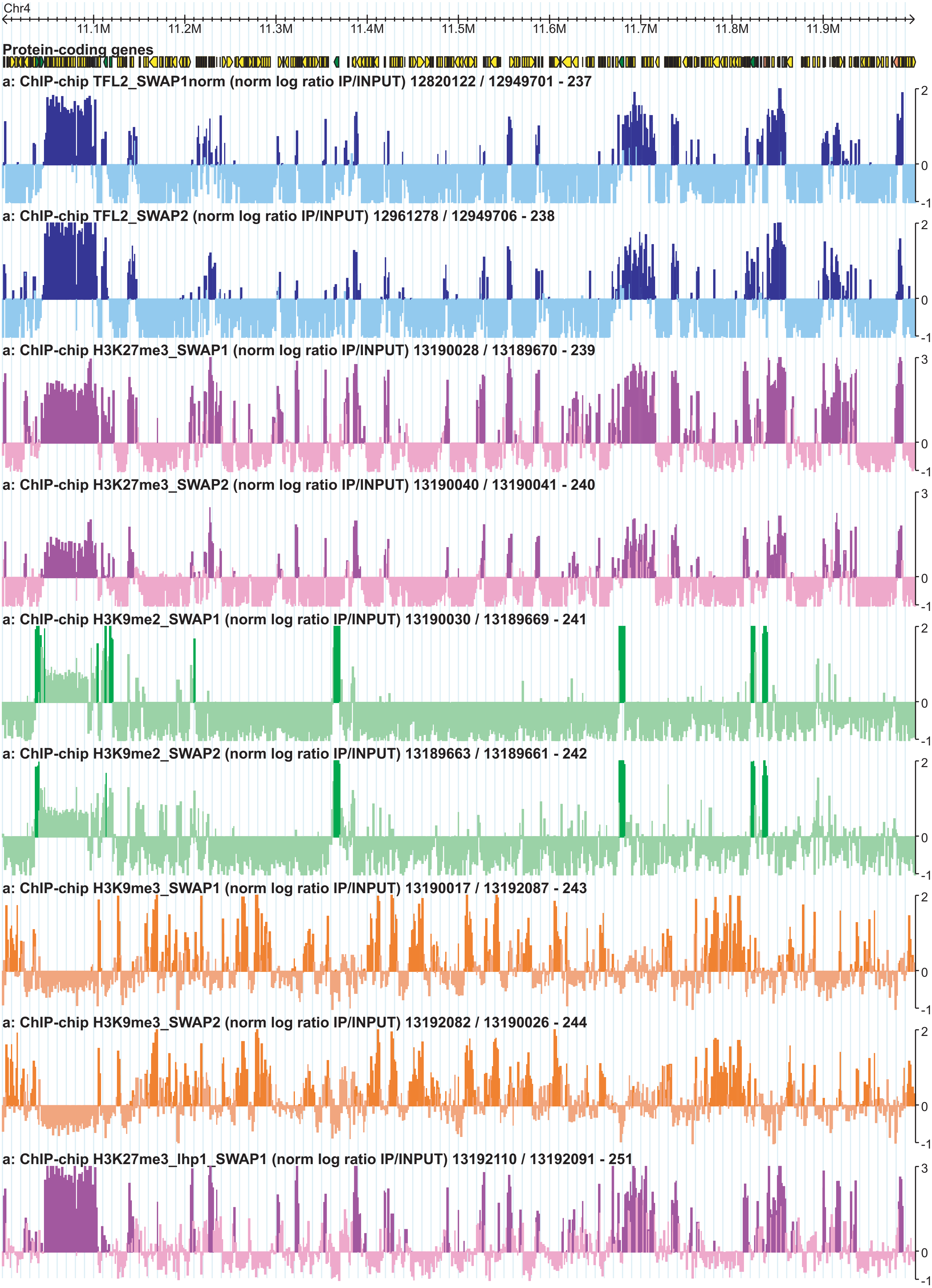

Chr4
11.1M
11.2M
11.3M
11.4M
11.5M
11.6M
11.7M
11.8M
11.9M
Protein-coding genes
a: ChIP-chip TFL2_SWAP1norm (norm log ratio IP/INPUT) 12820122 / 12949701 - 237
a: ChIP-chip TFL2_SWAP2 (norm log ratio IP/INPUT) 12961278 / 12949706 - 238
a: ChIP-chip H3K27me3_SWAP1 (norm log ratio IP/INPUT) 13190028 / 13189670 - 239
a: ChIP-chip H3K27me3_SWAP2 (norm log ratio IP/INPUT) 13190040 / 13190041 - 240
a: ChIP-chip H3K9me2_SWAP1 (norm log ratio IP/INPUT) 13190030 / 13189669 - 241
a: ChIP-chip H3K9me2_SWAP2 (norm log ratio IP/INPUT) 13189663 / 13189661 - 242
a: ChIP-chip H3K9me3_SWAP1 (norm log ratio IP/INPUT) 13190017 / 13192087 - 243
a: ChIP-chip H3K9me3_SWAP2 (norm log ratio IP/INPUT) 13192082 / 13190026 - 244
a: ChIP-chip H3K27me3_lhp1_SWAP1 (norm log ratio IP/INPUT) 13192110 / 13192091 - 251

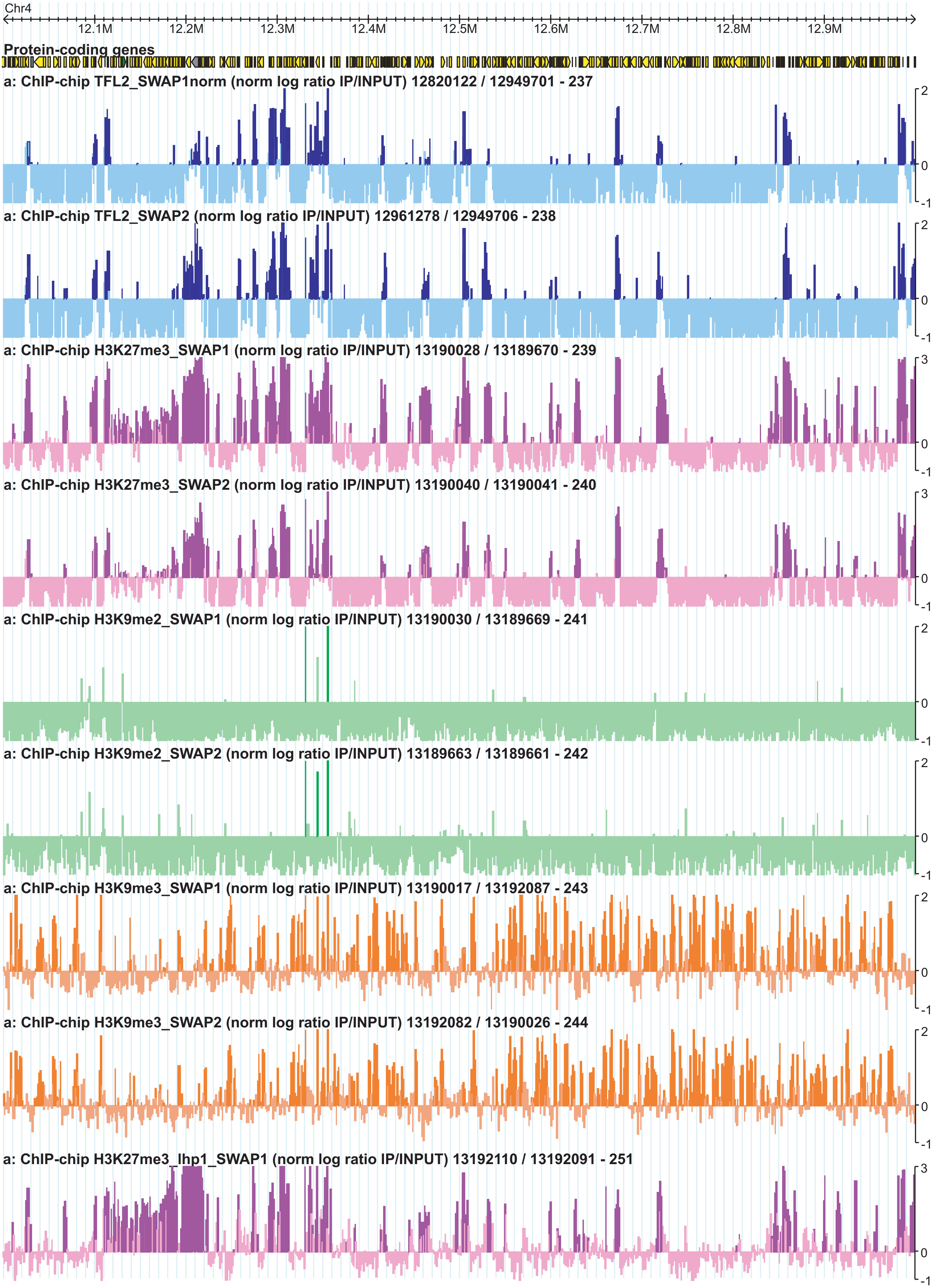
Chr4
12.1M
12.2M
12.3M
12.4M
12.5M
12.6M
12.7M
12.8M
12.9M
Protein-coding genes
a: ChIP-chip TFL2_SWAP1norm (norm log ratio IP/INPUT) 12820122 / 12949701 - 237
a: ChIP-chip TFL2_SWAP2 (norm log ratio IP/INPUT) 12961278 / 12949706 - 238
a: ChIP-chip H3K27me3_SWAP1 (norm log ratio IP/INPUT) 13190028 / 13189670 - 239
a: ChIP-chip H3K27me3_SWAP2 (norm log ratio IP/INPUT) 13190040 / 13190041 - 240
a: ChIP-chip H3K9me2_SWAP1 (norm log ratio IP/INPUT) 13190030 / 13189669 - 241
a: ChIP-chip H3K9me2_SWAP2 (norm log ratio IP/INPUT) 13189663 / 13189661 - 242
a: ChIP-chip H3K9me3_SWAP1 (norm log ratio IP/INPUT) 13190017 / 13192087 - 243
a: ChIP-chip H3K9me3_SWAP2 (norm log ratio IP/INPUT) 13192082 / 13190026 - 244
a: ChIP-chip H3K27me3_lhp1_SWAP1 (norm log ratio IP/INPUT) 13192110 / 13192091 - 251

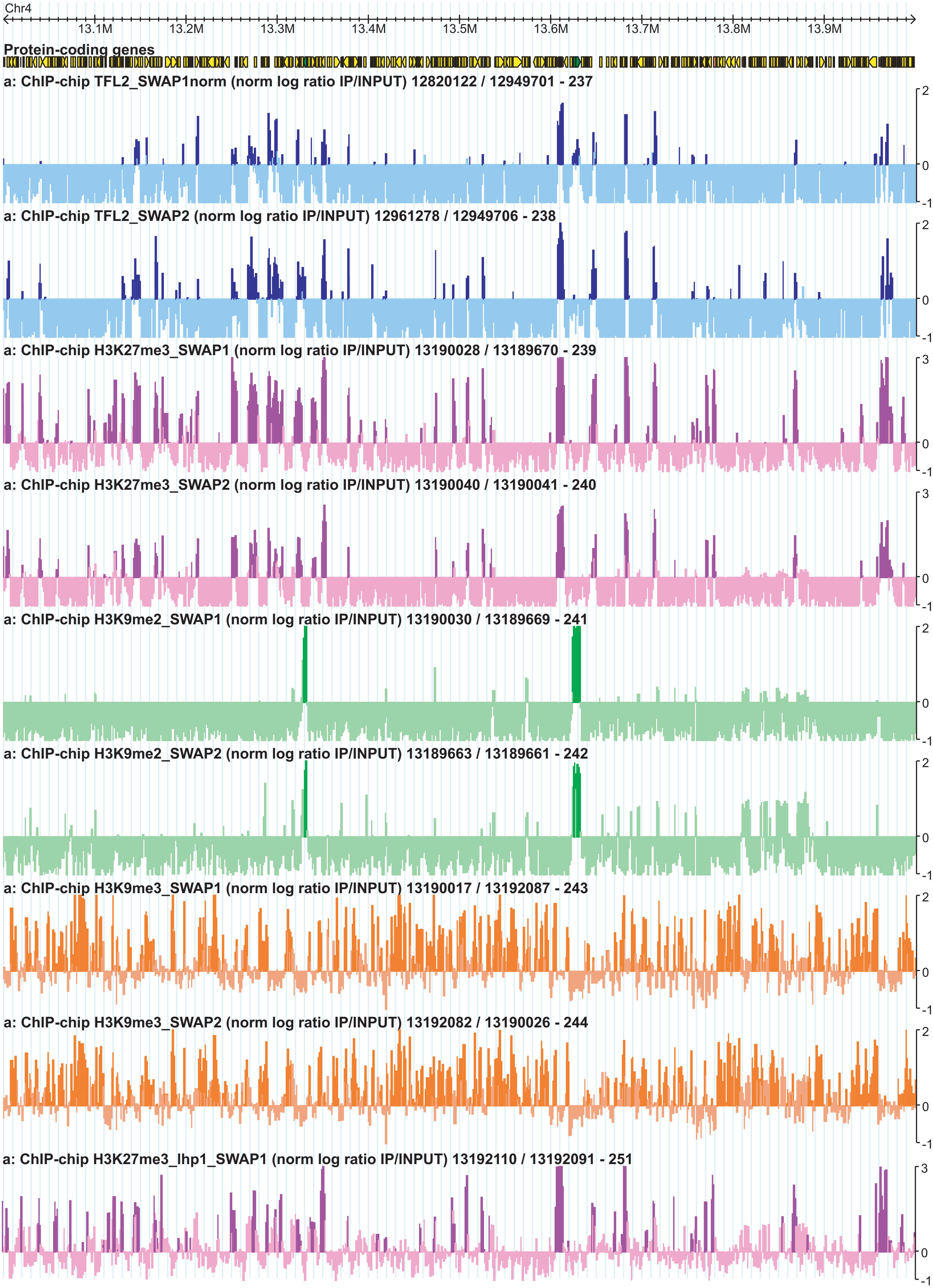

Chr4
13.1M
13.2M
13.3M
13.4M
13.5M
13.6M
13.7M
13.8M
13.9M
Protein-coding genes
a: ChIP-chip TFL2_SWAP1norm (norm log ratio IP/INPUT) 12820122 / 12949701 - 237
a: ChIP-chip TFL2_SWAP2 (norm log ratio IP/INPUT) 12961278 / 12949706 - 238
a: ChIP-chip H3K27me3_SWAP1 (norm log ratio IP/INPUT) 13190028 / 13189670 - 239
a: ChIP-chip H3K27me3_SWAP2 (norm log ratio IP/INPUT) 13190040 / 13190041 - 240
a: ChIP-chip H3K9me2_SWAP1 (norm log ratio IP/INPUT) 13190030 / 13189669 - 241
a: ChIP-chip H3K9me2_SWAP2 (norm log ratio IP/INPUT) 13189663 / 13189661 - 242
a: ChIP-chip H3K9me3_SWAP1 (norm log ratio IP/INPUT) 13190017 / 13192087 - 243
a: ChIP-chip H3K9me3_SWAP2 (norm log ratio IP/INPUT) 13192082 / 13190026 - 244
a: ChIP-chip H3K27me3_lhp1_SWAP1 (norm log ratio IP/INPUT) 13192110 / 13192091 - 251

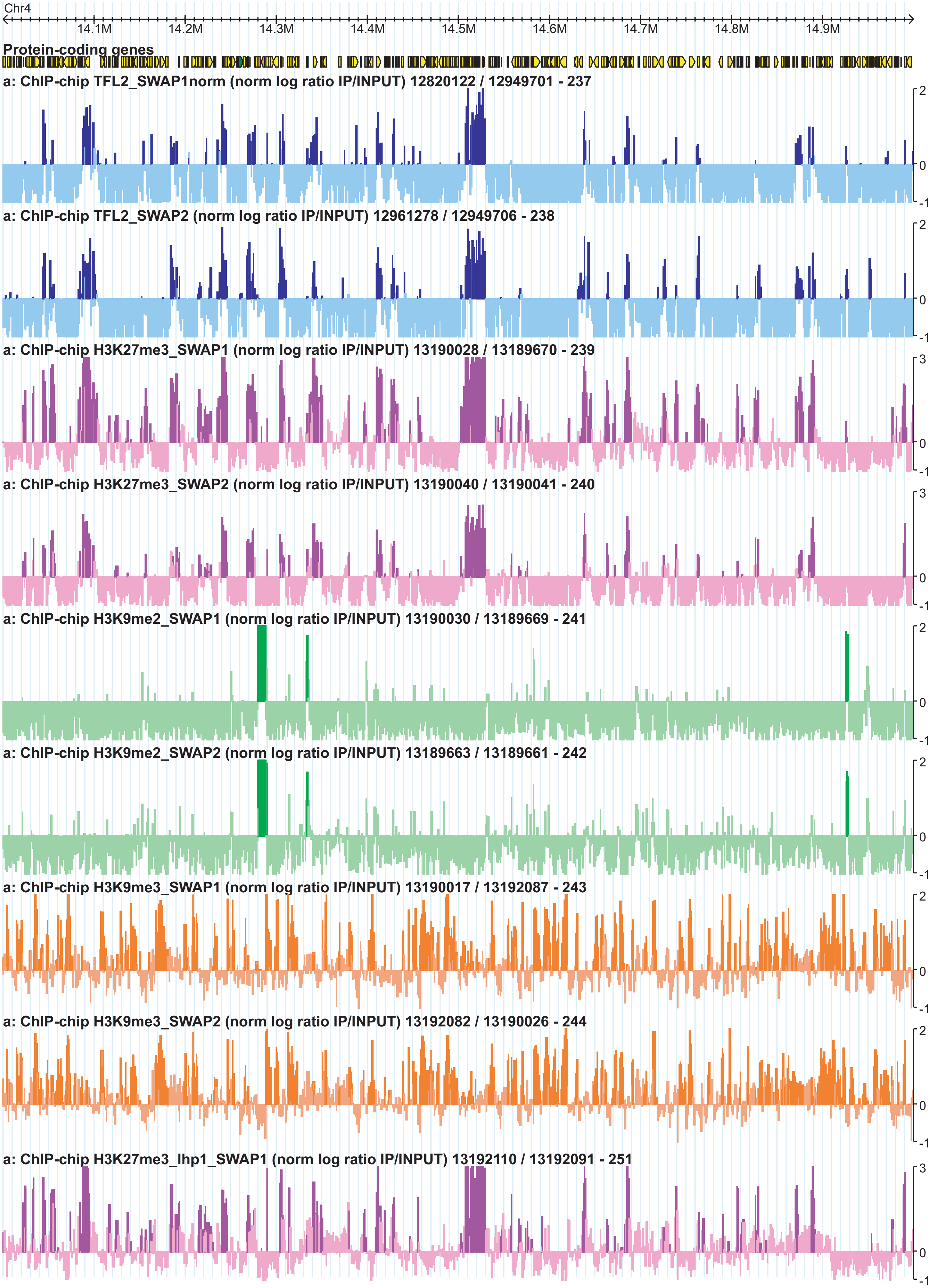
Chr4
14.1M
14.2M
14.3M
14.4M
14.5M
14.6M
14.7M
14.8M
14.9M
Protein-coding genes
a: ChIP-chip TFL2_SWAP1norm (norm log ratio IP/INPUT) 12820122 / 12949701 - 237
a: ChIP-chip TFL2_SWAP2 (norm log ratio IP/INPUT) 12961278 / 12949706 - 238
a: ChIP-chip H3K27me3_SWAP1 (norm log ratio IP/INPUT) 13190028 / 13189670 - 239
a: ChIP-chip H3K27me3_SWAP2 (norm log ratio IP/INPUT) 13190040 / 13190041 - 240
a: ChIP-chip H3K9me2_SWAP1 (norm log ratio IP/INPUT) 13190030 / 13189669 - 241
a: ChIP-chip H3K9me2_SWAP2 (norm log ratio IP/INPUT) 13189663 / 13189661 - 242
a: ChIP-chip H3K9me3_SWAP1 (norm log ratio IP/INPUT) 13190017 / 13192087 - 243
a: ChIP-chip H3K9me3_SWAP2 (norm log ratio IP/INPUT) 13192082 / 13190026 - 244
a: ChIP-chip H3K27me3_lhp1_SWAP1 (norm log ratio IP/INPUT) 13192110 / 13192091 - 251

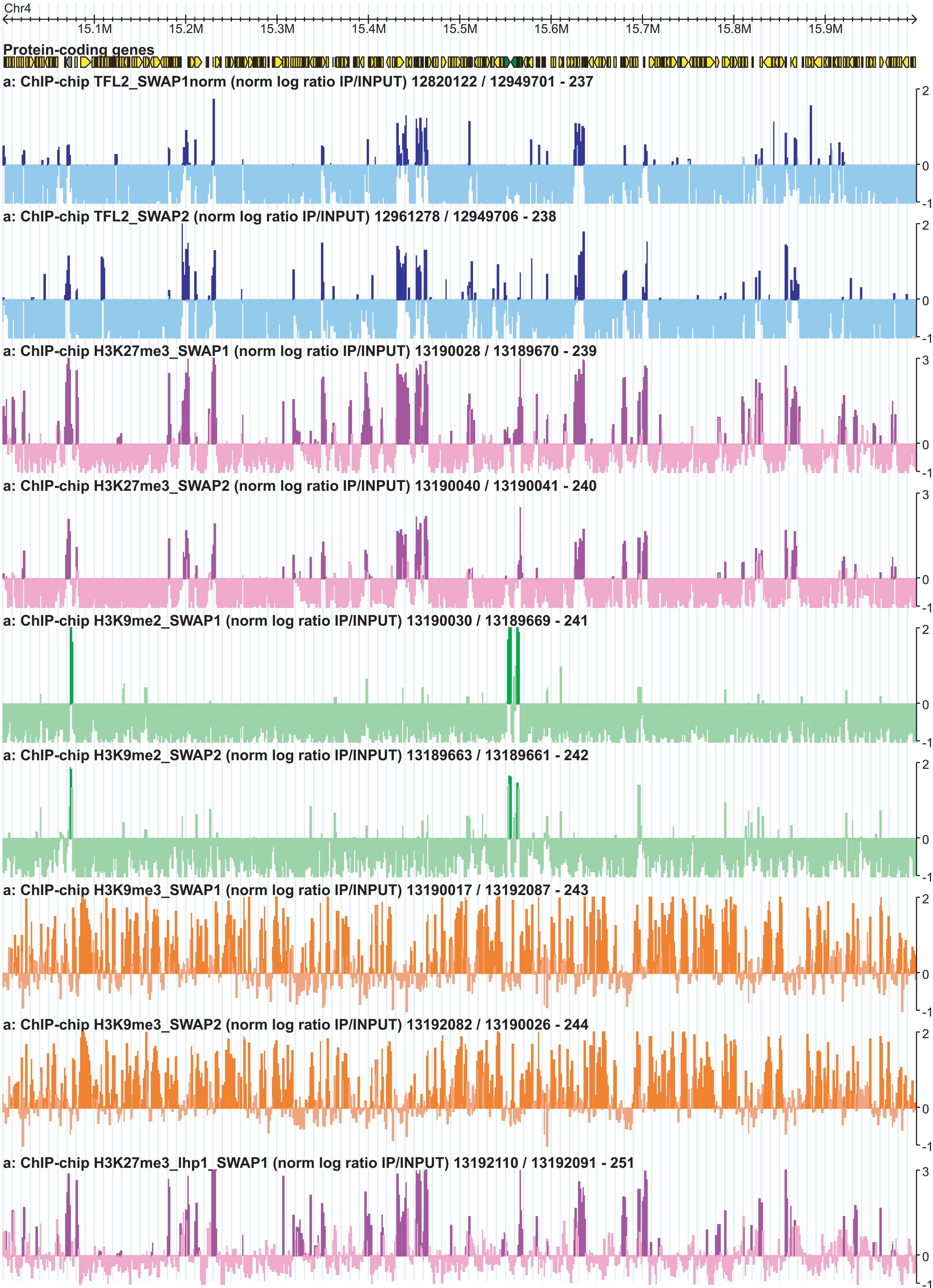
Chr4
15.1M
15.2M
15.3M
15.4M
15.5M
15.6M
15.7M
15.8M
15.9M
Protein-coding genes
a: ChIP-chip TFL2_SWAP1norm (norm log ratio IP/INPUT) 12820122 / 12949701 - 237
a: ChIP-chip TFL2_SWAP2 (norm log ratio IP/INPUT) 12961278 / 12949706 - 238
a: ChIP-chip H3K27me3_SWAP1 (norm log ratio IP/INPUT) 13190028 / 13189670 - 239
a: ChIP-chip H3K27me3_SWAP2 (norm log ratio IP/INPUT) 13190040 / 13190041 - 240
a: ChIP-chip H3K9me2_SWAP1 (norm log ratio IP/INPUT) 13190030 / 13189669 - 241
a: ChIP-chip H3K9me2_SWAP2 (norm log ratio IP/INPUT) 13189663 / 13189661 - 242
a: ChIP-chip H3K9me3_SWAP1 (norm log ratio IP/INPUT) 13190017 / 13192087 - 243
a: ChIP-chip H3K9me3_SWAP2 (norm log ratio IP/INPUT) 13192082 / 13190026 - 244
a: ChIP-chip H3K27me3_lhp1_SWAP1 (norm log ratio IP/INPUT) 13192110 / 13192091 - 251

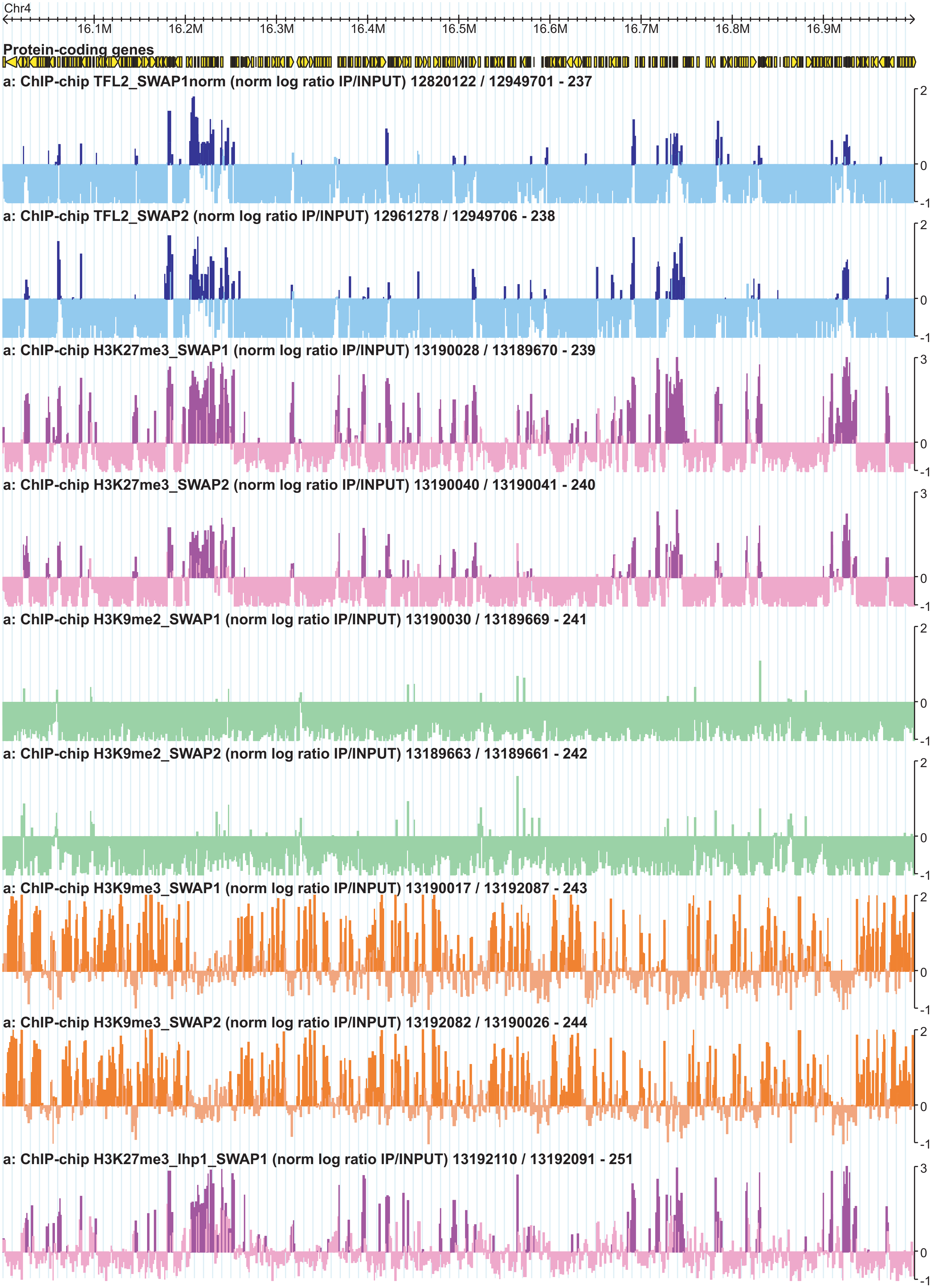
Chr4
16.1M
16.2M
16.3M
16.4M
16.5M
16.6M
16.7M
16.8M
16.9M
Protein-coding genes
a: ChIP-chip TFL2_SWAP1norm (norm log ratio IP/INPUT) 12820122 / 12949701 - 237
a: ChIP-chip TFL2_SWAP2 (norm log ratio IP/INPUT) 12961278 / 12949706 - 238
a: ChIP-chip H3K27me3_SWAP1 (norm log ratio IP/INPUT) 13190028 / 13189670 - 239
a: ChIP-chip H3K27me3_SWAP2 (norm log ratio IP/INPUT) 13190040 / 13190041 - 240
a: ChIP-chip H3K9me2_SWAP1 (norm log ratio IP/INPUT) 13190030 / 13189669 - 241
a: ChIP-chip H3K9me2_SWAP2 (norm log ratio IP/INPUT) 13189663 / 13189661 - 242
a: ChIP-chip H3K9me3_SWAP1 (norm log ratio IP/INPUT) 13190017 / 13192087 - 243
a: ChIP-chip H3K9me3_SWAP2 (norm log ratio IP/INPUT) 13192082 / 13190026 - 244
a: ChIP-chip H3K27me3_lhp1_SWAP1 (norm log ratio IP/INPUT) 13192110 / 13192091 - 251

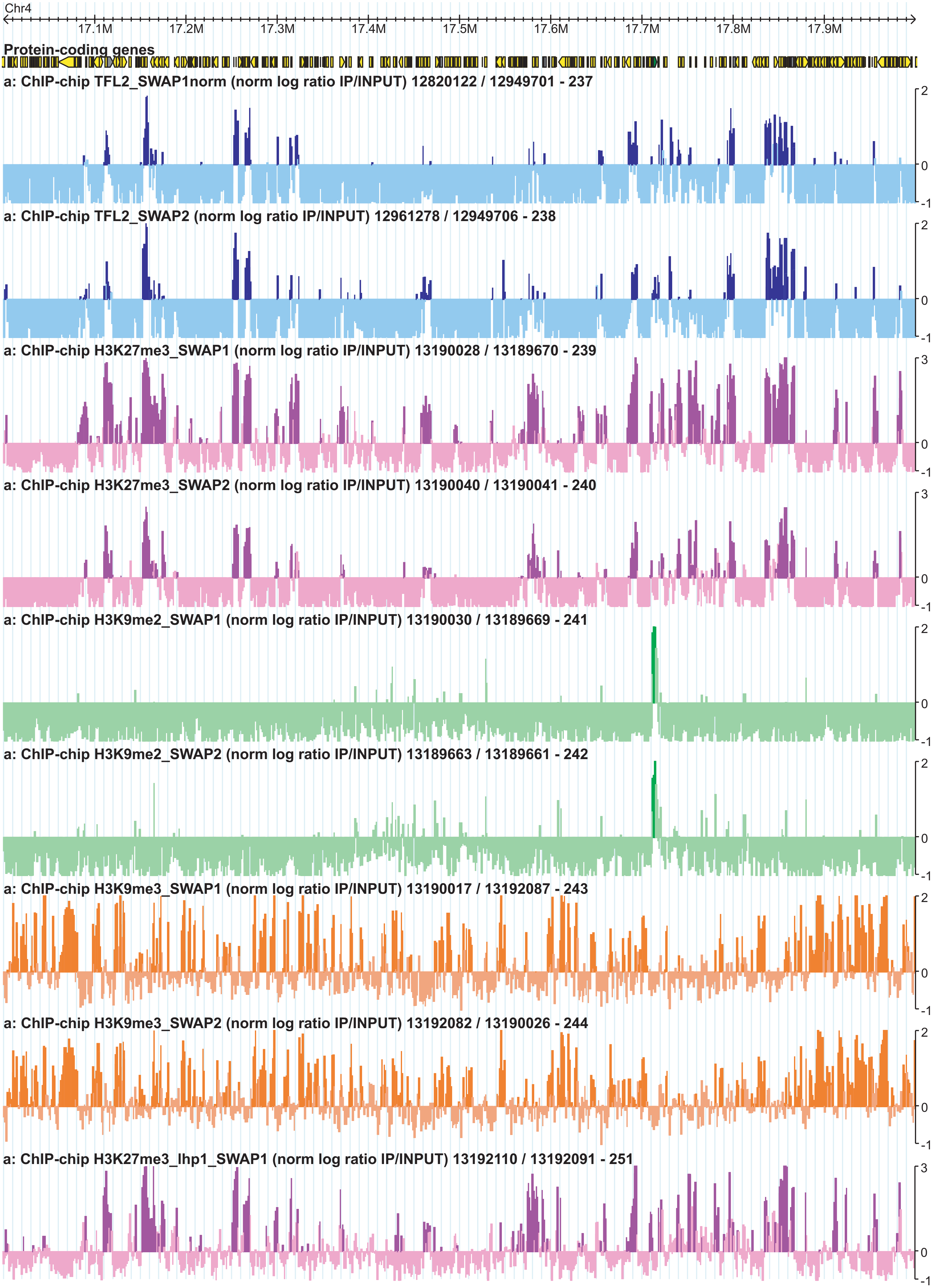

Chr4
17.1M
17.2M
17.3M
17.4M
17.5M
17.6M
17.7M
17.8M
17.9M
Protein-coding genes
a: ChIP-chip TFL2_SWAP1norm (norm log ratio IP/INPUT) 12820122 / 12949701 - 237
a: ChIP-chip TFL2_SWAP2 (norm log ratio IP/INPUT) 12961278 / 12949706 - 238
a: ChIP-chip H3K27me3_SWAP1 (norm log ratio IP/INPUT) 13190028 / 13189670 - 239
a: ChIP-chip H3K27me3_SWAP2 (norm log ratio IP/INPUT) 13190040 / 13190041 - 240
a: ChIP-chip H3K9me2_SWAP1 (norm log ratio IP/INPUT) 13190030 / 13189669 - 241
a: ChIP-chip H3K9me2_SWAP2 (norm log ratio IP/INPUT) 13189663 / 13189661 - 242
a: ChIP-chip H3K9me3_SWAP1 (norm log ratio IP/INPUT) 13190017 / 13192087 - 243
a: ChIP-chip H3K9me3_SWAP2 (norm log ratio IP/INPUT) 13192082 / 13190026 - 244
a: ChIP-chip H3K27me3_lhp1_SWAP1 (norm log ratio IP/INPUT) 13192110 / 13192091 - 251

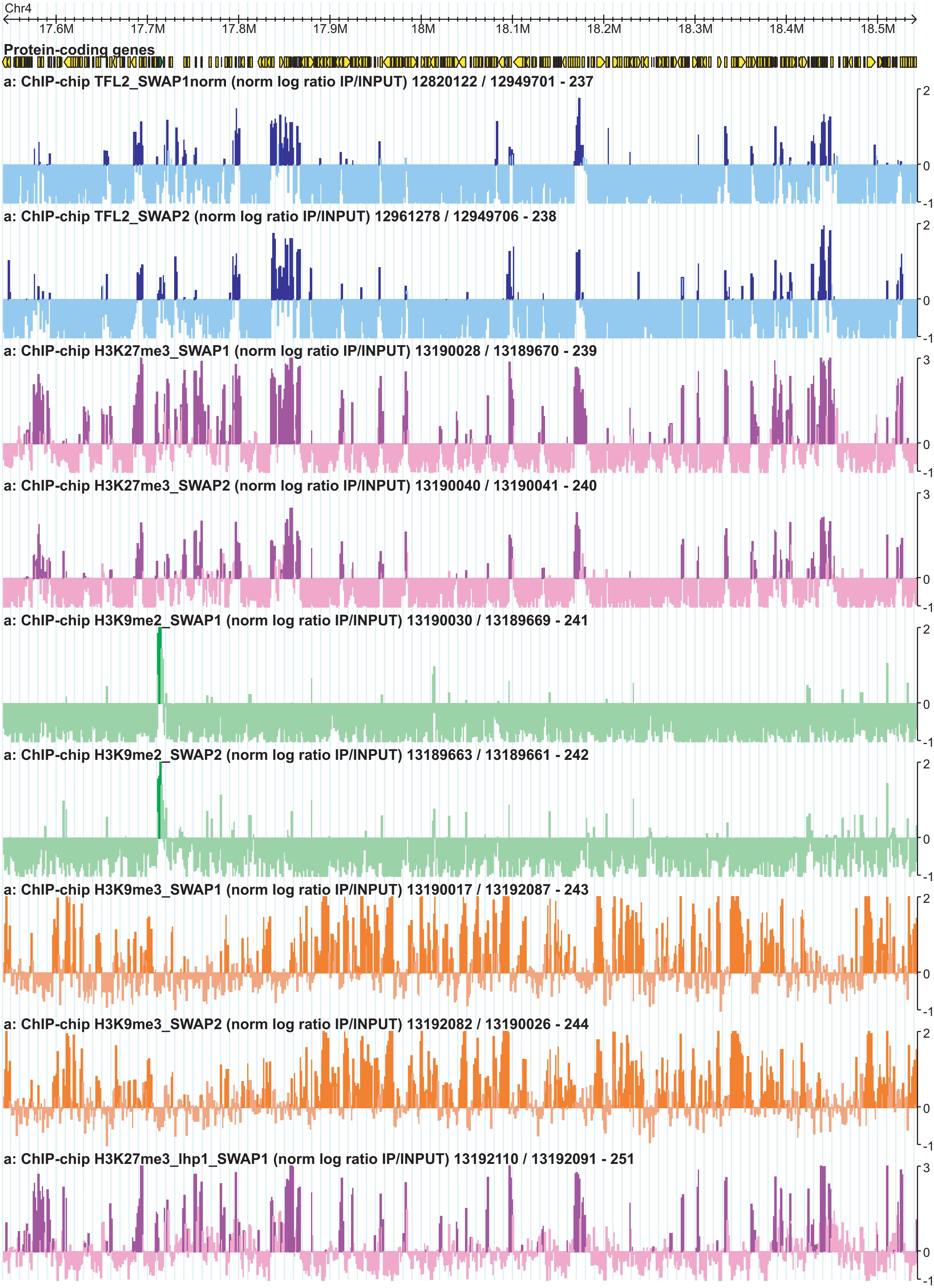

Chr4
17.6M
17.7M
17.8M
17.9M
18M
18.1M
18.2M
18.3M
18.4M
18.5M
Protein-coding genes
a: ChIP-chip TFL2_SWAP1norm (norm log ratio IP/INPUT) 12820122 / 12949701 - 237
2
0
-1
a: ChIP-chip TFL2_SWAP2 (norm log ratio IP/INPUT) 12961278 / 12949706 - 238
2
0
-1
a: ChIP-chip H3K27me3_SWAP1 (norm log ratio IP/INPUT) 13190028 / 13189670 - 239
3
0
-1
a: ChIP-chip H3K27me3_SWAP2 (norm log ratio IP/INPUT) 13190040 / 13190041 - 240
3
0
-1
a: ChIP-chip H3K9me2_SWAP1 (norm log ratio IP/INPUT) 13190030 / 13189669 - 241
2
0
-1
a: ChIP-chip H3K9me2_SWAP2 (norm log ratio IP/INPUT) 13189663 / 13189661 - 242
2
0
-1
a: ChIP-chip H3K9me3_SWAP1 (norm log ratio IP/INPUT) 13190017 / 13192087 - 243
2
0
-1
a: ChIP-chip H3K9me3_SWAP2 (norm log ratio IP/INPUT) 13192082 / 13190026 - 244
2
0
-1
a: ChIP-chip H3K27me3_lhp1_SWAP1 (norm log ratio IP/INPUT) 13192110 / 13192091 - 251
3
0
-1
