# Supplementary material for: Arabidopsis TFL2/LHP1 Specifically Associates with Genes Marked by Trimethylation of Histone H3 Lysine 27
Source: PLoS Genet. 2007 Jun 1;3(6):e86. doi: 10.1371/journal.pgen.0030086 (PMC1885283; doi:10.1371/journal.pgen.0030086)

Turck, Roudier et al. FIGURE S4

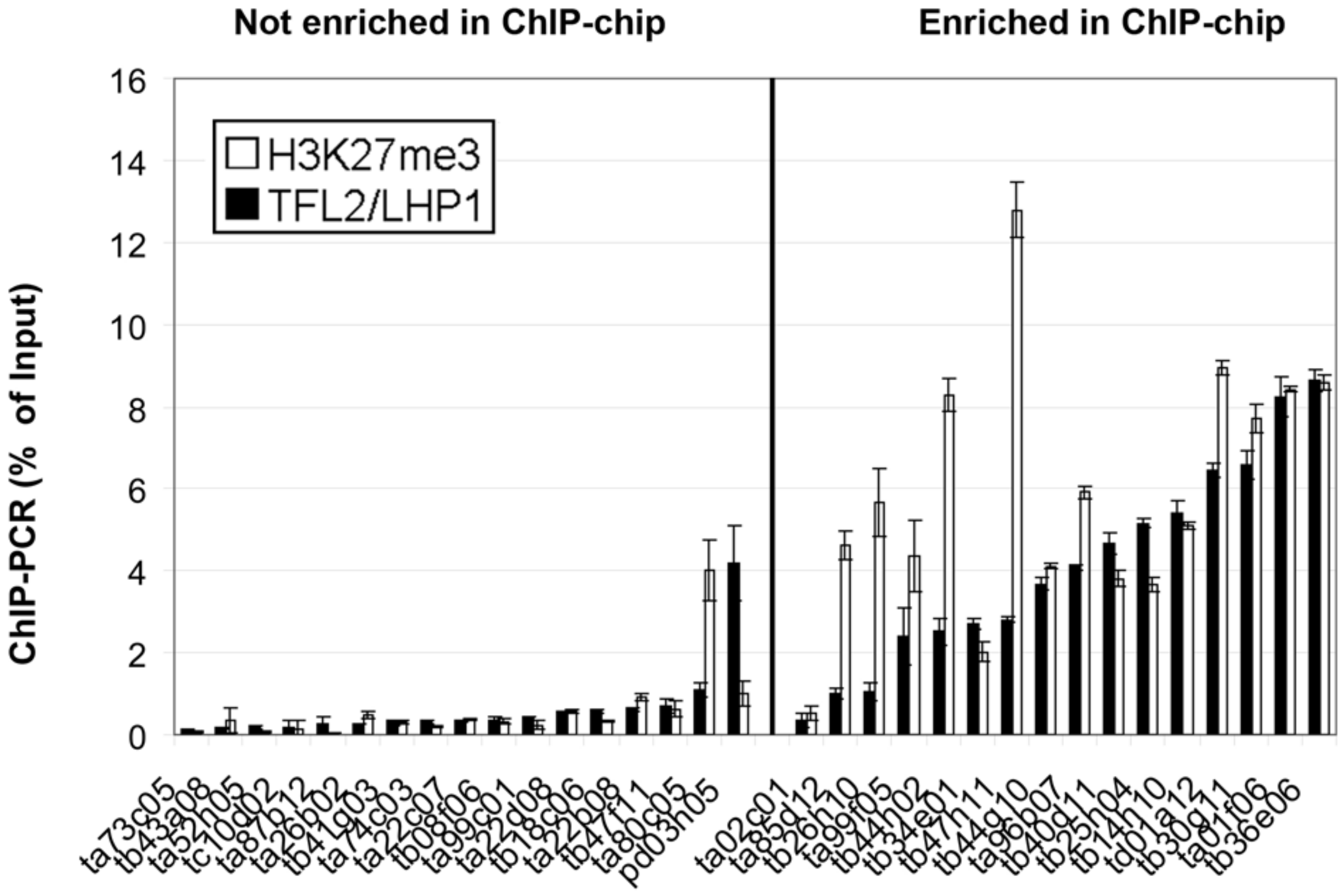

Supplement: Supplementary file 4 [file pgen.0030086.sg004.pdf]
