# Supplementary material for: Arabidopsis TFL2/LHP1 Specifically Associates with Genes Marked by Trimethylation of Histone H3 Lysine 27
Source: PLoS Genet. 2007 Jun 1;3(6):e86. doi: 10.1371/journal.pgen.0030086 (PMC1885283; doi:10.1371/journal.pgen.0030086)
Supplement: Supplementary file 5 [file pgen.0030086.st009.doc]

# **Turck_Roudier et al., Table S9**

|  | TFL2/LHP1  603 | H3K27me3  994 | H3K9me2  638 | H3K9me3  1732 |
| --- | --- | --- | --- | --- |
| TFL2/LHP1  603 | - | 525  52.76% | 38  5,96% | 50  2.88% |
| H3K27me3  994 | 525  87.06% | - | 12  1.88% | 103  5.94% |
| H3K9me2  638 | 38  6.12% | 12  1.20% | - | 9  0.52% |
| H3K9me3  1732 | 50  8.23% | 103  10.36% | 9  1.41% | - |

**Table S9.** **Extent of overlap between TFL2/LHP1 target genes and genes marked by H3K9me2, H3K9me3 or H3K27me3.**

Number of genes is indicated together with percentage of overlap. As explained in the text, the lower percentage of overlap between H3K27me3-marked genes and TFL2/LHP1 target genes relative to the reciprocal comparison is mainly caused by a higher signal to noise ratio in the case of H3K27me3, which leads to H3K27me3 domains appearing slightly larger (and thus sometimes covering additional genes) in comparison to the corresponding TFL2/LHP1 domains (for an example, see Figure 5C). Among the limited number of genes (42) that are targeted by TFL2/LHP1 and apparently co-marked by H3K9me3 and H3K27me3, all but 3 correspond in fact to TFL2/LHP1-H3K27me3 domains that are juxtaposed to H3K9me3 domains, with less than 25% sequence overlap between the two. Thus most overlaps between H3K27me3 and H3K9me3 simply reflect the tight juxtaposition of domains rather than any true co-localization.
