# Supplementary material for: Arabidopsis TFL2/LHP1 Specifically Associates with Genes Marked by Trimethylation of Histone H3 Lysine 27
Source: PLoS Genet. 2007 Jun 1;3(6):e86. doi: 10.1371/journal.pgen.0030086 (PMC1885283; doi:10.1371/journal.pgen.0030086)
Supplement: Supplementary file 6 [file pgen.0030086.st011.doc]

Turck_Roudier et al. Table S11

Number of tiles reporting an enriched IP/INPUT ratio and intersection between replicates.

|  | Replicate1 | Replicate2 | Intersection * | Overlap level |
| --- | --- | --- | --- | --- |
| TFL2/LHP1 | 2294 | 3633 | 2175 (1713) | 94.81% |
| H3K27me3 | 5336 | 3099 | 3077 (2775) | 99.29% |
| H3K9me2 | 2968 | 2834 | 2732 (2589) | 96.40% |
| H3K9me3 | 5733 | 6528 | 5233 (4453) | 91.27% |

* Numbers in brackets refer to tiles that remain after manual curation to remove singletons.
